# Dimensionality reduction beyond neural subspaces with slice tensor component analysis

**DOI:** 10.1038/s41593-024-01626-2

**Published:** 2024-05-06

**Authors:** Arthur Pellegrino, Heike Stein, N. Alex Cayco-Gajic

**Affiliations:** 1 Laboratoire de Neurosciences Cognitives et Computationnelles, INSERM U960, Département D’Etudes Cognitives, Ecole Normale Supérieure, PSL University, Paris, France; 2 Institute for Adaptive and Neural Computation, School of Informatics, University of Edinburgh, Edinburgh, UK

**Keywords:** Neural decoding, Neural circuits

## Abstract

Recent work has argued that large-scale neural recordings are often well described by patterns of coactivation across neurons. Yet the view that neural variability is constrained to a fixed, low-dimensional subspace may overlook higher-dimensional structure, including stereotyped neural sequences or slowly evolving latent spaces. Here we argue that task-relevant variability in neural data can also cofluctuate over trials or time, defining distinct ‘covariability classes’ that may co-occur within the same dataset. To demix these covariability classes, we develop sliceTCA (slice tensor component analysis), a new unsupervised dimensionality reduction method for neural data tensors. In three example datasets, including motor cortical activity during a classic reaching task in primates and recent multiregion recordings in mice, we show that sliceTCA can capture more task-relevant structure in neural data using fewer components than traditional methods. Overall, our theoretical framework extends the classic view of low-dimensional population activity by incorporating additional classes of latent variables capturing higher-dimensional structure.

## Abstract (web summary)

Neural activity does not always lie in a low-dimensional subspace. The authors extend this classic view to show that task-relevant information is distributed across multiple covariability classes and propose a new method, sliceTCA, to disentangle them.

## Main

Neural activity varies in relation to fluctuations in the environment, changes in synaptic input, learning or adaptation, and heterogeneous cell properties, creating variability across neurons, time and trials. Recent work has emphasized that trial-to-trial variability is often correlated across populations of neurons^1^, generating low-dimensional representations of sensory or behavioral variables. Indeed, analyzing the structure of neural covariances has led to key insights into the information encoded and computations performed by neural circuits^2,3^. Such findings have driven an increase in the popularity of dimensionality reduction methods (for example, principal component analysis (PCA)), which seek to capture structure in neural data by identifying population-wide patterns of covariance. More recent work has advocated instead for applying tensor-based methods (for example, tensor component analysis (TCA)) that distinguish between changes in neural trajectories that occur over fast (within-trial) and slow (between-trial) timescales^4–6^. In these approaches, neural activity is assumed to be constrained to a low-dimensional neural subspace (defined by a set of latent variables) that is fixed over the course of an experiment.

However, this picture of low-dimensional latent variables fails to account for some forms of structure in neural datasets. First, not all population activity is described by covariance patterns across neurons. For example, many brain areas produce temporal sequences in which the latency of activation varies from neuron to neuron but that are highly stereotyped across conditions^7–11^. Second, the neural encoding weights for a given sensory stimulus may change over trials due to adaptation, learning^12,13^ or representational drift^14–16^. These examples demonstrate three different types (or ‘classes’) of ‘covariability’, by which we mean structure in neural population recordings that can be described by stereotyped patterns across neurons, trials or time. Yet, because common neural dimensionality reduction methods typically look for covarying population-wide patterns, they may miss these additional forms of covariability in neural data.

Here, we propose that neural circuits are likely to encode task-relevant information in multiple co-occurring covariability classes. To demonstrate this, we introduce sliceTCA, a new unsupervised dimensionality reduction method able to identify and disentangle components belonging to different covariability classes that are mixed within the same dataset. This property contrasts sliceTCA from matrix factorization methods (such as PCA), which capture a single covariability class at a time, and from TCA, which identifies components constrained to all of them simultaneously. As a result, we show that sliceTCA can capture more structure in fewer components than either of these methods. Based on theoretical and practical considerations of the sliceTCA decomposition, we develop an analysis pipeline for model selection, optimization and visualization that is implemented in a readily applicable Python library.

After validating our method on simulated data, we illustrate the advantages of the mixed covariability framework in three large-scale neural datasets. First, we demonstrate that different covariability classes encode distinct behaviorally relevant signals in motor cortical recordings in nonhuman primates^17^. Next, in simultaneous imaging data from cortical and cerebellar populations during a cued motor task^18^, we show that sliceTCA uncovers task-relevant manifolds by considering covariability across trials. Finally, we analyze a recent dataset from the International Brain Laboratory (IBL)^19^ and show that sliceTCA disentangles region-specific covariability classes across the visual cortex, hippocampus, thalamus and midbrain. We then provide a geometric intuition for how neural population activity is shaped by latent variables belonging to the three different covariability classes. Together, these results demonstrate the necessity of extending the traditional view of latent variables and neural covariability to uncover higher-dimensional latent structure. With sliceTCA, we propose a new unsupervised dimensionality reduction method that uncovers coexisting classes of behaviorally relevant covariability in neural datasets.

## Results

### Multiple covariability classes

**Fig. 1:**
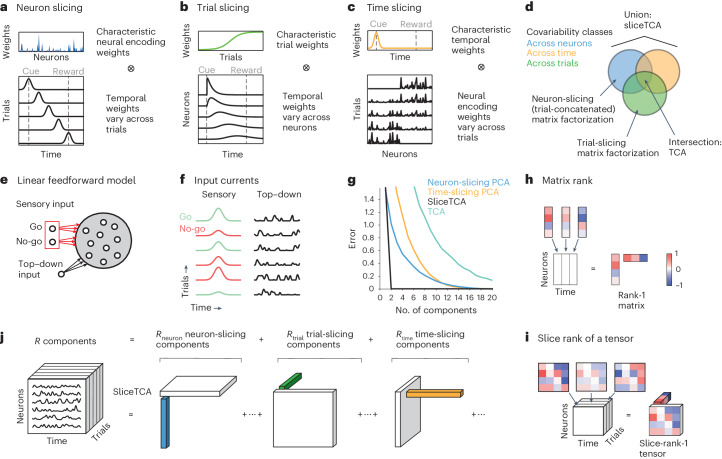
SliceTCA demixes covariability across neurons, time and trials. **a**, Example of a latent variable that represents a fixed neural encoding but whose temporal profile changes from trial to trial. **b**, Example of a latent variable that scales in amplitude over trials but has a neuron-specific time course within a trial. **c**, Example of a latent variable with a characteristic temporal profile within a trial but whose neural encoding weights change over trials. **d**, Schematic of the three covariability classes captured by sliceTCA. Matrix factorization methods such as PCA capture only a single covariability class at a time depending on how the data tensor is unfolded into matrix form. Because TCA treats neurons, trials and time symmetrically, it requires each component to lie in the intersection of the three classes. In contrast, sliceTCA represents the union of these three classes. **e**, Toy model of perceptual learning during a go/no-go task. On each trial, a population of linear neurons receives (1) a sensory input from one of two upstream sources representing the go/no-go stimuli and (2) top–down modulation representing stimulus-independent factors. Red indicates plastic weights. **f**, Evolution of inputs over trials. Go/no-go inputs increase/decrease in strength over trials due to synaptic potentiation/depression, whereas top–down inputs vary from trial to trial but are nonplastic. **g**, Error as a function of the number of components for different methods. **h**, Schematic of a rank-1 matrix. Each column of the matrix is a scaled version of the same vector. Equivalently, the matrix can be written as the outer product of that same column vector and a row vector representing the scaling weights. **i**, Schematic of a slice-rank-1 tensor. Each ‘slice’ of the tensor is a scaled version of the same matrix. The tensor can be written as an outer product of that matrix (a ‘slice’) and a vector representing the scaling weights. **j**, Schematic illustrating that sliceTCA approximates the data tensor as a low-slice-rank approximation. Each component is a slice-rank-1 tensor, which can be one of three types: neuron slicing, trial slicing or time slicing, corresponding to the examples in **a**–**c**.

**Extended Data Fig. 1:**
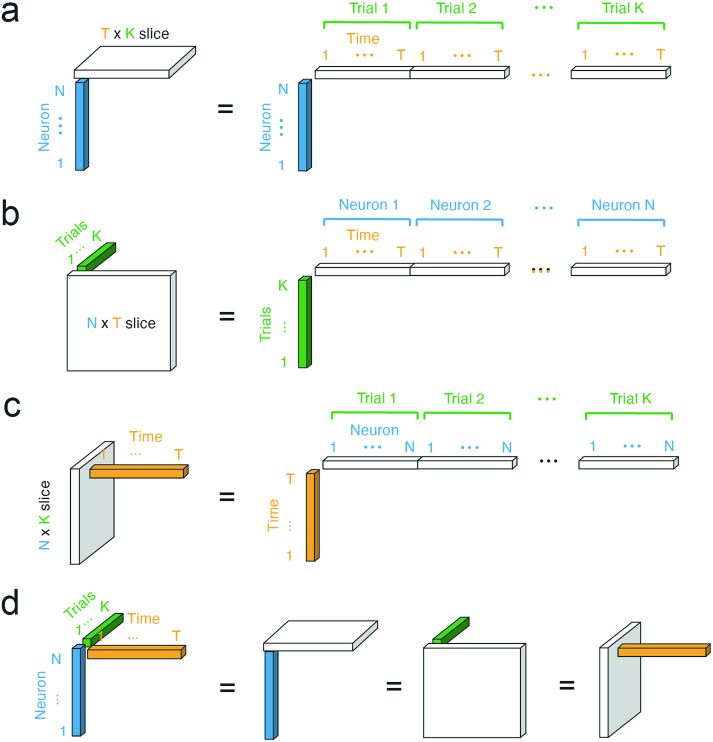
Tensor decomposition, matrix factorization, and covariability classes. **a**. A neuron-slicing component can be converted into a rank-1 matrix by unfolding the slice into a row vector (or equivalently, by unfolding the reconstructed 3-tensor into a matrix). A sliceTCA model comprised only of neuron-slicing components (that is, *R*_time_ = *R*_trial_ = 0) is equivalent to applying matrix factorization after unfolding the data tensor into an *N* × *KT* matrix (for example, as in ‘trial-concatenated PCA’). Therefore, a neuron-slicing component can be interpreted as a latent trajectory that varies over trials but which has fixed neural encoding weights. **b-c**. The trial-slicing (**b**) and time-slicing (**c**) components can similarly be converted to rank-1 matrices. These components represent covariability across trials and time points, respectively. **d**. A TCA component can be represented as any of the three sliceTCA components by defining the slice to be the outer product of two of the loading vectors. This means that a single TCA component simultaneously corresponds to covariability across neurons, time, and trials.

Neural activity often displays correlated fluctuations^1^. This form of covariability in neural data is usually determined by the neuron-by-neuron covariance matrix. Classic methods such as PCA capture the neural covariance matrix to identify characteristic patterns of neural weights whose time course of activation can vary freely from trial to trial (Fig. 1a). Each of these patterns is represented by a different component. However, there are other forms of structure in population activity that are not captured by the neural covariance matrix and that would be discarded within this framework. Heterogeneous latencies or timescales in different neurons (Fig. 1b) have been widely reported across brain regions, including in neural sequences^7–11^. Such temporal patterns are often characteristic for trials of the same task condition. Hence, such patterns represent a distinct kind of covariability in neural data, in which population activity covaries over trials, whereas the time courses of activation are heterogeneous across neurons (Fig. 1b and Extended Data Fig. 1).

These two examples illustrate that population covariability falls into multiple classes, which we call ‘neural covariability’ and ‘trial covariability’. Similar to neural covariability, trial covariability can be analyzed using the trial-by-trial covariance matrix, each element of which describes the similarity between the time courses of the full population response on two distinct trials. Indeed, using this approach, previous work has argued that different cortical regions are better described by trial covariability or neural covariability^7^. An additional form of structure in population activity might follow a characteristic temporal profile (say, locked to the time of stimulus presentation), whereas its neural encoding profile might change from trial to trial (Fig. 1c) due to adaptation or representational drift^12,14–16^. This gives rise to a third covariability class corresponding to ‘temporal covariability’, which is captured by the time-by-time covariance matrix.

**Extended Data Fig. 2:**
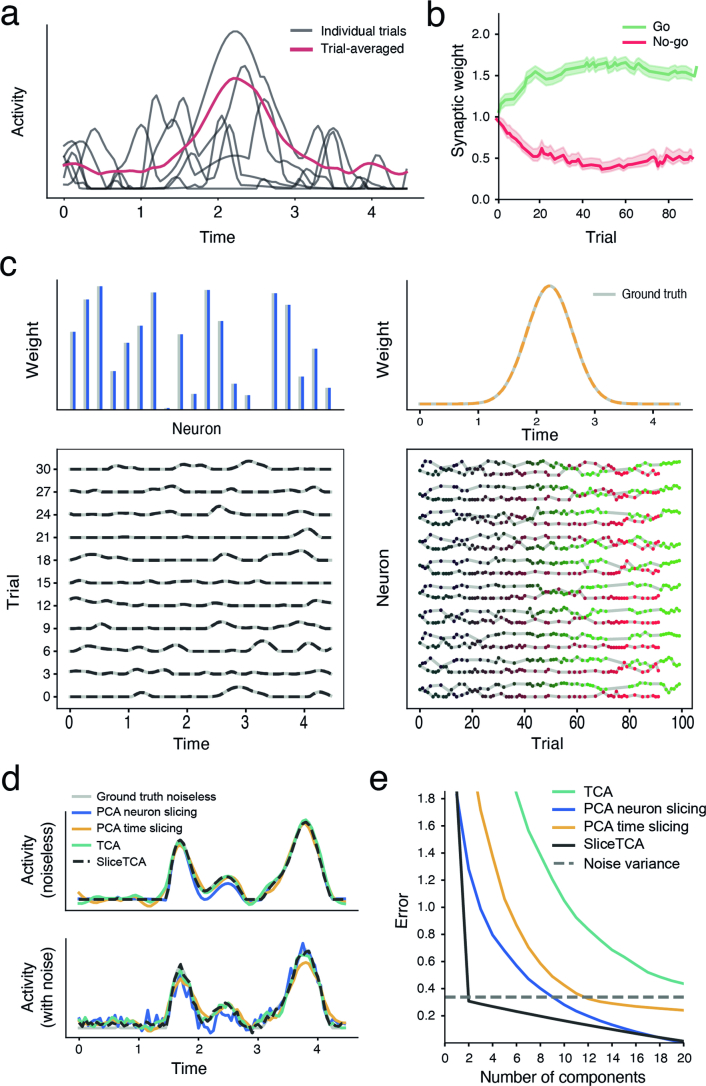
Feedforward model of perceptual learning. **a**. Sample of the activity of a single neuron over six example trials (grey) as well as trial-averaged activity (red). **b**. Evolution of the mean Go and No-go weight over learning. Shading represents the standard error of the mean. **c**. Recovered neuron-slicing (left) and time-slicing (right) components, plotted alongside ground truth values (grey). Weights for each neuron in the slice of the time-slicing component are plotted separately for Go (green) and No-go (red) trials. The sliceTCA decomposition captures the ground truth exactly. **d**. Single neuron activity is better captured by sliceTCA with (bottom) and without (top) white noise added to the data tensor. **e**. Mean squared error of decomposing the activity of the feedforward model with added white noise for sliceTCA, TCA, and PCA as a function of the number of components used in the model. The dashed line represents the mean squared deviation between the noisy and the noiseless model. Adding white noise does not affect the performance of sliceTCA relative to TCA or PCA.

In this Article, we argue that neural population activity is likely to exhibit multiple covariability classes that are intermixed (Fig. 1d). To provide intuition on how mixed covariability could arise at the level of neural circuits, we first built a toy feedforward model of the sensory cortex during a go/no-go task (Fig. 1e). In this model, a population of linear cortical neurons received two sources of input in the context of a go/no-go task (Fig. 1f and Extended Data Fig. 2). First, all neurons received a sensory input that was time-locked to the stimulus. The projection weights were stimulus specific (either go or no-go) and plastic (potentiation/depression for go/no-go stimuli, respectively), in line with enhanced sensitivity to target stimuli in the sensory cortex during perceptual learning^20,21^. Potentiation and depression rates were stochastic and heterogeneous across neurons (Extended Data Fig. 2b,c and Methods). Second, all neurons also received a top–down modulatory input unrelated to task events, for example, due to temporal fluctuations of arousal^22^. In this linear model, each neuron’s activity is simply the summation of its sensory and top–down input currents (Fig. 1f).

From these minimal assumptions, the two input sources represent two different classes of covariability. First, stimulus-locked sensory inputs share the same characteristic temporal profile, with neural encoding weights that vary over neurons and trials due to heterogeneity in potentiation and depression rates. This type of structure corresponds to time covariability (Fig. 1c). In contrast, the top–down input is fed through a static pattern of neural weights (as these synapses are nonplastic) but fluctuates in strength over time and trials due to variability in the modulatory signals. Therefore, this second type of structure falls into the class of neural covariability (Fig. 1a). In principle, the resulting population activity can be described by two components capturing the sensory and top–down inputs (Methods). Yet, despite the simplicity of this model, PCA, which relies on a single covariability class, requires many components to capture the resulting population activity (Fig. 1g). This toy model illustrates how mixed covariability classes can emerge from minimal assumptions regarding different sources of heterogeneity in neural circuits and that they cannot be disentangled using traditional dimensionality reduction methods.

### SliceTCA disentangles mixed covariability

To disentangle mixed covariability in neural data, we must first return to the mathematical formulation of PCA. Matrix factorization methods, including PCA and non-negative matrix factorization (NMF), approximate a data matrix *X* as a sum of *R* components:

1 $${{{{X}}}}\approx \hat{{{{{X}}}}}=\mathop{\sum }\limits_{r=1}^{R}{{{{{X}}}}}^{\,(r)}.$$

In neuroscience, *X* is generally a matrix of size *N* × *KT* containing the activity of *N* neurons recorded over *K* trials, each containing *T* time points. Each component *X*^(*r*)^ is a rank-1 matrix defined by a vector of neural weights describing different activation patterns across the population and a vector of temporal weights describing how the strength of these patterns changes in amplitude over the course of the experiment (Fig. 1h). Through this low-rank constraint, these methods are typically used to capture dominant patterns of neural covariability.

However, arranging neural data into matrix form limits the structure that can be captured, as matrix factorizations do not distinguish between rapid fluctuations within a trial and slower variations across trials^4^. This limitation can be addressed by structuring the data into an *N* × *T* × *K* tensor, which can be similarly decomposed following equation (1) into a low-rank tensor approximation. For this, we must generalize the concept of a rank-1 matrix to tensors. Different definitions of the tensor rank will capture different forms of structure in the data.

Here, we present sliceTCA, a new tensor decomposition method based on the slice rank^23^ (Methods). A rank-1 matrix is defined as the outer product of two vectors so that each column of the matrix is a scaled version of the same column vector (Fig. 1h). Similarly, a slice-rank-1 tensor is defined as the outer product of a vector and a matrix (or ‘slice’; Fig. 1i). Depending on how the tensor is sliced, these components can capture any of the three covariability classes.

To see this, we may consider each slice type separately. First, a neuron-slicing component is described by a vector of characteristic neural weights and a matrix describing the time course for that component over trials (Fig. 1a). This is the same class of neural covariability captured by common applications of matrix factorizations in which the data tensor is reshaped or ‘unfolded’ into an *N* × *KT* matrix (sometimes referred to as ‘trial-concatenated’ matrix factorization; Extended Data Fig. 1a). Similarly, the trial-slicing components capture trial covariability: stereotyped neuron-specific temporal profiles that vary together in amplitude over trials (Fig. 1b). Meanwhile, the time-slicing components identify time covariability: a common temporal profile whose neural encoding weights change from trial to trial, for example, due to learning, adaptation or drift (Fig. 1c).

If only one of these three slice types were fitted, sliceTCA would be equivalent to a matrix factorization on the respective unfolding of the data tensor (Extended Data Fig. 1a–c). Indeed, previous work has argued for performing PCA on different unfoldings of the data tensor to identify the slice type that provides the best approximation^7^. Crucially, sliceTCA differs from this approach by fitting all three slice types simultaneously, thereby demixing different covariability classes that may be combined within the same dataset (Fig. 1j). SliceTCA is also related to, yet distinct from, TCA (that is, the CP (canonical polyadic) decomposition)^4–6^. TCA constrains each component to be described by the outer product of three vectors of neural, trial and temporal factors, which requires that each component lies in the intersection of all three covariability classes (Fig. 1d and Methods). To demonstrate the conceptual difference between these methods, we applied sliceTCA to our toy model (Fig. 1e). Indeed, sliceTCA was able to decompose the activity into its two ground-truth components (Extended Data Fig. 2c), whereas PCA and TCA required substantially more components to capture the data (Fig. 1g). SliceTCA also outperformed PCA and TCA in the presence of noise (Extended Data Fig. 2d,e and Supplementary Fig. 1). These results demonstrate that, by disentangling mixed covariability classes, sliceTCA is able to capture more structure in the data with fewer components as compared to other methods.

**Extended Data Fig. 3:**
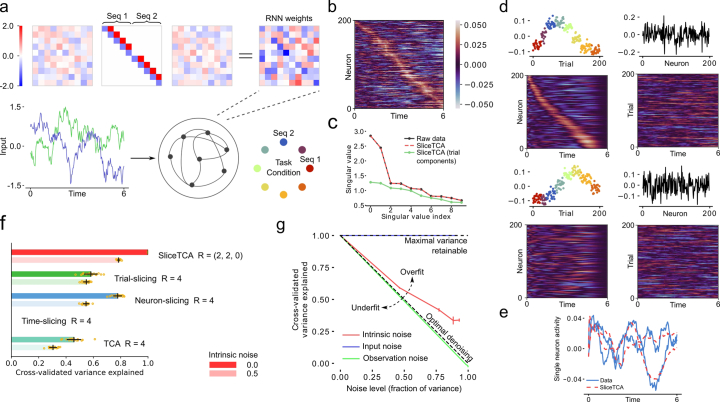
Linear RNN model of condition-specific neural sequences. **a**. Top: Two sequences are encoded into neural weights using the Schur decomposition (24). Bottom right: The initial condition continuously parametrizes which sequence is activated. Bottom left: In addition, the RNN receives a two-dimensional task-irrelevant stochastic input that varies from trial to trial. **b**. Sequential activity during a single trial, neurons sorted by peak activity. **c**. Singular values of the activity matrix. The two dominant singular values come from the two-dimensional input. Nevertheless, as expected from sequential activity, the singular value spectrum has a long, slowly-decaying tail (participation ratio of 7.57). In comparison, the singular values of the partial reconstruction from the two trial slicing components (which capture the neural sequences, see panel d) retain the long tail, confirming that the two dominant modes in the data are input-driven. **d**. SliceTCA components for *R*_trial_ = *R*_neuron_ = 2, *R*_time_ = 0. Neurons are sorted according to their peak activity in the slice of the first component. **e**. Example of single-neuron activity in the raw data and the SliceTCA reconstruction. **f**. Comparison of the variance of the activity captured by sliceTCA, TCA, and PCA with or without intrinsic noise (mean variance plus/minus standard deviation over *n* = 10 simulations of the RNN). **g**. Cross-validated variance explained by fitting sliceTCA (*R*_trial_ = *R*_neuron_ = 2, *R*_time_ = 0) to the RNN activity while systematically varying the level of noise added at the level of the circuit dynamics (intrinsic noise, red), low-dimensional inputs (input noise, blue), or neural activity itself (observation noise, green).

While we were able to explore neural and temporal covariability in the feedforward model, it neglected trial covariability, which can capture temporally rich population dynamics such as neural sequences. Toward this end, we built a linear recurrent neural network (RNN) model to generate high-dimensional, condition-specific sequences^24^ while additionally integrating low-dimensional, condition-independent inputs (Methods and Extended Data Fig. 3a–c). As designed, the RNN activity could be decomposed into a few trial-slicing components corresponding to the embedded sequences and a few neural-slicing components corresponding to the inputs (Extended Data Fig. 3d). Through this model, we were able to systematically examine the effects of three different sources of noise: low-dimensional input noise, intrinsic noise in the circuit dynamics and observation noise (Methods). As expected, sliceTCA was able to achieve near-optimal denoising of observation noise (Extended Data Fig. 3g). Conversely, variability in the RNN activity coming from input noise was entirely retained by sliceTCA; this is because any variability in the inputs pushes the activity along a low-dimensional subspace determined by the input projection. Finally, sliceTCA performed well for intrinsic noise with a tendency to overfit for higher noise levels. These results clarify the robustness of sliceTCA to different sources and amounts of noise and provide insight into the relationship between the slice rank and neural circuit dynamics.

### Task-relevant information is distributed across slice types

**Fig. 2:**
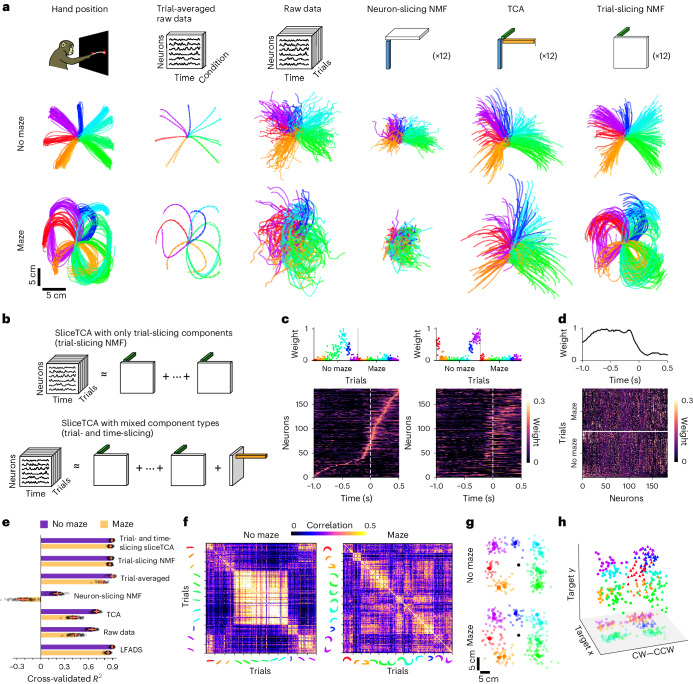
Time- and trial-slicing components identify preparatory and kinematic information in motor cortical activity, respectively. **a**, Behavioral and motor cortical trajectories (*n* = 182 neurons from M1 and PMd) during a classic center-out reaching task with straight reaches (top) and curved maze reaches (bottom; modified from ref. ^52^). Different colors indicate different reach directions. Hand position: hand positions during the experiment. Trial-averaged raw data: condition-wise trial-averaged reaches (dashed lines) versus neural population activity (solid lines), projected onto the two-dimensional (2D) subspace that best matches hand trajectories. Raw data: raw population activity mapped onto hand trajectories at single-trial resolution. Neuron-slicing NMF: denoised population activity mapped onto hand trajectories (neuron-slicing NMF, 12 components; equivalent to NMF performed on the trial-concatenated data matrix). TCA: denoised population activity (TCA, 12 components) mapped onto hand trajectories. Trial-slicing NMF: denoised population activity (trial-slicing NMF, 12 components) mapped onto hand trajectories. **b**, Schematic of a sliceTCA model with multiple components of the same slice type versus a model with mixed slice types. **c**, Two example trial-slicing components, with neurons ordered by peak activation times of the first component. Sequential patterns distinguish specific reach conditions (here, upper left versus upper right straight reaches). **d**, The single time-slicing component, which displays a high temporal weight preceding movement onset. Condition-specific neural weights are shown in the slice. **e**, *R*^2^ of fivefold cross-validated velocity decoding in each model (error bars represent the s.e.m. over *n* = 49 and *n* = 53 test trials for the maze and no-maze conditions, respectively, averaged over a fivefold cross-validation of 20 permutations of the trials). **f**, Correlations between neural weights on the time-slicing component in the PMd. Correlations were high for pairs of trials with similar reach direction and curvature and low for dissimilar reaches. **g**, Mapping of average activity in the time-slicing component before movement onset (from 0.75 to 0 s before onset) onto reach targets, revealing a strong association (*R*^2^ = 0.95 and *R*^2^ = 0.91, center-out versus curved reaches). **h**, Partially reconstructed activity from the time-slicing component, projected into a 3D subspace identified to maximally separate clockwise (CW) versus counterclockwise (CCW) movements and target *x* and *y* positions. Data points are clustered according to both reach direction and curvature, indicating that the time-slicing component encodes information about the dynamics of the upcoming movement (dots, clockwise reaches; triangles, counterclockwise reaches).

Based on the results of our toy model, we predicted that different slice types could capture different kinds of behaviorally relevant signals in neural data. We tested this hypothesis in a dataset comprising population recordings of the primary motor cortex (M1) and dorsal premotor cortex (PMd) during maze reaching and classic center-out (no-maze) reaching tasks (Fig. 2a, hand position). To quantify decoding performance, we linearly mapped population activity onto hand velocity (Methods). As a benchmark, we first mapped trial-averaged raw neural data onto kinematic trajectories, revealing a close match between behavior and neural activity (Fig. 2a, trial-averaged raw data). However, when we attempted to decode hand trajectories based on individual trials, we observed considerable trial-to-trial variability that corresponded poorly to kinematic data (Fig. 2a, raw data).

We reasoned that single-trial kinematic information might be present in the data but obscured by behaviorally irrelevant neural variability. If true, then the decoder should perform significantly better on properly denoised data. To test this, we first used a common approach of fitting a low-rank approximation using NMF (*R* = 12 components) to the *N* × (*TK*) matrix of trial-concatenated neural activity (‘neuron-unfolded’ data). Surprisingly, this decreased the performance of the decoder (Fig. 2a, neuron-slicing NMF), suggesting that the discarded variability contained information about hand kinematics. We wondered whether a better performance could be obtained with a method that explicitly identifies covariability across trials. Indeed, TCA-denoised data displayed a better match to the hand kinematics (*R* = 12 components; Fig. 2a, TCA). Yet, by constraining the decomposition to be low tensor rank (and thus also discarding temporal variability across neurons), TCA is unable to reconstruct neural sequences at a sufficiently high temporal resolution to allow for precise behavioral readout.

By performing TCA and NMF on the neuron-unfolded data tensor, we have assumed that behaviorally relevant information in the data is represented by neural covariability (Fig. 1d). However, previous work has emphasized that neural activity in motor regions is better described by stereotyped sequences that are distinct for each task condition^7,25^. Following this intuition, we performed the same decoding analysis on denoised trial-unfolded data, in which a *T* × (*NK*) matrix is approximated using NMF (*R* = 12 components). Remarkably, this simple change in the denoising strategy resulted in a significantly better match between trial-to-trial variability in the data and the hand kinematics (Fig. 2a, trial-slicing NMF). We further validated that the components obtained by trial-slicing NMF corresponded to reach-tuned sequences whose temporal orderings were reproducible across held-out data (Supplementary Fig. 2). These results reveal that, in this dataset, behaviorally relevant information was encoded by trial covariability (specifically, neural sequences) rather than by neural covariability.

Trial- and neuron-concatenated NMFs constitute two special cases of non-negative sliceTCA in which either neuron-slicing components or trial-slicing components exclusively are fitted. Therefore, we next asked whether we could identify additional information in the data by demixing different classes of covariability with sliceTCA. Previous work has identified preparatory signals in the PMd that indicate the dynamics of the upcoming movement^26^. Therefore, we hypothesized that we could capture preparatory signals in a time-slicing component with a stereotyped ramping profile and neural weights encoding reach targets and curvature on a trial-by-trial basis.

**Extended Data Fig. 4:**
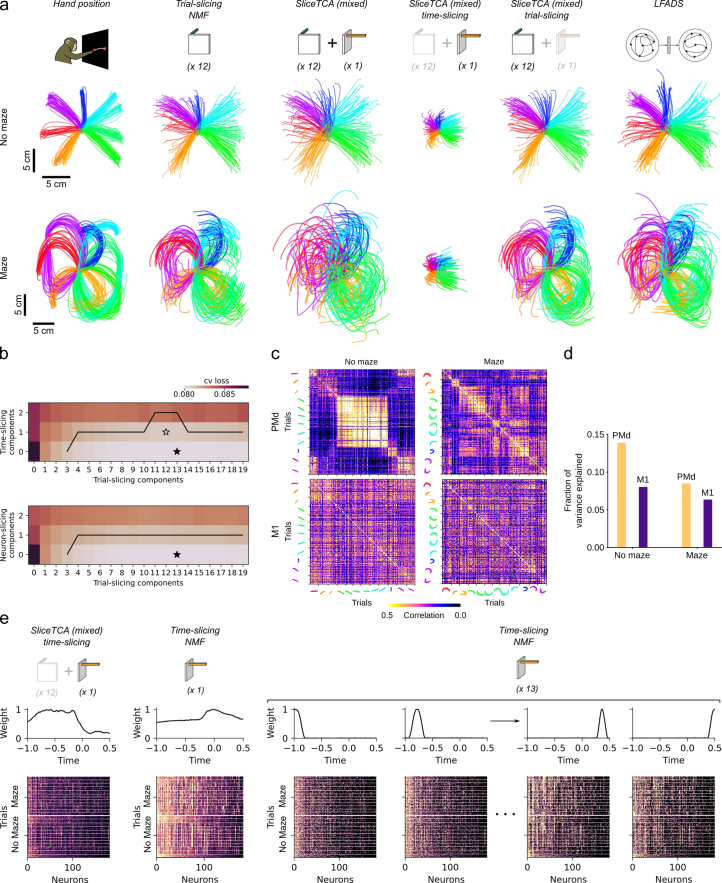
Analysis of the mixed covariability model used to decode reaching kinematics from motor cortical recordings (Dataset 1). **a**. Ordinary least squares decoding of the hand velocity from different decompositions. For comparison, hand position and the mapping from the reconstruction of the trial-slicing only NMF model (*R*_neuron_ = 0, *R*_time_ = 0, *R*_trial_ = 12) are reproduced from Fig. 2. Also shown are the decoding performances using the fit of the mixed covariability model (*R*_neuron_ = 0, *R*_time_ = 1, *R*_trial_ = 12), based on the full reconstruction of all components in the model, as well as the partial reconstruction of the data tensor from only the time-slicing component or only the trial-slicing components. Note that adding the single time component to the mixed model before decoding decreases performance, indicating that it captures variability in the neural data that is not directly relevant to movement. Finally, we show the decoding performance based on the reconstruction from autoLFADS (27, fitted on the cloud-based NeuroCAAS platform; https://neurocaas.org/analysis/20). **b**. Cross-validated model selection. Top: The cross-validated loss is displayed as a function of the number of time- and trial-slicing components (for 0 neuron-slicing components). Bottom: Cross-validated loss as a function of the number of neuron- and trial-slicing components (for 0 time-slicing components). For 10 random seeds, data tensors were split in train- and test-data 80% and 20%, respectively), following a blocked masking procedure (Methods). Full black star marks the optimal model, and hollow black star marks the selected model analyzed here and in Fig. 2. Black thresholds represents the 95% loss elbow. **c**. Reach condition-specific structure in motor preparatory information is observed in time-slicing weights of PMd, but not of M1. Top: Correlation matrix between neural encoding weights for pairs of trials in the time-slicing matrix, showing similarity of PMd neuron weights for trials with similar reach direction and curvature (left/right indicate no maze/maze conditions, cf. Fig. 2f). Bottom: In contrast, M1 neuron weights reveal a lack of structure in its correlation matrices. **d**. Average fraction of the variance of PMd and M1 neuron activity explained by the single time-slicing component, separated by maze and no maze conditions. **e**. Comparison of sliceTCA and time-slicing NMF. Left: SliceTCA time-slicing component (*R*_trial_ = 12, *R*_time_ = 1, *R*_neuron_ = 0), cf. components of NMF applied to the time-unfolded data tensor for *R*_time_ = 1 (middle) and *R*_time_ = 13 (right). Neither time-slicing NMF model appears to capture pre-movement preparatory activity.

Toward this end, we used sliceTCA to add a single time-slicing component to the previous model with 12 trial-slicing components (Fig. 2b and Supplementary Fig. 3; *R*_neuron_ = 0, *R*_trial_ = 12, *R*_time_ = 1 selected based on the elbow of the cross-validated loss (Extended Data Fig. 4b)). In both the trial-slicing NMF model and the mixed covariability sliceTCA model, the trial-slicing components identified sequential neural activations for similar reach conditions that seemed to be continuously tuned to target angles (Fig. 2c and Supplementary Fig. 3). Decoding from these trial-slicing components (in either the mixed or unmixed model) led to significantly better performance as compared to the neuron-slicing and TCA models (Fig. 2e, Extended Data Fig. 4a and Supplementary Fig. 4). Furthermore, despite being a (multi)linear method, sliceTCA had a decoding performance on par with that of LFADS (latent factor analysis via dynamical systems)^27^ for straight reaches and performed better for the maze condition (*P* = 1.907 × 10^−6^, two-sided Wilcoxon signed-rank test; Fig. 2e and Extended Data Fig. 4a). We additionally noted that the trial-slicing partial reconstruction from sliceTCA mapped onto hand kinematics slightly better in the mixed model than in the trial-slicing-only model (Fig. 2e; *P* = 1.907 × 10^−6^, two-sided Wilcoxon signed-rank test). Intriguingly, while the single time-slicing component mapped poorly onto hand kinematics (Extended Data Fig. 4a), its time course displayed a peak around 100 ms before movement onset followed by a drop in amplitude (Fig. 2d), consistent with a motor preparatory signal.

If the time-slicing component contains motor preparatory information, we would further expect it to contain information regarding the parameters of the upcoming movement^26^. Indeed, the neural encoding weights in the PMd (but not M1; Extended Data Fig. 4c,d) were correlated across similar conditions and encoded both reach direction and curvature (Fig. 2f–h). Therefore, while the trial-slicing components directly encoded motor sequences governing hand kinematics, the time-slicing component contained primarily preparatory information about movement parameters. Interestingly, simply applying NMF without explicitly demixing covariability classes was not able to recover this preparatory signal (Extended Data Fig. 4e and Supplementary Fig. 5). Together, these results show that behaviorally relevant information in neural data can be spread across different slice types, motivating the need to demix covariability with sliceTCA.

### Pipeline for sliceTCA model selection and optimization

**Fig. 3:**
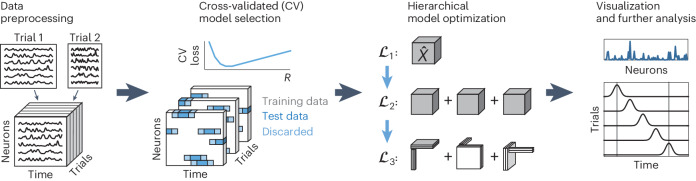
SliceTCA model selection, optimization and analysis pipeline. First, neural data are preprocessed to form a data tensor. In experiments with variable trial lengths, this could include temporal warping, exclusion of outlier trials and/or trimming to the time period of interest. Second, model selection is performed to choose the number of components of each slice type (*R*_neuron_, *R*_trial_, *R*_time_) based on the cross-validated mean-squared error (MSE) loss (blue curve). For cross-validation, we randomly assign blocks of consecutive time points (blue) within the data tensor as held-out data. The remaining entries of the tensor are used as training data (white). Specifically, the held-out entries are masked when computing the loss used to optimize the model parameters. To reduce temporal correlations between the training and testing data, we discard a brief period from the ends of the held-out blocks (light blue) from both training and testing. We use only the interiors of these blocks as test data (dark blue). Note that, because there are three slice types, the optimization is a 3D grid search on the cross-validated loss. Third, a hierarchical model optimization procedure is performed to identify a unique solution to the two mathematically identified invariance classes by optimizing the MSE loss ${{{{\mathcal{L}}}}}_{1}$ followed by secondary and tertiary losses ${{{{\mathcal{L}}}}}_{2}$ and ${{{{\mathcal{L}}}}}_{3}$ (Methods). After this procedure, the resulting loading vectors and slices can be analyzed.

**Extended Data Fig. 5:**
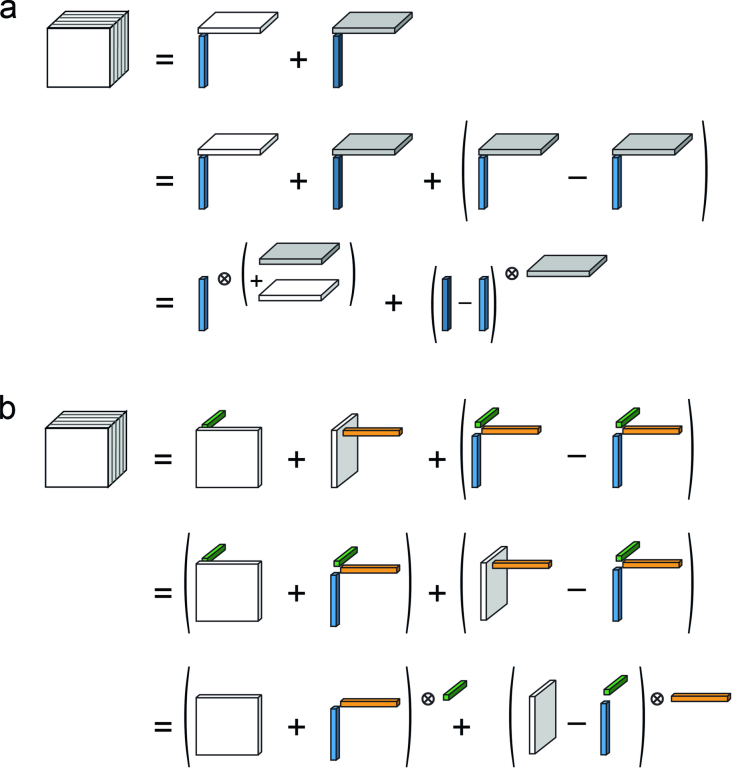
Schematic of the two sliceTCA invariant transformations. **a**. Example of a within-slice-type invariant transformation in a slice-rank-2 tensor formed by adding and subtracting a slice-rank-1 component with the same loading vector as component 1 and the same slice as component 2. These terms can be absorbed into the original two components, resulting in two equivalent decompositions. **b**. Example of a between-slice-type invariant transformation in a slice-rank-2 tensor. Here, a rank-1 tensor constructed by the two components’ loading vectors (green and yellow) and a third free vector (blue) is added and subtracted. These terms can be absorbed into the original two components, resulting in two equivalent decompositions.

**Extended Data Fig. 6:**
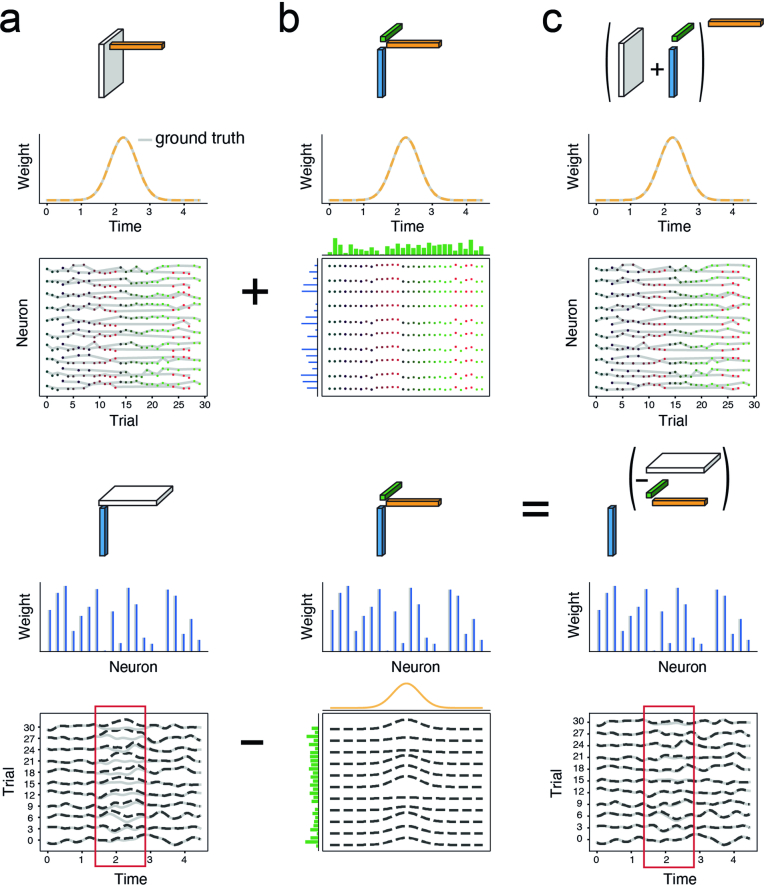
Between-slice-type invariance in the feedforward model. **a**. The activity of the feedforward model (Extended Data Figure 2) can be decomposed into the sum of a time-slicing component (top) and a neuron-slicing component (bottom). In this example, the Gaussian Process is not rectified and the data is not non-negative, leading to bleedthrough between the two components due to this invariance class (bottom, red rectangle shows discrepancy between model and ground truth). This invariance class was not observed in the original non-negative model (Fig. 1e–g, Extended Data Figure 2) because there are fewer permissible transformations when the factors are more constrained. **b**. Example of a rank-1 tensor that can be passed between the two components. Two of the loading vectors are identical to the loading vectors of the components in a. The third is unconstrained. **c**. By choosing the trial loading that minimizes a specified objective function, a unique solution can be found. Here the correlation between the activity of trial-neuron pairs was minimized in the neuron slicing component, resulting in a fit that matches the ground-truth values.

**Extended Data Fig. 7:**
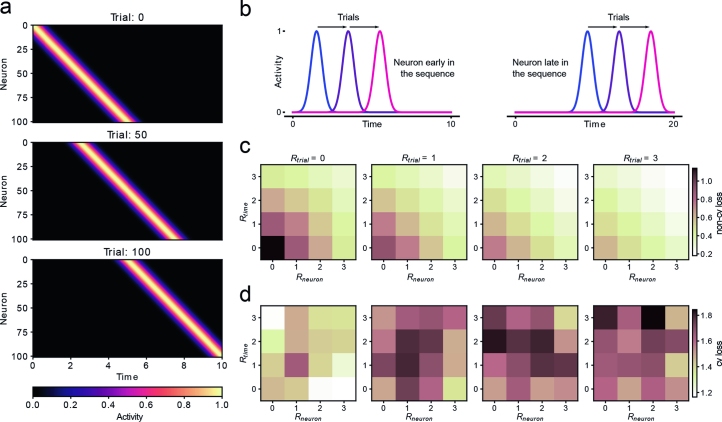
Drifting neural sequences have high slice rank. **a**. Over trials, the time point at which a neural sequence starts continuously drifts later and later within the trial. In the model, the activity at trial *i* ∈ {1, …, *K*} for neuron *j* *∈*{1, …, *N*} and time point *k* ∈ {1, …, *T*} is given by ${X}_{ijk}={{{\exp}}}\left({-{\left(\frac{i+j-k}{\sigma T}\right)}^{2}}\right)$. Here we used *T* = 2*N* = 2*K*, *N* = 100, *σ* = 0.1. **b**. Left: A neuron that activates early in the sequence begins to be activated later and later in time, for increasing trials. Right: similar drift for a neuron that fires late in the sequence. Therefore, in addition to there being a sequence of neural activation whose timing drifts from trial to trial (as in panel a), there is equivalently a sequence of activations over trials whose timing drifts from neuron to neuron. This symmetry of the sequences in the data tensor means that it can neither be captured by few neuron slicing components nor by few trial slicing components. **c**. SliceTCA gridsearch for the optimal number of components (fit on the full tensor, not cross-validated). The loss continues to decrease indefinitely as the number of components increases. **d**. Cross-validated sliceTCA gridsearch for the optimal number of components. Retaining blocks of trial x neuron x time creates a test set that is nearly completely decorrelated (in neuron, trial or time) from the train set; thus the test loss is close to random and larger in magnitude than in panel c due to overfitting.

**Extended Data Fig. 8:**
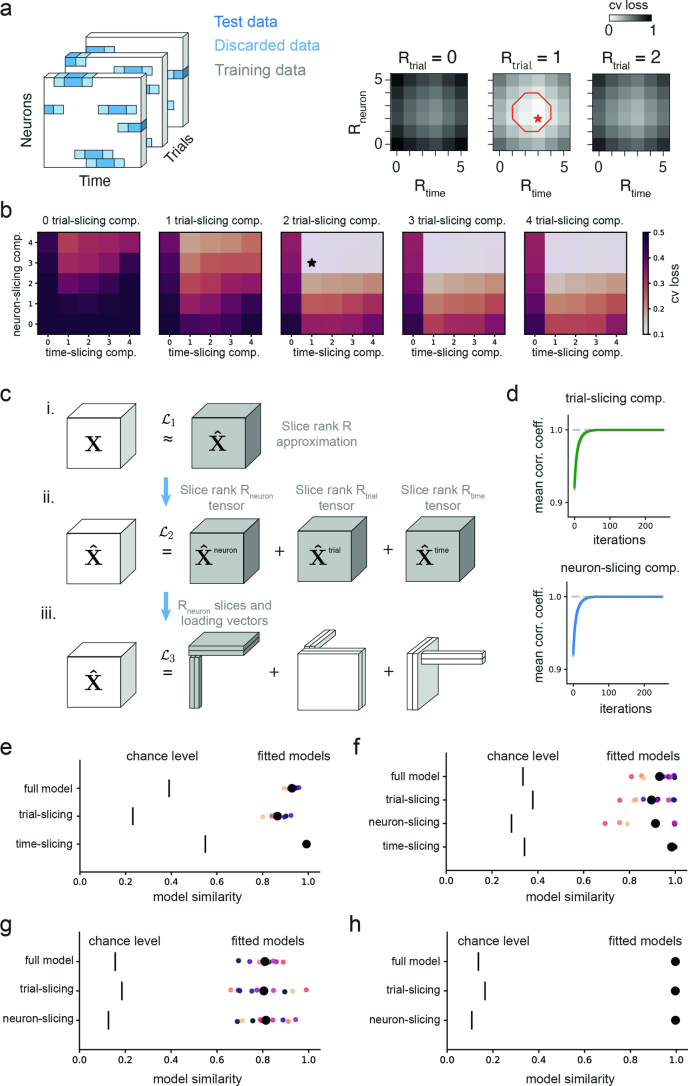
SliceTCA model selection and optimization. **a**. Cross-validated model selection procedure. We randomly assign blocks of consecutive time points (blue) within the data tensor as held out data. The remaining entries of the tensor are used as training data (white). To reduce correlations between the training and testing data, we discard a brief period from the ends of the held-out blocks (light blue) from both training and testing. We use only the interiors of these blocks as test data (dark blue). We run a 3D grid search on the cross-validated loss (right). We either choose the optimal model (red star) or a model at the ‘elbow’ of the loss function (red circle). **b**. Validation of the cross-validation procedure. For ten random seeds, data tensors were split in training data and held out data (80% and 20%, respectively), following a blocked masking procedure as described in Methods. Grid shows mean squared error loss for different numbers of components of each slice type, averaged across ten cross-validation folds. The ground truth model was a tensor formed by approximating the cortico-cerebellar dataset tensor using a decomposition of *R*_neuron_ = 3, *R*_time_ = 1, *R*_trial_ = 2 and subsequently adding independent Gaussian noise (*σ* = 0.1) to each tensor entry. The grid search recovers the correct number of components of each slice type (black star indicates optimal model). **c**. Hierarchical model optimization procedure. Schematic illustrates the three optimization stages to be performed in sequence (gray represents the entries which change at each stage). i. We first fit the model on all data (optimizing the MSE or ${\mathcal{L}}_{1}$ loss), resulting in the approximation ${\hat{X}}$ to the data tensor X. ii. Next, we optimize the auxiliary loss ${\mathcal{L}}_{2}$ (a function of the partial reconstructions ${\hat{X}}^{\rm{neuron}}$, etc.) in order to find a solution to the between-slice-type invariances. This can be considered as a regularization step that affects the relative weighting of each slice type. iii. Finally, the auxiliary loss function ${\mathcal{L}}_{3}$ (a function of the loading vectors and slices, for example, **u**^(*r*)^ and A^(*r*)^) is optimized to solve the factor rotation problem for each slice type separately (here, shown for the neuron slice type components). **d**. Results of running the tensor-passing (${\mathcal{L}}_{2}$) optimization step on the decomposition shown in Fig. 4, with 10 random seeds, so that the free vector of the rank-1 tensor was initialized differently each time. During optimization, the free vector is optimized to minimize the L2-norm of slices. Over 250 iterations, the optimization procedure lead to a unique solution, observable in a perfect correlation between slice weights both for trial- (green, top) and neuron-slicing components (blue, bottom). Each transparent line is the pairwise correlation between two solutions found based on 10 different initialization seeds. **e-h**. Model similarity scores for all three datasets. For a given number of components, we fitted ten sliceTCA models from different parameter initializations. We then compared the similarity between the model with the lowest mean squared error (MSE) and all other models using a metric originally proposed in 56, and adapted from 4. For this, we unfolded each component’s slice into a vector and computed two angles per component: one corresponding to the loading vectors of the two models, and another corresponding to the unfolded slices. Components were matched using the Hungarian algorithm (as in 4). We report a similarity score that is averaged over all components (‘full model’) as well as slice type-specific similarity scores. Single small dots are scores for models from different initializations, color-coded by MSE (brighter colors indicate higher MSE). Lines denote average chance level over 100 shuffle repetitions, for which the elements of vectors of one component were permuted before computing the similarity score. **e**. Similarity scores for reaching dataset (Dataset 1, shown in Fig. 2). See Supplementary Figure 6 to see components of the lowest-similarity model. **f**. Similarity scores for multi-region dataset during decision-making (Dataset 3, shown in Fig. 5). **g-h**. Similarity scores for cerebello-cortical imaging data (Dataset 2, shown in Fig. 4) before (**g**) and after (**h**) hierarchical optimization of ${\mathcal{L}}_{2}$ and ${\mathcal{L}}_{3}$ to identify a unique solution.

Dimensionality reduction methods, while powerful, can prove challenging in practice. First, robustly identifying the optimal number of components is a crucial yet challenging step in interpreting the dimensionality of neural representations^28,29^. Even after the rank is fixed, invariances in the decomposition may lead to multiple possible solutions (for example, matrix factorizations are known to be invariant to invertible linear transformations such as rotations), although adding a non-negativity constraint (as in the case of NMF) confers better uniqueness properties compared to unconstrained matrix factorizations^30^. Thanks to the tractability of sliceTCA, we were able to characterize its mathematical invariances (Extended Data Figs. 5 and 6). To provide objective criteria for model selection and uniqueness, we developed a full analysis pipeline for sliceTCA, including data preprocessing, model selection, model optimization and visualization (Fig. 3). First, trials must be time-warped, trimmed or masked for the data to be shaped into a tensor. Alignment to key events is an important consideration to remove additional sources of variability that are not incorporated into sliceTCA assumptions, such as sequences that are jittered or warped in time^31^ (Extended Data Fig. 7). We have taken the approach of piecewise linearly warping trials to task-relevant variables, but unsupervised warping is a promising alternative^32^. Second, to choose the optimal rank, we developed a rigorous cross-validation procedure to identify the number of components of each slice type, which we validated on ground-truth data (Extended Data Fig. 8a,b). Third, we identified the two invariance classes leading to equivalent sliceTCA decompositions (that is, for which different sets of weights of the components yield the same reconstructed tensor approximation) (Methods and Extended Data Figs. 5 and 6) and developed a hierarchical model optimization that adds additional constraints in the form of ‘sub-losses’ that must be minimized at three stages (Methods and Extended Data Fig. 8c,d). Model similarity analysis across different parameter initializations quantified the nonuniqueness of sliceTCA solutions (Extended Data Fig. 8e–h and Supplementary Fig. 6) and confirmed that the hierarchical optimization procedure leads to unique solutions. We further prove mathematically that a unique solution is guaranteed if each of the sub-losses is unique (Supplementary mathematical notes). Using a rigorous and standardized pipeline for model selection, fitting and optimization allows the user to make a robust, principled choice of sliceTCA decomposition for further interpretation.

### Denoising task-relevant manifolds

**Fig. 4:**
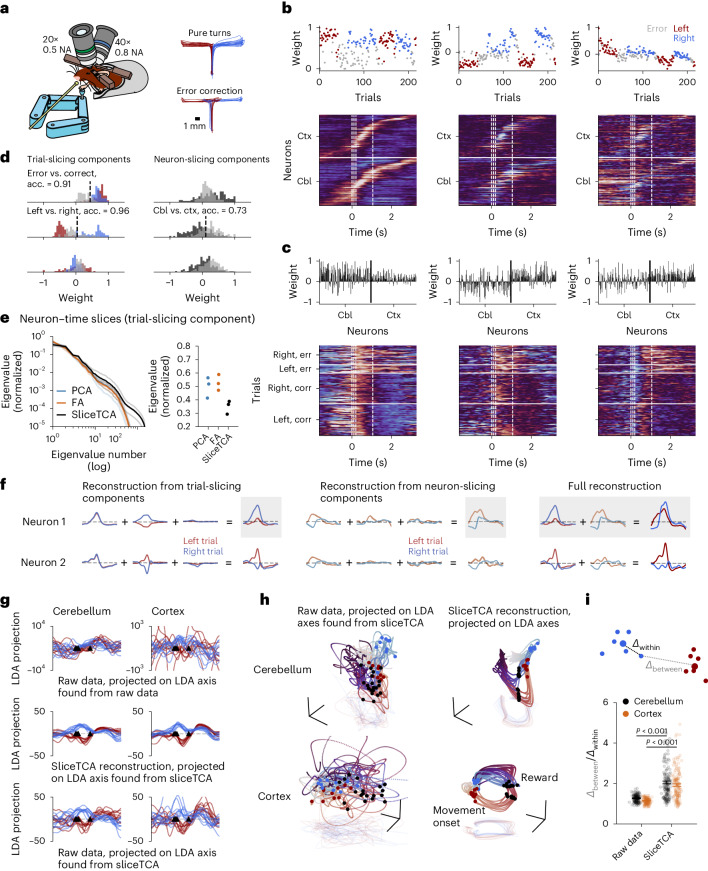
SliceTCA denoises task representations in simultaneously imaged cortical and cerebellar populations. **a**, Schematic of the experimental setup. Image modified from ref. ^18^. NA, numerical aperture. **b**, Trial-slicing components. Loading vector weights are colored according to trial type. In the slices, neurons are sorted within each region (Cbl, cerebellum; Ctx, premotor cortex) by the latency of the maximum activation in the first component. Dashed lines indicate movement onset, mid-turn, movement end and reward. **c**, Neuron-slicing components. In the slices, trials are separated by left/right and correct (corr)/error (err). Within blocks, trials are plotted in increasing order (ascending). **d**, Histograms of loading weights, colored by trial type and region. We classified weight vectors (correct versus incorrect, left versus right correct trials, cerebellum versus cortex). acc., accuracy. **e**, Left: eigenspectra of the covariance matrices of the slices of the trial-slicing components identified by PCA, factor analysis (FA) or sliceTCA, averaged over components (thick lines; transparent lines indicate individual components). Right: leading eigenvalue for each component. **f**, Single-neuron reconstructions of low-slice-rank approximations. The full sliceTCA reconstruction (right) is obtained by summing the contributions of all components from both slice types. **g**, Data from ten example trials per condition, projected onto an axis that maximally separates left and right correct trials between movement onset and reward. LDA, linear discriminant analysis. **h**, Neural manifolds in an orthonormalized neural subspace found with LDA (axis 1, same as **g**; axis 2 separates movement onset versus reward; axis 3 separates reward expectation versus post-reward) from raw data and sliceTCA reconstruction. **i**, Separation of left versus right trajectories from full data and data denoised with sliceTCA. *Δ*_within_ (*Δ*_between_) indicates the distance of the population vector around the time of movement onset to the center of the cluster of data points in its same (the opposite) trial class. Each dot represents a different trial for *n* = 151 correct left and right trials. Error bars represent the bootstrapped 95% confidence intervals of the mean. Left and right trajectories are more separable after sliceTCA denoising (two-sided Wilcoxon signed-rank test, *P* < 0.001 for both the cerebellum and premotor cortex).

**Extended Data Fig. 9:**
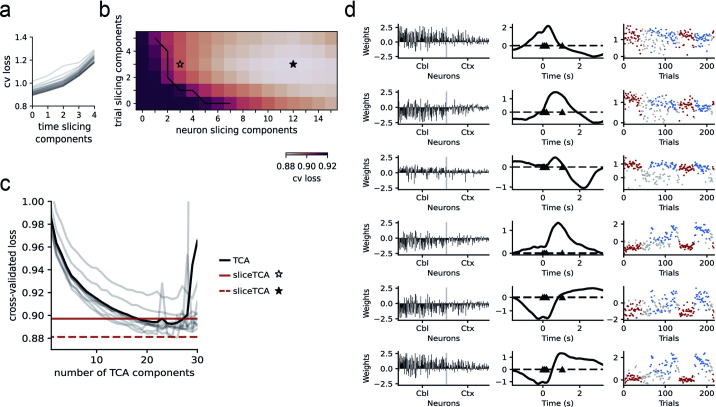
Cross-validated model selection and comparison to TCA for cerebello-cortical imaging data (Dataset 2). **a**. The cross-validated loss as a function of the number of time-slicing components. Transparent grey lines show different combinations of 0-5 neuron- and 0-5 trial-slicing components. This indicates that adding any time-slicing components leads to an overfitting of the data. **b**. The cross-validated loss as a function of the number of neuron- and trial-slicing components (for 0 time-slicing components). The filled star indicates the minimum loss (that is, the optimal model), while the black line indicates the 80% loss elbow. The hollow star indicates the model selected for further analysis in Fig. 4. **c**. TCA applied to the cerebello-cortical dataset. Cross-validated loss as a function of the number of TCA components (grey curves indicate ten different cross-validation folds, with the mean shown in black). The solid red line shows the performance of the sliceTCA model shown in Fig. 4 (that is, at the elbow of the sliceTCA loss), while the dashed red line shows the optimal sliceTCA model (components shown in Supplementary Figure 7, dashed red line). **d**. Factors of the 6-component TCA model. Note that the first three and last three components show redundancy in the trial factors, while the corresponding time factors cover different periods of the task.

With a standardized data analysis pipeline established, we next applied unconstrained sliceTCA to a new dataset consisting of the *z*-scored fluorescence traces of simultaneously imaged granule cells in the cerebellum and pyramidal neurons in the premotor cortex of mice performing a motor task (Fig. 4a)^18^. Using the sliceTCA analysis pipeline, we selected a model with three trial-slicing components and three neuron-slicing components at the elbow of the cross-validated loss function (Fig. 4b,c and Extended Data Fig. 9a; similar components were observed in the optimal model (Supplementary Fig. 7)). By comparison, TCA required 18 components to attain the same performance and displayed redundancy in the fitted components (Extended Data Fig. 9c,d). The cross-validation procedure resulted in no time-slicing components, as they consistently led to an increased test loss for this dataset (Extended Data Fig. 9a). The first trial-slicing component captured temporally distributed cerebellar and cortical time courses that were common to both left and right correct reaches but distinct from error reaches (Fig. 4b,d). In contrast, the second trial-slicing component accounted for the differential activation in left versus right trials (Fig. 4b,d). A third component decayed slowly over trials, possibly representing adaptation over the course of the session (Fig. 4b).

In addition, the three neuron-slicing components captured trial-specific activations of population modes localized around the time of movement or reward (dashed lines, Fig. 4c), with prolonged (and enhanced) activity in error trials, compared to correct trials, in the first and third components (two-sided Mann–Whitney *U* test, *P* < 0.001 for both components). Interestingly, the second neuron-slicing component captured differences between cerebellar and cortical activity (Fig. 4c,d). We next asked how sliceTCA compares to matrix factorization methods that do not demix neural and trial covariability. To test this, we performed PCA and factor analysis on the neuron and trial unfoldings of the data tensor (Fig. 4e and Supplementary Fig. 8). Demixing covariability classes with sliceTCA resulted in components with higher-dimensional structure in the slices (Fig. 4e and Supplementary Fig. 8c,d). This suggests that simply applying PCA to the tensor unfoldings cannot capture as much variability in the data (for example, in timing for individual neurons or trials) because it may be obscured by other dominant covariability types. Together, these results show that sliceTCA identifies both task-specific (left, right, error trials) and region-specific (cerebellum versus cortex) variables by capturing the structure of neural data across multiple covariability classes.

We next examined how applying sliceTCA affects reconstructed neural activity (Fig. 4f). Toward this end, we compared the neural representations of the raw data in neural space to the reconstructed data from the sliceTCA model. The sliceTCA reconstruction captured the same top principal components as the raw data, confirming that it faithfully captured its overall structure (Supplementary Fig. 9). The advantage of including both neural covariability and trial covariability was reflected in the increased behavioral interpretability of the neural representations. For this, we projected the data onto the dimension that best separated left versus right correct trials during the period between movement and reward. The axis found from the sliceTCA reconstruction revealed more interpretable, denoised representations as compared to the dimension found from raw data (Fig. 4g). Similarly, the task-relevant neural manifolds, found by projecting neural trajectories onto a subspace that separates activity along three task-relevant dimensions (Methods), appeared substantially denoised when sliceTCA was applied compared to a direct projection of the raw data (Fig. 4h and Supplementary Fig. 9). We confirmed this denoising effect by measuring the distance between left and right trials around the time of movement onset in sliceTCA reconstructions as compared to the raw data (Fig. 4i). Our results indicate that sliceTCA, by grouping behaviorally similar trajectories in an unsupervised manner, increases the distance between trajectories of behaviorally distinct trials. Together, these findings show that sliceTCA is able to denoise task-relevant representations in neural data in an unsupervised fashion.

### Identifying region-specific covariability patterns

Thus far, we have shown that mixed covariability occurs within the same neural population. However, the need to consider multiple covariability classes becomes even more crucial in multiregion recordings, as different brain areas are better described by different classes^7^. Yet, relying on different data tensor unfoldings for each region would require that they be analyzed separately without leveraging the simultaneous nature of such data. Therefore, we asked whether sliceTCA could demix area-specific representations in distinct slice types.

**Fig. 5:**
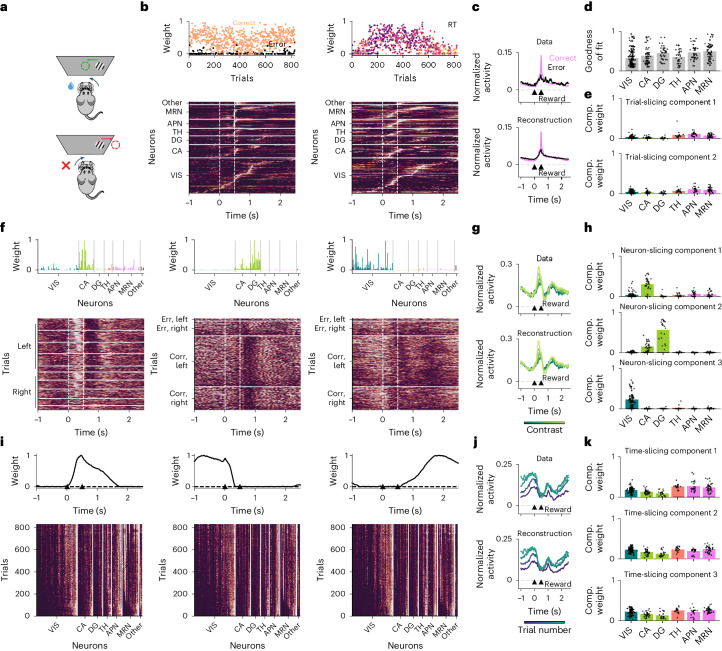
SliceTCA identifies region-specific sensory and behavioral variables in multiregion recordings. **a**, Schematic of the perceptual decision-making task from the IBL. Image modified from ref. ^53^. Green (red) arrows and circles indicate correct (incorrect) actions. **b**, Trial-slicing components: the loading vector of component 1 shows a separation between correct (orange) and error (black) trials. In component 2, the color scale in the loading vector indicates the log reaction time (RT). In the corresponding slices: VIS, visual cortex; CA, hippocampus; DG, dentate gyrus; TH, thalamus; APN, anterior pretectal nucleus; MRN, midbrain reticular nucleus. White lines indicate stimulus onset and reward or timeout onset. Slice weights are normalized to [0, 1] for each neuron separately and sorted by the latency of the peak activation within each region (separately for each component). **c**, Top: PSTH of an example neuron from the anterior pretectal nucleus showing reward-locked activation for correct/error trials (pink/black). Bottom: PSTH built from the full sliceTCA reconstruction. Arrowheads indicate stimulus onset and reward. **d**, Reconstruction performance (Methods) of the full sliceTCA model, separated by region. Black dots indicate individual neurons. **e**, Contribution of each trial-slicing component to the overall reconstruction. Comp. weight, component weight. **f**, Neuron-slicing components: trials are grouped into blocks separately for different components. In component 1 (hippocampal region CA1 related), trials are grouped by contrast separately for left/right trials (within left/right, contrast increases from bottom to top). In components 2 (dentate gyrus related) and 3 (visual cortex related), trials are grouped into blocks by left/right and correct/error. For all slices, within each block, trials are sorted in increasing order (ascending). Each slice is normalized to [0, 1]. **g**, Top: PSTH of an example hippocampal neuron for low to high contrast (dark to light green). Bottom: PSTH built from the full sliceTCA reconstruction. **h**, Contribution of each neuron-slicing component to the overall reconstruction. **i**, Time-slicing components: in the slices, neurons are sorted within each region according to increasing activation in early trials after normalizing weights for each neuron to [0, 1] (same sorting across components). **j**, Top: PSTH of an example visual cortical neuron for early to late trials (indigo to teal). Bottom, PSTH built from the full sliceTCA reconstruction. **k**, Contribution of each time-slicing component to the overall reconstruction.

**Extended Data Fig. 10:**
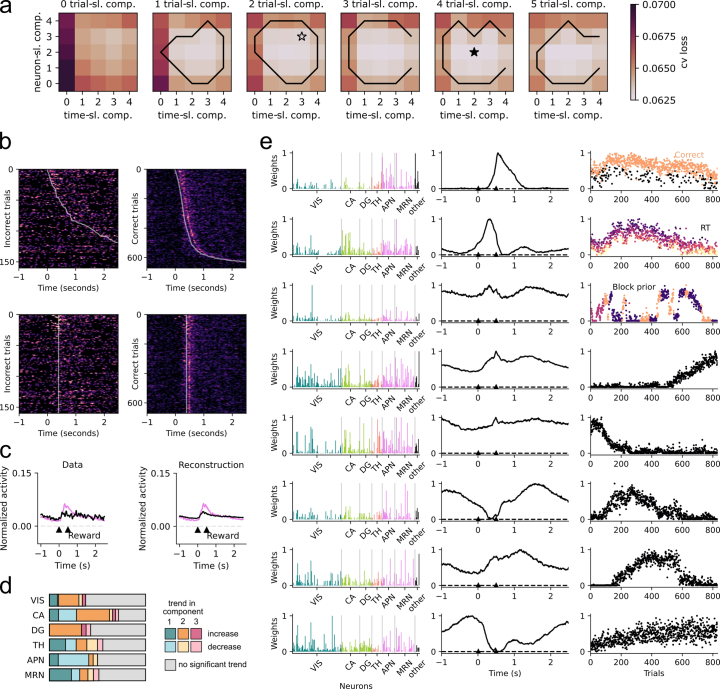
Cross-validated model selection, effects of temporal warping, and comparison to TCA for multi-region recordings during decision making (Dataset 3). **a**. Cross-validated model selection. For 10 random seeds, data tensors were split in train- and test-data (80% and 20%, respectively), following a blocked masking procedure as described in Methods. Full black star marks the optimal model, and hollow black star marks the selected model analyzed in Fig. 5. Black thresholds describe a 95% loss elbow. **b**. Effect of piecewise linear warping on the example APN neuron shown in Fig. 5c. Trials are ordered by the time of reward (white line; for visibility of neural responses, line has been shifted left by 100 ms). **c**. Single-neuron response of the same ARN neuron in correct vs. incorrect trials without time-warping (c.f. Fig. 5c). **d**. To determine whether the three time-slicing components found by sliceTCA reconstructed an increasing baseline for most neurons or whether responses in single task periods changed more strongly than others, we compared the slope of linear trends over trials (for each neuron separately) across the three time-slicing components. Specifically, we fitted linear models with a multiplicative term for each neuron: *w*_*k*,*r*_ = *β*_0_ + *β*_1_*k* + *β*_2_*δ*_*r*=2_ + *β*_3_*δ*_*r*=3_ + *β*_4_*kδ*_*r*=2_ + *β*_5_*kδ*_*r*=3_ + *ε*_*k*,*r*_ where *w*_*k*,*r*_ is the weight in trial *k* for component *r*, and *δ*_*r*=2_ = 1 if the weight belongs to component 2, *δ*_*r*=2_ = 0 otherwise (same for *δ*_*r*=3_, whereas component 1 is the reference class). We performed analyses of variance for each neuron to test whether multiplicative terms explained a significant part of variance (at a Bonferroni-corrected significance level *α* = 0.05 with *n* = 221 neurons). We discarded neurons with non-significant multiplicative terms (grey). For neurons with significant differences in the rate of change across time-slicing, we compared rates of change between components. Each neuron was classified by the highest absolute change coefficient and its sign, leading to six classes for three time-slicing components. **e**. 8-component TCA model on multi-region Neuropixel dataset. Trial loading vectors are color-coded by the behavioral or task variable most correlated with the weights (if any). Similarly to sliceTCA, component 1 was correlated with trial outcome and component 2 with the reaction time. However, components 3-8 tile the sequence of trials but have little correlation with trial outcome, reaction time or the block switching structure of the IBL task.

To test this idea, we took advantage of a recently published dataset consisting of Neuropixels recordings across six brain regions during a perceptual decision-making task (Fig. 5a)^19^. Our cross-validation procedure selected a model with eight components: two trial-slicing components, three neuron-slicing components and three time-slicing components (Extended Data Fig. 10a and Supplementary Fig. 10). The two trial-slicing components identified variables related to behavioral performance (Fig. 5b). The first trial-slicing component separated correct from incorrect trials (two-sided Mann–Whitney *U* test, *P* < 0.001), and the corresponding slice was characterized by reward-locked temporal response profiles in midbrain nuclei (anterior pretectal nucleus and midbrain reticular nucleus), which we validated in single-neuron peristimulus time histograms (PSTHs) (Fig. 5c) and in nonwarped data (Extended Data Fig. 10b,c). The second trial-slicing component instead featured temporally heterogeneous responses in all regions and correlated inversely with the log reaction times (Pearson’s *r* = −0.35, *P* < 0.001, *n* = 831 trials; Fig. 5b). We next asked how these components contributed to the activity of different regions. The full sliceTCA reconstruction explained 33–49% of neural activity, depending on the region (Fig. 5d). Of this reconstructed activity, the two trial-slicing components contributed considerably to neurons in the anterior pretectal nucleus, midbrain reticular nucleus and thalamus (19 ± 10%, mean ± s.d., *n* = 75 neurons; Fig. 5e). Thus, the trial-slicing components identified stereotyped activations in subcortical regions (thalamus, anterior pretectal nucleus and midbrain reticular nucleus) that were linked to behavioral performance across trials.

In contrast, the three neuron-slicing components identified three distinct clusters of neurons corresponding to cortical regions: the hippocampus, dentate gyrus and visual cortex (Fig. 5f). These components, therefore, represented population-wide covariability patterns that were specific to each of these regions. The slice of the hippocampus-preferring component was characterized by a contrast-dependent activation between the sensory cue and reward (correlation of stimulus-evoked responses with contrast, Pearson’s *r* = 0.40, *P* < 0.001; Fig. 5f,g), a feature that was less prominent in the dentate gyrus and not observed in visual cortex-preferring components (*r* = 0.11, *P* = 0.002 for dentate gyrus, *r* = −0.05, *P* = 0.14 for visual cortex). In the dentate gyrus-preferring component, we observed post-reward suppression on correct (rewarded) trials, which was significantly shorter on error trials (two-sided Mann–Whitney *U* test, *P* < 0.001; Fig. 5f). The final visual cortex-preferring component revealed pre-stimulus activation that increased in strength over trials (Pearson’s *r* = 0.55, *P* < 0.001; Fig. 5f), possibly indicating the emergence of a predictive signal of cue onset over the course of the experiment. Each component contributed to a large fraction of the sliceTCA reconstruction in its respective region (37 ± 21%, *n* = 138 neurons; Fig. 5h). Therefore, the three neuron-slicing components represented different task-relevant features that were separately encoded in hippocampal, dentate gyrus and visual cortical population responses.

Finally, the remaining time-slicing components partitioned the task duration into three distinct periods: early (pre-stimulus and stimulus onset), late (post-reward) and reward periods (Fig. 5i). The corresponding slices revealed smooth variations of the strength of each of these components in single neurons over the course of the experiment. While these changes appeared low-rank, simply replacing them with a TCA component led to a drop in the reconstruction error (Supplementary Fig. 12). Furthermore, given the strong similarity of the three slices, we asked whether the components could sum to a flat trial-varying baseline for each neuron. However, we observed examples of a broad range of modulation patterns of PSTHs, with slowly varying activity that changed heterogeneously over trials for the three task periods (for example, in Fig. 5j). Indeed, a substantial proportion of neurons across all regions showed significantly different rates of change in trial weights across the three components (analysis of variance, *P* < 0.05 with Bonferroni correction, *n* = 221 neurons; Extended Data Fig. 10d). Moreover, these three components contributed substantially to the sliceTCA reconstruction across all recorded regions (62 ± 18%, *n* = 213 neurons; Fig. 5k), demonstrating that the dataset was dominated by time covariability. Therefore, we asked whether the task-relevant and region-specific information observed in the trial and neural slice-type components would be visible without explicitly demixing the covariability classes with sliceTCA. However, simply applying NMF to the relevant unfoldings led to neural loadings that were not clustered by region and trial loadings that were not correlated with behavior (Supplementary Fig. 13). In contrast to sliceTCA, TCA components were less region-specific (Extended Data Fig. 10e). Together, these results show that, by accounting for different classes of covariability, sliceTCA is able to demix multiregion recording data into brain-wide representations of task period, behaviorally relevant stereotyped activity and population-wide patterns of covariability encoded by individual regions.

### Geometric interpretation of mixed covariability

Dimensionality reduction methods such as PCA allow for the interpretation of neural representations as trajectories embedded in a low-dimensional latent subspace within the full neural activity space. In sliceTCA, the neuron-slicing components can be interpreted in the same way owing to their relationship to standard matrix factorizations. However, the time- and trial-slicing components have different interpretations, as their loading vectors form bases of subspaces of the time and trial spaces. How, then, can we grasp the time- and trial-slicing components’ contributions to latent representations in neural activity space?

**Fig. 6:**
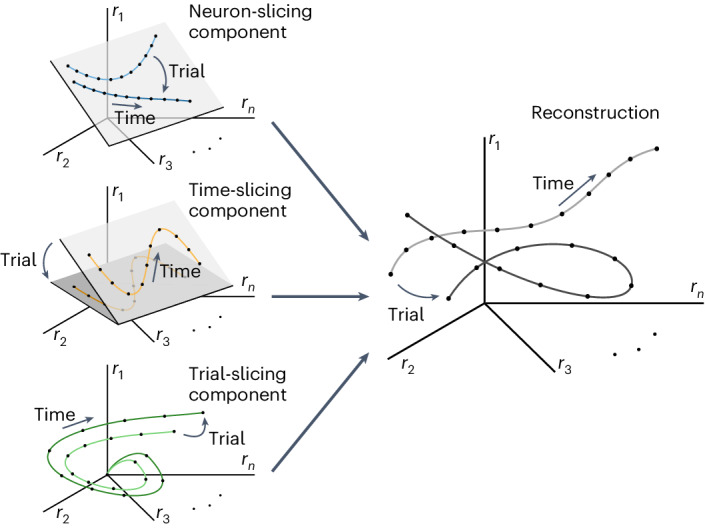
Different slice types capture latent variables with distinct geometric properties. Neuron-slicing component: example of two neuron-slicing components visualized in neural activity space. The latent activity is embedded in a 2D subspace, but their trajectories within that subspace are unconstrained. Time-slicing component: example of two time-slicing components. These are also embedded within a 2D subspace, but that subspace may vary over trials. Within each latent subspace, the latent trajectories have similar shape as they are constrained to linear combinations of a few characteristic temporal weights. Trial-slicing component: the trial-slicing components are not constrained to any subspace, as the neural encodings may change at every time point. These components describe linear combinations of a few potentially high-dimensional latent trajectories such as neural sequences. Note that, here, only one component is shown for clarity. In this case, the latent trajectory is simply re-scaled on each trial. Reconstruction: after summing these components, the full latent trajectories are not necessarily limited by any of the geometric constraints that characterize individual slice types.

We can answer this question by considering the contribution from each slice type separately. First, note that while the neuron-slicing components are constrained to an *R*_neuron_-dimensional subspace, their trajectories within that subspace are unconstrained over trials (Fig. 6, neuron-slicing component). Conversely, the trajectories of the *R*_time_ time-slicing components are constrained to be a linear combination of a few common temporal profiles, but the neural weight vectors can instead vary from trial to trial. Geometrically, this means that the reconstruction from these components lies within an *R*_time_-dimensional subspace that can now vary on each trial, but the embedded low-dimensional trajectories will have similar shapes (Fig. 6, time-slicing component). Finally, the *R*_trial_ trial-slicing components’ neural weights change at every time point, whereas trial weights are fixed. This corresponds to trajectories that are no longer embedded in a low-dimensional subspace but that are instead constrained to be linear combinations of stereotyped, potentially high-dimensional trajectories (Fig. 6, trial-slicing component; see Supplementary Fig. 14 for an example with multiple components). In this way, the three covariability classes that we have described can also be seen as three classes of latent activity in neural state space. All three classes combine to form the full reconstruction, which may appear more complex than any one component type (Fig. 6, reconstruction).

This geometric view illustrates that, by fitting different covariability classes, sliceTCA is able to capture latent trajectories that are no longer confined to a linear subspace despite still being a multilinear method. In contrast, traditional matrix factorization methods that capture only a single covariability class are restricted to one of the three geometric classes of latent activity in neural state space shown in Fig. 6, whereas TCA constrains its components to obey the geometrical constraints of all three classes simultaneously (Supplementary Fig. 15). In sum, sliceTCA is able to capture a broader range of covariability structure in neural data (and a broader range of latent representations in neural space) than related methods, all while remaining easily interpretable.

## Discussion

Neural activity is often interpreted as low-dimensional population modes representing patterns of covariation across neurons. We have advocated for an expansion of this view to describe three distinct classes of covariability: across neurons, across time and across trials. We further introduced sliceTCA, a new tensor decomposition that demixes these covariability classes in large-scale neural data. Through several example datasets, we demonstrated that sliceTCA captures more task-relevant covariability in fewer components, allowing for the discovery of intricate latent structure in population activity. Thus, sliceTCA expands the classic view of neural representations toward latent variables that are not constrained to a fixed low-dimensional subspace.

Our framework of multiple covariability classes addresses key limitations of the classic view on latent variables, which is unable to identify several types of structure commonly found in neural data (for example, neural sequences)^7,25^. Indeed, task-relevant sequences are a widespread phenomenon observed across brain regions^11,33,34^. While we emphasized the ability of trial covariability to capture condition-specific neural sequences, we note that this class can capture more complex forms of stereotyped temporal patterning across neurons^35–37^. In contrast, population modes characterized by variable timing on different trials (for example, in temporal difference learning^38^) are captured by neural covariability. Lastly, temporal covariability captures stereotyped trajectories embedded in reaching direction-specific subspaces within the neural state space^39^. We speculate that temporal covariability could also capture latent subspaces that evolve slowly due to learning or drift^12,14^. Our results support previous work arguing that different brain regions are better described by different covariability classes^7^. Importantly, we further show that, without demixing covariability, task-relevant variability can be obscured by components of the dominant slice type (Supplementary Figs. 5, 8 and 13). Therefore, demixing covariability classes may be a crucial step when considering multiregion recordings that may contain qualitatively distinct computations in different populations.

A long-standing challenge in systems neuroscience is the difficulty of mapping neural variability to changes in behavior^40^. Despite being unsupervised, sliceTCA was able to disentangle behavioral and task information in each of the datasets presented. This may be due to two factors: first, demixing covariability effectively ‘denoises’ components representing task variables that would have otherwise been occluded by other covariability classes. Second, trial-slicing components identify changes that are common across trials, which tend to be defined by task variables or behavioral outcomes. Indeed, we found that trial-slicing components often correlated with behavioral variables. Moreover, using feedforward and recurrent circuit models, we demonstrated how sliceTCA could offer a window into the computational roles of variables modeled by different slice types. Hence, we argue that the classical view on latent neural representations, which assumes that behaviorally relevant neural variability is correlated across neurons, is overly reductionist and may miss many types of neural dynamics underlying behavior.

A key advantage of matrix and tensor decompositions is their simplicity. (Multi)linear methods can perform as well as nonlinear methods in specific applications while remaining considerably more interpretable (Extended Data Fig. 4a). Indeed, the analytical tractability of sliceTCA enabled us to characterize its invariance classes and to propose a method to identify a unique solution in the unconstrained case (Extended Data Fig. 8c). Identifying invariances is crucial for reproducibility and interpretation, as nonunique solutions may prohibit clear comparisons across datasets^41,42^. This issue is ever more important with the trend toward comparisons of neural data to task-trained neural networks, whose representations are known to be sensitive to model specifications^43,44^. Going forward, matrix and tensor decompositions could prove useful for comparing latent representations by virtue of their tractability.

SliceTCA is closely related to both TCA and PCA while offering more flexibility by capturing multiple covariability classes. However, in some cases, TCA or PCA may be sufficient to capture the data while also offering other advantages. If no demixing is required, PCA has the benefit of having a closed-form solution (but we note that PCA is a special case of sliceTCA that could be identified through our pipeline). In contrast, the stronger constraints imposed by TCA mean that it generally requires more components than PCA or sliceTCA yet has fewer parameters per component. As such, TCA and sliceTCA represent two sides of a trade-off between model parsimony and expressivity, which could be balanced by combining their respective strengths (Supplementary Fig. 12). Future work could generalize sliceTCA and TCA using the partition rank^45^. However, careful consideration would be required to fully understand the implications of a low-partition-rank decomposition, including potentially new invariances.

While tensor decompositions can be viewed as generalizations of matrix factorizations, they have specific limitations (for example, they are generally more computationally expensive^46,47^). Data tensors also require trimming, masking or warping trials to the same length. These preprocessing steps make implicit assumptions about the temporal structure of latent variables: warping assumes that latent variables are simply rescaled in time on different trials^48^, whereas trimming is more suitable when latent variables have a fixed intrinsic temporal structure independent of trial length (for example, background oscillations)^32^. Because of these considerations, we note that sliceTCA may not be a good fit for datasets in which these two kinds of temporal structure are mixed, datasets that lack a systematic trial structure or datasets in which activity is dominated by chaotic dynamics rather than patterns of covariation. More generally, time warping is a thorny issue for tensor decomposition when key events for alignment are unknown (Supplementary Fig. 11). Toward this end, unsupervised time-warping methods could help identify unlabeled events in the data, whether as a preprocessing step^32^ or performed simultaneously with dimensionality reduction^31,49^.

Together, tensor decompositions are useful for neural data, as they allow for the discovery of patterns in trial-structured data. While we focused on third-order tensors, data tensors of even higher order could be imagined by adding legs corresponding to days, conditions or even individuals^50,51^. Going forward, our framework of mixed covariability could, therefore, help advance our understanding of behaviorally relevant latent structure in high-dimensional neural data across brain regions and subjects.

## Methods

No original data were collected for this study. We analyzed data from three previous datasets^17–19^. All experiments were approved by the relevant bodies: the Institutional Animal Care and Use Committee of Stanford University (dataset 1), the Administrative Panel on Laboratory Animal Care and Administrative Panel on Biosafety of Stanford University (dataset 2), and the Institutional Animal Care and Use Committees of Cold Spring Harbor Laboratory (dataset 3). Additional experimental details can be found below.

### Definition of the sliceTCA model

#### Matrix rank and matrix factorization

Consider a data matrix consisting of *N* neurons recorded over *T* samples (time points): ${{{{X}}}}\in {{\mathbb{R}}}^{N\times T}$. Matrix factorization methods find a low-rank approximation $\hat{{{{{X}}}}}$ following equation (1), in which each component is a rank-1 matrix: *X*^(*r*)^ = **u**^(*r*)^ ⊗ **v**^(*r*)^, where ${{{{\bf{u}}}}}^{(r)}\in {{\mathbb{R}}}^{N}$ and ${{{{\bf{v}}}}}^{(r)}\in {{\mathbb{R}}}^{T}$ are vectors representing the neural and temporal coefficients, which are chosen to minimize a loss function. In other words, the activity of neuron *n* at time *t* is given by

2 $${\hat{X}}_{n,t}=\mathop{\sum }\limits_{r=1}^{R}{u}_{n}^{(r)}{v}_{t}^{(r)}$$

A common choice of loss function is the MSE:

3 $${{{\mathcal{L}}}}=\frac{1}{NT}{\left\Vert {{{{X}}}}-\hat{{{{{X}}}}}\right\Vert }_{F}^{2}$$

Constraints may be added to the minimization of the loss, such as non-negativity of the coefficients in NMF.

#### Slice rank and sliceTCA

A *d*-tensor is a generalization of a matrix to *d* legs (that is, a data matrix is a 2-tensor). Here, we are specifically concerned with 3-tensors typically used in neuroscience, in which the three legs represent neurons, time and trial/condition: ${{{{X}}}}\in {{\mathbb{R}}}^{N\times T\times K}$. SliceTCA extends the matrix factorization in equation (1) by fitting *X* with a low ‘slice rank’ approximation^23^. A slice-rank-1 *d*-tensor is an outer product of a vector and a (*d* − 1)-tensor. For the 3-tensors that we have been considering, this corresponds to the outer product of a ‘loading’ vector and a 2-tensor, thus making this 2-tensor a ‘slice’ of this slice-rank-1 tensor up to a scalar multiple determined by the loading vector.

Each sliceTCA component can be one of three different slice types. For example, a neuron-slicing component can be written as *X*^(*r*)^ = **u**^(*r*)^ ⊗ *A*^(*r*)^, where ${{{{{A}}}}}^{(r)}\in {{\mathbb{R}}}^{T\times K}$ is the time-by-trial slice representing the weights of the component across both time and trials and the vector **u**^(*r*)^ represents the neural loading vector. Components of other slice types can be constructed similarly with their respective loading vectors and slices: ${{{{\bf{v}}}}}^{(r)}\in {{\mathbb{R}}}^{T},\;{{{{{B}}}}}^{(r)}\in {{\mathbb{R}}}^{N\times K}$ for the time-slicing components and ${{{{\bf{w}}}}}^{(r)}\in {{\mathbb{R}}}^{K},\;{{{{{C}}}}}^{(r)}\in {{\mathbb{R}}}^{N\times T}$ for the trial-slicing components. Put together, this results in a decomposition of the following form:

4 $${\hat{X}}_{n,t,k}=\mathop{\sum }\limits_{r=1}^{{R}_{{{{\rm{neuron}}}}}}{u}_{n}^{(r)}{A}_{t,k}^{(r)}+\mathop{\sum }\limits_{r=1}^{{R}_{{{{\rm{time}}}}}}{v}_{t}^{(r)}{B}_{n,k}^{(r)}+\mathop{\sum }\limits_{r=1}^{{R}_{{{{\rm{trial}}}}}}{w}_{k}^{(r)}{C}_{n,t}^{\,(r)}$$

Because of the different slice types, each sliceTCA model can be described by the hyperparameter three-tuple *R* = (*R*_neuron_, *R*_trial_, *R*_time_), defining the number of neuron-, trial- and time-slicing components, for a total of *R*_neuron_ + *R*_trial_ + *R*_time_ components.

#### Relationship to TCA

The extension of matrix factorizations to TCA is based on a different definition of tensor rank, in which a rank-1 tensor is as an outer product of *d* vectors. Each component is defined by a set of vectors corresponding to neuron, time and trial coefficients ${{{{\bf{u}}}}}^{(r)}\in {{\mathbb{R}}}^{N}, {{{{\bf{v}}}}}^{(r)}\in {{\mathbb{R}}}^{T},{{{{\bf{w}}}}}^{(r)}\in {{\mathbb{R}}}^{K}$ for each component: *X*^(*r*)^ = **u**^(*r*)^ ⊗ **v**^(*r*)^ ⊗ **w**^(*r*)^. Then, each element of the approximated data tensor can be written as

5 $${\hat{X}}_{n,t,k}=\mathop{\sum }\limits_{r=1}^{R}{u}_{n}^{(r)}{v}_{t}^{(r)}{w}_{k}^{(r)}$$

In other words, a TCA component is a special case of a sliceTCA component in which the slice is a rank-1 matrix. In this way, sliceTCA is more flexible than TCA, as it has fewer constraints on the type of structure that is identified in the data. However, this increase in flexibility comes with the cost of an increased number of parameters, as sliceTCA fits all the entries of each slice. The flexibility of sliceTCA also leads to different invariance classes as discussed below. Finally, we note that the two methods can, in principle, be merged by incorporating TCA components into equation (4).

### SliceTCA invariance classes

#### Transformations within a slice type

Matrix factorization methods are known to be invariant to invertible linear transformations, including, but not limited to, rotations of the loading vectors. For example, suppose we decompose a matrix ${{{{Y}}}}\in {{\mathbb{R}}}^{N\times T}$ into a product of a matrix of weights, ${{{{W}}}}\in {{\mathbb{R}}}^{N\times R}$, and a matrix of scores, ${{{{S}}}}\in {{\mathbb{R}}}^{R\times T}$. Consider any invertible linear transformation ${{{{F}}}}\in {{\mathbb{R}}}^{R\times R}$. Then, *Y* can be rewritten as

6 $${{{{Y}}}}={{{{WS}}}}={{{{{WFF}}}}}^{\,-{{{{1}}}}}{{{{S}}}}=\tilde{{{{{W}}}}}\tilde{{{{{S}}}}}$$

where $\tilde{{{{{W}}}}}={{{{WF}}}}$ and $\tilde{{{{{S}}}}}={{{{{F}}}}}^{-{{{{1}}}}}{{{{S}}}}$. As a result, matrix decompositions, such as factor analysis, lead to not one solution but rather an invariance class of equivalent solutions. Note that PCA avoids this problem by aligning the first component to the direction of the maximum projected variance, as long as the eigenvalues of the covariance matrix are distinct. However, other methods that do not have a ranking of components are not able to use the same alignment. SliceTCA inherits this same invariance class, as all the loading vectors within a given slice type can be transformed in the same way as equation (6) to yield the same partially reconstructed tensor for each slice type (Extended Data Fig. 5a).

#### Transformations between slice types

SliceTCA has an additional invariance class due to the fundamental properties of multilinear addition. For example, consider a slice-rank-2 tensor ${{{{Y}}}}\in {{\mathbb{R}}}^{N\times T\times K}$, which is made of two components of different slice types. We will assume without loss of generality that these are neuron- and time-slicing components with corresponding slices *V* and *U*, such that

$${Y}_{n,t,k}={u}_{n}{V}_{t,k}+{v}_{t}{U}_{n,k}$$

Then, the following transformation can be performed for the arbitrary vector ${{{\bf{z}}}}\in {{\mathbb{R}}}^{K}$:

$$\begin{array}{rcl}{Y}_{n,t,k}&=&{u}_{n}{V}_{t,k}+{v}_{t}{U}_{n,k}+{u}_{n}{v}_{t}{z}_{k}-{u}_{n}{v}_{t}{z}_{k}\\ &=&{u}_{n}\left({V}_{t,k}-{v}_{t}{z}_{k}\right)+{v}_{t}\left({U}_{n,k}+{u}_{n}{z}_{k}\right)\\ &=&{u}_{n}{\tilde{V}}_{t,k}+{v}_{t}{\tilde{U}}_{n,k}\end{array}$$

where $\tilde{{{{{V}}}}}={{{{V}}}}-{{{\bf{v}}}}\otimes {{{\bf{z}}}}$ and $\tilde{{{{{U}}}}}={{{{U}}}}+{{{\bf{u}}}}\otimes {{{\bf{z}}}}$ are transformations of the original slices. This invariance class, therefore, corresponds to passing a tensor-rank-1 tensor between two slices of differing slice types (Extended Data Fig. 5b).

Note that two classes of transformations (within slice type and between slice type) commute (see proposition 2.1 of Supplementary mathematical notes); therefore, one cannot obtain a new transformation by, for example, applying the first transformation, followed by the second and then the first again.

#### Identification of a unique sliceTCA decomposition

To find a uniquely defined solution, we can take advantage of the natural hierarchy between the two invariance classes. Specifically, let us first define the partial reconstruction ${\hat{{X}}}^{\rm{neuron}}$ of the low-slice-rank approximation $\hat{{{{{X}}}}}$ based on the neuron-slicing components; that is

$${\hat{{{{{X}}}}}}^{{{{\rm{neuron}}}}}=\mathop{\sum }\limits_{r=1}^{{R}_{{{{\rm{neuron}}}}}}{{{{\bf{u}}}}}^{(r)}\otimes {{{{{A}}}}}^{(r)}$$

and let ${\hat{X}}^{\rm{time}}$ and ${\hat{X}}^{\rm{trial}}$ be similarly defined, so that ${\hat{X}}={\hat{X}}^{\rm{neuron}}+{\hat{X}}^{\rm{time}}+{\hat{X}}^{\rm{trial}}$. Now, note that the within-slice-type transformations change the weights of the loading vectors and slices of all components of a given slice type without changing the partial reconstructions for each slice type. For example, applying these transformations to the neuron-slicing components would change **u**^(*r*)^ and *A*^(*r*)^ but not ${\hat{X}}^{\rm{neuron}}$. On the contrary, the between-slice-type transformations change the partial reconstructions ${{\hat{X}}^{\rm{neuron}}},\,{{\hat{{X}}}^{\rm{time}}}$ and ${{\hat{X}}^{\rm{trial}}}$, but not the full reconstruction ${\hat{X}}$. Therefore, the key to identifying a unique solution is first to perform the between-slice-type transformations to identify the unique partial reconstructions ${{\hat{X}}^{\rm{neuron}}},\,{{\hat{X}}^{\rm{time}}}$ and ${\hat{X}}^{\rm{trial}}$ and then perform the within-slice-type transformations to identify the unique loading vectors and components.

We leveraged this hierarchy to develop a post hoc model optimization into three steps, each with a distinct loss function. The first step identifies a model that minimizes a loss function ${{{{\mathcal{L}}}}}_{1}$ defined on the full reconstruction (Extended Data Fig. 8c(i)), resulting in the approximation $\hat{{{{{X}}}}}$. Next, because of the two invariance classes, there is a continuous manifold of solutions with different parameters (loading vectors and slices) that, after being recombined, all result in the same $\hat{{{{{X}}}}}$ and, therefore, have the same loss. Next, we use stochastic gradient descent to identify the between-slice-type transformation that minimizes a secondary loss function ${{{{\mathcal{L}}}}}_{2}$, which fixes ${\hat{X}}^{\rm{neuron}},\;{\hat{X}}^{\rm{time}}$ and ${{\hat{X}}^{\rm{trial}}}$ without affecting ${\hat{X}}$ (Extended Data Fig. 8c(ii)). Finally, we identify the within-slice-type transformation that minimizes a tertiary loss function ${{{{\mathcal{L}}}}}_{3}$ to arrive at the final components (loading vectors **u**^(*r*)^, **v**^(*r*)^, **w**^(*r*)^ and slices *A*^(*r*)^, *B*^(*r*)^, *C*^(*r*)^) without affecting ${{\hat{X}}^{\rm{neuron}}},\,{{\hat{X}}^{\rm{trial}}}$ and ${{\hat{X}}^{\rm{time}}}$ (Extended Data Fig. 8c(iii)). Each of the three loss functions can, in principle, be chosen according to the constraints or normative assumptions most relevant to the question at hand.

We note that, if we performed only the ${{{{\mathcal{L}}}}}_{1}$ optimization step, then different initializations would lead to different solutions for the coefficients. Both the ${{{{\mathcal{L}}}}}_{2}$ and ${{{{\mathcal{L}}}}}_{3}$ steps are necessary to identify a unique solution across the two invariance classes. If we applied only ${{{{\mathcal{L}}}}}_{3}$ after ${{\mathcal{L}}_{1}}$, there would be no guarantee that ${{\hat{X}}^{\rm{neuron}}}$ would be the same for two seeds, as they could differ by more than just a rotation due to the between-slice-type invariances; therefore, it would not necessarily be possible to identify a unique solution. If we then applied ${{{{\mathcal{L}}}}}_{2}$ to correct this, we would need to reapply ${{{{\mathcal{L}}}}}_{3}$ to come up with a unique set of coefficients. Therefore, the most natural way to identify a unique solution is to exploit the hierarchical structure of the invariances by optimizing the invariances in the proposed order: ${{{{\mathcal{L}}}}}_{1}$, then ${{{{\mathcal{L}}}}}_{2}$, then ${{{{\mathcal{L}}}}}_{3}$. More precisely, we prove that, if each of these objective functions leads to a unique solution, the decomposition is unique under weak conditions (see theorem 2.7 in Supplementary mathematical notes).

This procedure can also be understood more intuitively by considering the case in which there is only a single component type, in which case sliceTCA reduces to a matrix factorization. Even then, minimizing ${{{{{{{{\mathcal{L}}}}}}}}}_{{{{{1}}}}}$ is not sufficient to determine a unique model due to there being a continuum of factor rotations that yield the same $\hat{{{{{X}}}}}$. PCA solves these invariances by constraining the factors to be orthogonal and ranking them by variance explained, resulting in a unique solution (under certain weak conditions, for example, up to sign reversals if all singular values are unique). This can be written through an additional loss function (equivalent to ${{{{\mathcal{L}}}}}_{3}$ in our framework). When considering mixed slice types, the second step (minimizing ${{{{\mathcal{L}}}}}_{2}$) becomes necessary owing to the invariant transformations between slice types.

### Model selection, optimization and fitting

To fit sliceTCA for a given dataset arranged as a 3-tensor, we followed the data analysis pipeline described in the main text. Below, we provide details and hyperparameters for the steps involved in the pipeline.

#### Fitting sliceTCA with stochastic gradient descent

For a fixed choice of *R*, model parameters (that is, the loading vectors and slices of all components) were fitted using the optimizer Adam^54^ in Pytorch. Initial parameters were randomly drawn from a uniform distribution over [−1, 1] or [0, 1] for unconstrained and non-negative sliceTCA, respectively. Throughout, we optimized the MSE loss in equation (3) with a learning rate of 0.02. Note that, during model selection, some of these entries will be masked (that is, not be summed in the loss) for cross-validation (see the next section). To introduce stochasticity in the computation of the gradient, and thus avoid local minima, we additionally masked a fraction of tensor entries so that they are not included in the calculation of the loss. This fraction starts at 80% and decreases exponentially during training with a decay factor of 0.5 over three (Fig. 2) or five blocks of iterations (Figs. 4 and 5). Within each block, the mask indices are randomly reinitialized every 20 of a total of 150 (Fig. 2), 200 (Fig. 4) or 100 iterations per block (Fig. 5). Run time scales approximately linearly with the number of components (Supplementary Fig. 16). To obtain an optimal model under a given *R*, we repeated the fitting procedure ten times with different random seeds and chose the model with the lowest loss.

#### Cross-validated model selection

To choose the number of components in each slice type, we run a 3D grid search to optimize the cross-validated loss. In addition to the decaying mask used during model fitting, we masked 20% of the entries throughout the fitting procedure as held-out data. These masked entries were chosen in randomly selected 1-s (Fig. 4) or 150-ms blocks (Fig. 5) of consecutive time points in random neurons and trials. Blocked masking of held-out data (rather than salt-and-pepper masking) was necessary to avoid temporal correlations between the training and testing data due to the slow timescale of the calcium indicator or due to smoothing effects in electrophysiological data. To protect further against spuriously high cross-validation performance due to temporal correlations, we trimmed the first and last 250 ms (Fig. 4) or 40 ms (Fig. 5) from each block; these data were discarded from the test set, and only the remaining interior of each block was used to calculate the cross-validated loss. We repeated the grid search ten times with different random seeds for train–test split and parameter initialization while keeping a constant seed for different *R*. Once the cross-validated grid search was complete, we selected R^*^ by identifying the model with a minimum or near-optimal average test loss across seeds. Admissible models are defined as those achieving a minimum of 80% of the optimal performance for nonconstrained sliceTCA and 95% of the optimal model performance for non-negative sliceTCA, as compared to root-mean-squared entries of the raw data.

#### Hierarchical model optimization

For the first step of the model optimization procedure, we chose the MSE loss for ${{\mathcal{L}}_{1}}$:

$$\begin{array}{l}{\mathcal{L}}_1({{\mathbf{u}}},{A},{\mathbf{v}},{B},{\mathbf{w}},{C})\\=\displaystyle\frac{1}{KNT}\left\|{{{X}}}-\left(\sum\limits_{r {=} 1}^{R_{\mathrm{neuron}}}{[}{\mathbf{u}}^{(r)}\otimes {A}^{(r)}{]}+\sum\limits_{r = 1}^{R_{\mathrm{time}}}{[}{\mathbf{v}}^{(r)}\otimes {B}^{(r)}{]}+\sum\limits_{r {=} 1}^{R_{\mathrm{trial}}}{[}{\mathbf{w}}^{(r)}\otimes {C}^{(r)}{]}\right)\right\|^2_F\end{array}$$

as in the model selection (essentially refitting the model with the specific ranks identified with the cross-validation procedure on the entire data). For ${{{{\mathcal{L}}}}}_{2}$, we used the sum of the squared entries of the three partial reconstructions from each slice type:

$$\begin{array}{rcl} {\mathcal{L}}_2({\mathbf{x}},{\mathbf{y}},{\mathbf{z}})&=& \left\|{{\hat{X}}}^{\mathrm{trial}}-\sum\limits_{r,s}{\mathbf{x}}^{(r,s)}\otimes {\mathbf{v}}^{(s)} \otimes {\mathbf{w}}^{(r)}-\sum\limits_{r,s}{\mathbf{u}}^{(r)} \otimes {\mathbf{y}}^{(r,s)} \otimes {\mathbf{w}}^{(s)}\right\|^2_F\\ &+&\left\| {\hat{X}}^{\mathrm{time}}+\sum\limits_{r,s}{\mathbf{x}}^{(r,s)} \otimes {\mathbf{v}}^{(s)}\otimes {\mathbf{w}}^{(r)}-\sum\limits_{r,s}{{\mathbf{u}}}^{(r)} \otimes {{\mathbf{v}}}^{(s)} \otimes {{\mathbf{z}}}^{(r,s)}\right\|^2_F\\ &+&\left\| {\hat{X}}^{{\mathrm{neuron}}}+\sum\limits_{r,s}{{\mathbf{u}}}^{(r)}\otimes {{\mathbf{y}}}^{(r,s)}\otimes {{\mathbf{w}}}^{(s)}+\sum\limits_{r,s}{{\mathbf{u}}}^{(r)}\otimes {{\mathbf{v}}}^{(s)}\otimes {{\mathbf{z}}}^{(r,s)}\right\|^2_F \end{array}$$

where ${{{\bf{x}}}}\in {{\mathbb{R}}}^{{R}_{{{{\rm{time}}}}}\times {R}_{{{{\rm{trial}}}}}\times N},\;{{{\bf{y}}}}\in {{\mathbb{R}}}^{{R}_{{{{\rm{neuron}}}}}\times {R}_{{{{\rm{trial}}}}}\times T}$ and ${{{\bf{z}}}}\in {{\mathbb{R}}}^{{R}_{{{{\rm{neuron}}}}}\times {R}_{{{{\rm{time}}}}}\times K}$. This can be thought as a form of L2 regularization. For ${{{{\mathcal{L}}}}}_{3}$, we chose orthogonalization and variance explained ordering through singular value decomposition (SVD).

We stress that the losses ${{\mathcal{L}}_{1}},\,{{\mathcal{L}}_{2}}$ and ${{{{\mathcal{L}}}}}_{3}$ may be chosen according to the specific problem at hand. For example, different factor rotations could be easily implemented into the hierarchical model optimization, including varimax or even oblique (that is, nonorthogonal) rotations. Therefore, while we chose an ${{{{\mathcal{L}}}}}_{3}$ that constrained components to be orthogonal, in general, sliceTCA does not necessarily need to return orthogonal components. Finally, we remark that the hierarchical model optimization procedure is valid only for unconstrained sliceTCA, as adding a non-negativity constraint restricts the possible space of solutions. This also explains why non-negative factorizations (for example, NMF) are known to suffer less from uniqueness issues but also require more complex conditions to guarantee uniqueness^30^. Future work could borrow from existing methods for factor rotations specifically designed for NMF to extend to non-negative sliceTCA^55^.

#### Model similarity

To estimate whether solutions found with sliceTCA are unique in practice, we adopted a measure of the model similarity of the solutions found from different random initializations^4,56^. This score is based on computing the angle between a pair of vectors corresponding to the loading factors of two models after components are matched according to the Hungarian algorithm. For each pair of sliceTCA components, we unfolded the slice of each component into a vector. Then, we computed the angle between the loading vectors, the angle between the vectors resulting from unfolded slices, and their average values.

Following previous work^4^, we computed this modified similarity score for each of the ten randomly initialized models against the model that achieved the lowest MSE loss. We calculated (1) the overall model similarity and (2) the model similarity for each slice type, which could be an informative diagnostic tool for model optimization in future work. To establish a baseline chance level of similarity, we also computed a shuffled model similarity score: for each slice type and component, we shuffled the elements of the weight vectors of one of the two models within the respective weight vectors before computing their similarity score. We then calculated the mean similarity over 100 shuffle repetitions for each slice type.

### Feedforward model of perceptual learning

We modeled a population of linear neurons receiving a sensory input from upstream sources representing a go stimulus and a no-go stimulus, as well as an input representing a top–down modulation that varied from trial to trial. On each trial *k*, either the go or no-go stimulus was activated, with probability *P* = 0.5 of presenting the same stimulus as in the previous trial. Go/no-go inputs (*x*^go^, *x*^no^) were assumed to follow the same bell-shaped activation function ${s}_{t}={e}^{-{(t-4)}^{2}}$ on the trials during which their corresponding stimulus was presented, that is, ${x}_{t,k}^{\,{\textrm{go}}}={s}_{t}$ if *k* was a go trial and ${x}_{t,k}^{\textrm{go}}=0$ otherwise (and vice versa for the no-go input).

The stochastic learning process of the go and no-go weights ${{{{\bf{w}}}}}_{k}^{\textrm{go}},{{{{\bf{w}}}}}_{k}^{\textrm {no}}\in {{\mathbb{R}}}^{N}$ over trials was modeled as an Ornstein–Uhlenbeck process, which was initialized at ${{{{\bf{w}}}}}_{0}^{\textrm{go}}={{{{\bf{w}}}}}_{0}^{\textrm{no}}=\bf{1}$ and evolved independently across neurons:

$$\begin{array}{rcl}{{{{\textrm{d}}{{\mathbf{w}}}}}}_{k}^{\textrm {go}}&=&{{{{\textrm{diag}(\boldsymbol{\alpha}) }}}}\left({\mu }^{\textrm{go}}-{{{{{\mathbf{w}}}}}}_{k}^{\textrm{go}}\right){\textrm{d}}k+\sigma {{{{{{\textrm{{d}}}\mathbf{W}}}}}}_{k}\\ {{{{\textrm{d}}{{\mathbf{w}}}}}}_{k}^{\textrm{no}}&=&{{{{\textrm{diag}(\boldsymbol{\alpha})}}}}\left({\mu }^{\textrm{no}}-{{{{{\mathbf{w}}}}}}_{k}^{\textrm{no}}\right){\textrm{d}}k+\sigma {{{{{{\textrm{{d}}}\mathbf{W}}}}}}_{k}\end{array}$$

where ${\alpha }_{n} \sim {{{\mathcal{U}}}}([0.2,0.8])$ are the neuron-specific learning rates, and *μ*^go^ = 2, *μ*^no^ = 0, *σ* = 1.3. Furthermore, to keep weights non-negative and simulate their saturation, we clamped them to [0, 2]. The process was evaluated using a stochastic differential equation solver and sampled at *K* evenly spaced points in [0, 10] representing *K* trials.

Top–down modulation was modeled as a rectified Gaussian process:

$${x}_{t,k}^{\textrm{TD}}=\max (0,\gamma (t)),\quad \gamma \sim GP(0,\kappa )$$

with the temporal kernel:

$$\kappa ({t}_{1},{t}_{2})=\exp \left(-\frac{{({t}_{1}-{t}_{2})}^{2}}{2{l}^{2}}\right)$$

where $l=\sqrt{0.5}$. Top–down weights were nonplastic and distributed as ${w}_{n}^{\textrm{TD}} \sim {{{\mathcal{U}}}}([0,1])$.

The activity of each neuron was thus given by

$$\begin{array}{rcl}{X}_{n,t,k}&=&{w}_{n,k}^{\textrm{go}}{x}_{t}^{\,{\textrm{go}}}+{w}_{n,k}^{\textrm{no}}{x}_{t}^{\textrm{no}}+{w}_{n}^{\textrm{TD}}{x}_{t,k}^{\textrm{TD}}\\ &=&{w}_{n,k}^{\textrm{S}}{s}_{t}+{w}_{n}^{\textrm{TD}}{x}_{t,k}^{\textrm{TD}}\end{array}$$

where the sensory input is combined into ${w}_{n,k}^{\textrm{S}}={w}_{n,k}^{\textrm{go}}{{\mathbb{1}}}_{k}^{\textrm{go}}+{w}_{n,k}^{\textrm{no}}(1-{{\mathbb{1}}}_{k}^{\textrm{go}})$, where ${{\mathbb{1}}}^{\textrm{go}}$ is an indicator function that is 1 when trial *k* is a go trial and 0 if it is a no-go trial. By construction, the tensor *X* has a slice rank of 2, as it can be written in the following form:

$${X}={{I}}^{\textrm{S}}+{I}^{\textrm{TD}}$$

where ${I}_{n,t,k}^{\,{\textrm{S}}}={w}_{n,k}^{\textrm{S}}{s}_{t}$ is a time-slicing component representing the weighted, trial-specific sensory input and ${I}_{n,t,k}^{\textrm{TD}}={w}_{n}^{\textrm{TD}}{x}_{t,k}^{\textrm{TD}}$ is a neuron-slicing component representing top–down modulatory factors that vary over trials. In our simulations, we used *K* = 100, *T* = 90, *N* = 80.

We fitted sliceTCA with non-negativity constraints to the synthetic dataset, using five blocks of 200 iterations each with a learning rate that decayed exponentially over blocks from 0.2 to 0.0125 and a mask that decayed exponentially over blocks from 0.8 to 0.05. Masked entries changed randomly every iteration. Initial parameters were drawn uniformly over [0, 1].

### RNN model of condition-dependent neural sequences

#### Model description

We built a model of a linear RNN that produces recurrently generated sequences for different task conditions while also receiving condition-independent inputs. To generate sequences, we parameterize the connectivity matrix $W\in {{\mathbb{R}}}^{N\times N}$ by a Schur decomposition^24^. Additionally, we let the central matrix have a block-diagonal structure to embed multiple sequences into the dynamics. Formally, we let *W* = *USU*^*T*^, where *U* is a unitary matrix and *S* is defined in block structure as

$$S=\left[\begin{array}{cc}(\lambda +\epsilon ){I}_{-}-\lambda I&{{{{0}}}}\\ {{{{0}}}}&(\lambda +\epsilon ){I}_{-}-\lambda I\end{array}\right]$$

where $I\in {{\mathbb{R}}}^{N/2\times N/2}$ is the identity matrix and ${I}_{-}\in {{\mathbb{R}}}^{N/2\times N/2}$ is the matrix with ones along its subdiagonal. The unitary matrix *U* was generated as the left singular vector matrix of a random normal matrix.

Each block of *S* corresponds to the sequential dynamics for one of the two noninterfering sequences. The specific sequence is selected by the initial state of the network. This is parameterized through the first and (*N*/2 + 1)th columns of *U* (that is, *U*_1_ and *U*_*N*__/2+1_), which correspond to the beginning of each sequence. The RNN also receives a 2D input that is condition independent. To avoid interference with the sequences, we mapped the input through the (*N*/2)th and the *N*th columns of *U* (that is, *U*_*N*__/2_ and *U*_*N*_), as these are the elements corresponding to the end of the sequence. In this way, we were able to generate RNN dynamics that produce condition-specific sequences while also being influenced by condition-independent inputs.

To test the effects of different sources of noise, we considered RNN dynamics that are governed by stochastic differential equations. On trial *k*, the population activity ${{{{\bf{x}}}}}^{(k)}(t)\in {{\mathbb{R}}}^{N}$ and inputs ${{{{\bf{u}}}}}^{(k)}(t)\in {{\mathbb{R}}}^{2}$ evolve according to

$$\left\{\!\!\begin{array}{ll}{{{{\textrm{d}}{\bf{x}}}}}^{(k)}=(-{{{{\bf{x}}}}}^{(k)}+W\phi ({{{{\bf{x}}}}}^{(k)})+B{{{{\bf{u}}}}}^{(k)}){\textrm{d}}t+{\sigma }_{1}{{{{\textrm{d}}{{W}}}}}_{1}^{(k)} &{{{{\bf{x}}}}}^{(k)}(0)={c}_{1}^{(k)}{U}_{1}+{c}_{2}^{(k)}{U}_{N/2+1}\\ {{{{\textrm{d}}{\bf{u}}}}}^{(k)}={A}^{(k)}{{{{\bf{u}}}}}^{(k)}{\textrm{d}}t+{\sigma }_{2}{{{{\textrm{d}}{{W}}}}}_{2}^{\,(k)} &{{{{\bf{u}}}}}^{(k)}(0)={{{{{u}}}}}_{0}^{(k)}\end{array}\right.$$

where $B=[{U}_{N/2},{U}_{N}],\;{A}_{ij} \sim {{{\mathcal{N}}}}(0,1/2)$ and d*W*_*i*_ are infinitesimal increments of a Wiener process. Furthermore, we took $[{c}_{1}^{(k)},{c}_{1}^{(k)}]=[\cos ({\theta }^{(k)}),\sin ({\theta }^{(k)})]$, where *θ*^(*k*)^ represents the angle of the task variable. In our simulations, we used *K* = *T* = *N* = 200 and took *ϕ* = id.

RNNs have three natural sources of noise: (1) noise at the level of the dynamics of each neuron, *σ*_1_d*W*_1_, which we call intrinsic noise; (2) input noise, *σ*_2_d*W*_2_; and (3) observation noise added to the full tensor, *Y* = *X* + *η*, where ${{{{{\eta }}}}}_{ijk} \sim {{{\mathcal{N}}}}(0,{\sigma }_{3})$. Thus, by systematically varying *σ*_1_, *σ*_2_, *σ*_3_, we can vary the magnitude of different sources of noise in the data. Importantly, they have the property that for *ϕ* = id, ${\mathbb{E}}[{{{{Y}}}}\;]={{{{X}}}}$, where **y**^(*k*)^(*t*) is the activity with *σ*_*i*_ ≠ 0 for at least one *i* and **x**^(*k*)^(*t*) is the activity with *σ*_*i*_ = 0 for all *i*.

To evaluate the effect of these different sources of noise on sliceTCA, we considered the variance explained $\kappa =1-| | \hat{{{{{Y}}}}}-{{{{Y}}}}| {| }_{F}^{2}/| | {{{{Y}}}}-\bar{y}| {| }_{F}^{2}$ as a function of the noise level $\zeta =| | {{{{Y}}}}-{{{{X}}}}| {| }_{F}^{2}/| | {{{{Y}}}}-\bar{y}| {| }_{F}^{2}$, where $\hat{{{{{Y}}}}}$ is the reconstruction from sliceTCA fit on *Y*. In the normalization term above, $\bar{y}\in {\mathbb{R}}$ is the average over all *NTK* entries of *Y* (but we note that different marginalizations are possible^57^). An optimal denoiser (that is, for which $\hat{{{{{Y}}}}}={{{{X}}}}$) would yield *κ* = 1 − *ζ*. Meanwhile, a model that fully captures the variability (including noise) in the data (that is, $\hat{{{{{Y}}}}}={{{{Y}}}}$) would have *κ* = 1.

### Statistics and reproducibility

As we reanalyzed existing data, no statistical method was used to predetermine sample sizes. Instead, we demonstrated the utility of sliceTCA by choosing three previously published datasets representing a typical range of numbers of recorded neurons, time points and trials. For the application of sliceTCA to these example datasets and subsequent analyses, we randomly selected an example session and animal for each dataset. General trends were confirmed by fitting sliceTCA on other example sessions of the same dataset (not shown). To ensure reproducibility, we have made available the datasets for the sessions analyzed in this paper, along with the analysis code (see the ‘Data availability’ and ‘Code availability’ sections below). Model selection was performed as described in the ‘Model selection, optimization and fitting’ section above. During cross-validation, tensor entries (indexed by neurons, trials and blocks of time) were randomly allocated (80–20%) into training versus held-out data using a pseudo-random number generator. No blinding was performed, as our method is unsupervised and was applied to the full dataset. The investigators were not blinded to outcome assessment. Unless otherwise specified, we performed two-sided nonparametric statistical tests. In Extended Data Fig. 10d, model assumptions were not tested before performing analyses of variance. In dataset 3, we excluded neurons with low firing rates (<0.2 Hz); otherwise, no data were excluded from the analyses.

### Dataset 1 of motor cortical recordings during a center-out and maze reaching task

#### Description of the dataset

We analyzed a dataset of motor cortical (M1, *n* = 90) and premotor cortical (PMd, *n* = 92) electrophysiological recordings^17^. The dataset is curated and publicly available as part of the ‘Neural Latents Benchmark’ project^58^. Briefly, monkeys were trained to perform a delayed center-out reach task to one of 27 locations in both the maze condition (in which barriers were placed on the screen, leading to curved optimal reach trajectories) and the no-maze condition with matched target locations (classic center-out task leading to straight optimal reach trajectories). The go signal for movement initiation appeared 0–1,000 ms after target onset and 1,000–2,600 ms after the trial started with a fixation cue. We analyzed data from one animal (monkey J) in a single session and randomly subselected 12 target locations, resulting in *K* = 246 single-target trials in the maze reach conditions and *K* = 265 single-target trials in the 12 center-out reach conditions with matched target locations.

#### Additional preprocessing

We calculated firing rates for bins of 10 ms, which we then smoothed with a Gaussian filter with *σ* = 20 ms and rescaled to minimum and maximum values of 0 and 1 over the course of the experiment for each neuron separately. We selected a time period starting 1 s before movement onset (thus including a substantial part of the motor preparation period) and ending 0.5 s after movement onset when the monkey had successfully reached the target position in most trials. We did not time-warp the data. The resulting data tensor had dimensions of *N* = 182, *T* = 150 and *K* = 511.

#### Supervised mapping of neural population activity onto kinematic data

To identify the neural subspace from which 2D hand trajectories could be read out (Fig. 2a), we used ordinary least squares (OLS). Specifically, we found weights that project the neuron-unfolded data from the full neural space onto a 2D subspace that best maps onto (*x*, *y*) hand velocity with a time delay of 100 ms to account for the lag between neural activity and movement. When testing the decoding analysis after dimensionality reduction, we instead applied OLS to the reconstruction (or partial reconstruction (that is, from a single slice type)) after reshaping it into an *N* × *KT* matrix. We also used OLS to project time-averaged pre-movement activity onto target locations (Fig. 2g). For Fig. 2h, we used LDA to identify the dimension that best separates pre-movement averaged activity in clockwise versus counterclockwise curved reaches in the maze condition. To plot activity in a 3D neural subspace that contained information about the upcoming movement, we then orthogonalized the two axes that map neural activity onto target locations to the axis that distinguishes clockwise and counterclockwise movements.

For all decoding analyses, we calculated *R*^2^ values on left-out trials in a fivefold cross-validation procedure performed on 20 permutations of the trials. Decoding was performed on data from the period spanning 250 ms before to 450 ms after movement onset. For trial-resolved data (Fig. 2a, raw data, neuron-slicing NMF, TCA, trial-slicing NMF), we averaged trial-wise *R*^2^ values; for pre-movement information on target positions, we calculated a single *R*^2^ value across trials for center-out and maze reaching conditions. For trial-averaged data (Fig. 2a, trial-averaged raw data), we performed twofold cross-validation by averaging hand and neural trajectories separately for each fold and then calculating *R*^2^ values averaged over conditions and folds.

#### Visualization of sliceTCA weights

The results of fitting non-negative sliceTCA are shown in Fig. 2c,d and Supplementary Fig. 3. Each component consists of a weight vector and a slice of corresponding weights on the other two variables. Along the trial dimension, we sorted trials by the angle of the target position and whether trials belonged to center-out or maze reaching conditions. Along the neuron dimension of trial-slicing components, neurons were sorted by the peak latency of neural activity in the first component. For the time-slicing component, neurons were sorted according to their mean activity in the first reaching condition.

#### Correlation matrices

To assess the encoding similarity of movement preparation in the time-slicing component, we calculated the *K* × *K* correlation matrix of the neural encoding weights (that is, the rows of the slice in Fig. 2d) for different pairs of trials, separately for center-out and maze reach conditions, and for the PMd (Fig. 2f) and M1 (Extended Data Fig. 4c). We sorted the resulting correlation matrices by the angle of the target location (Fig. 2f).

### Dataset 2 of cortico-cerebellar calcium imaging during a motor task

#### Description of the dataset

We analyzed recently published calcium imaging data consisting of simultaneously recorded cerebellar granule cells (*n* = 134) and premotor cortical L5 pyramidal cells (*n* = 152) from a head-fixed mouse performing a motor task in which a manipulandum had to be moved forward and leftward or rightward for a reward^18^. After a correct movement was completed, a water reward was delivered with a 1-s delay, followed by an additional 3.5-s intertrial interval. Left versus right rewarded turn directions were alternated without a cue after 40 successful trials. We analyzed data from one session of a mouse in an advanced stage of learning, comprising a total of *K* = 218 trials. The data were sampled at a 30-Hz frame rate. Calcium traces were corrected for slow drifts, *z*-scored and low-pass filtered^18^.

#### Additional preprocessing

Owing to the freely timed movement period, we piecewise linearly warped data to the median interval lengths between movement onset, turn and movement end. The remaining trial periods were left unwarped and cut to include data from 1.5 s before movement onset until 2.5 s after reward delivery, resulting in a preprocessed *N* × *T* × *K* data tensor with *N* = 286, *T* = 150 and *K* = 218.

#### Visualization of sliceTCA weights

In Fig. 4b,c, we show the results of a fitted sliceTCA model. We further reordered trials in the trial–time slices according to trial type and neurons in the neuron–time slices according to the peak activity in the first trial-loading component. This allows for a visual comparison of the tiling structure across components. We used Mann–Whitney *U* tests on the time-averaged activity between reward and trial end in the trial–time slices. We used LDA to determine the classification accuracy for neuron identity (cerebellum versus cortex) based on the loading vector weights of the three neuron-slicing components found by sliceTCA. We similarly reported the classification accuracy of trial identity (error versus correct, left versus right) based on the loading vector weights of the trial-slicing components.

#### Matrix rank of slices

To determine whether sliceTCA finds components with higher matrix ranks compared to methods that do not demix slice types (neuron-slicing PCA and factor analysis with neuron loadings, neuron- and time-concatenated PCA and factor analysis with trial loadings), we performed SVD on the six slices (after centering) of the sliceTCA model shown in Fig. 4b, as well as on the scores of either trial-slicing or neuron-slicing PCA and factor analysis, after refolding the resulting scores into *N* × *T* or *K* × *T* matrices, respectively. We then compared these to the spectra of squared singular values obtained from the slices of the trial-slicing (Fig. 4e) or neuron-slicing components (Supplementary Fig. 8). Factor analysis was performed using the ‘sklearn’ Python package^59^, which uses an SVD-based solver. For comparability with PCA and sliceTCA solutions, no factor rotations were performed.

#### Manifolds from sliceTCA reconstructions

To analyze the geometry of neural data, we reconstructed the low-slice-rank approximation of neural activity from the sliceTCA model separately for the cerebellum and premotor cortex. We then used LDA on both raw and reconstructed data to find the three axes that maximally separate left versus right correct trials between movement onset and reward (axis 1, Fig. 4g), movement onset time versus the time of reward in all correct trials (axis 2), and the time of reward versus post-reward (axis 3). We orthonormalized the three axes and projected raw and reconstructed data onto the new, 3D basis (Fig. 4h).

We then measured the distance ratio to compare the distance between trials of the same class versus the distance between trials of distinct classes (left versus right) in the full neural space. For the reconstructed versus the full dataset, we averaged neural activity over a 650-ms window centered at movement onset and measured the Euclidean distance of the population response in each trial to the trial-averaged population response in its own trial type, compared to the Euclidean distance to the average population response of the respective other trial type: *Δ*_between_/*Δ*_within_, where ${\varDelta}_{{{{\rm{within}}}}}={d}({x}_{k}^{\,{L}},{\bar{x}}^{\,{L}})$ is the Euclidean distance between population vectors in each left trial to the mean population vector across all left trials (and vice versa for right trials), and ${\varDelta }_{\rm{between}}={d}({x}_{k}^{\,{L}},{\bar{x}}^{R})$ is the Euclidean distance of population vectors in each left trial to the mean population vector across all right trials (and vice versa for right trials).

### Dataset 3 of electrophysiology across many brain regions during perceptual decision-making

#### Description of the dataset

The third analyzed dataset comprised recently published multiregion Neuropixels recordings (*n* = 303) in mice performing a perceptual decision-making task^53^. In the task, mice were presented a grating patch image with varying contrast (0%, 25%, 35%, 50% or 100%), shown on the left or right sides of a screen. The mice were trained to move the image to the center of the screen using a steering wheel within a 60-s period to receive a sugary water reward. A correct response was registered if the stimulus was moved to the center, whereas an incorrect response was recorded if the stimulus was moved to the border of the screen. We selected a single example mouse (subject CSHL049 from the openly released electrophysiology data repository).

#### Additional preprocessing

We binned single-neuron spiking events in 10-ms windows. Owing to the variable response times across trials, we piecewise linearly warped data between stimulus onset and reward delivery or timeout onset to correspond to the median interval length and clipped the trial period to start 1 s before stimulus onset and to end 2 s after reward delivery or timeout onset. We smoothed data with a Gaussian filter with *σ* = 20 ms and rescaled the activity of each neuron to a minimal and maximal value of 0 and 1 over all trials. We excluded neurons with mean firing rates below 0.2 Hz, leading to a total of *n* = 221 neurons analyzed of *n* = 303 neurons recorded. Brain regions included the visual cortex (anterior layers 2/3, 4, 5, 6a and 6b as well as anteromedial layers 2/3, 4, 5 and 6a; *n* = 85 neurons), hippocampal regions CA1 (*n* = 32 neurons) and dentate gyrus (molecular, polymorph and granule cell layers; *n* = 21 neurons), thalamus (including the posterior limiting nucleus and lateral posterior nucleus; *n* = 18 neurons) and the anterior pretectal and midbrain reticular nucleus (anterior pretectal nucleus, *n* = 22 neurons; midbrain reticular nucleus, *n* = 35 neurons) of the midbrain. In total, the resulting data tensor had dimensions of *N* = 221, *T* = 350 and *K* = 831.

#### Visualization of sliceTCA weights

In Fig. 5b, we scaled the rows of the neuron–time slices to a [0, 1] interval to highlight differences in the timing of peak activity between neurons. We then reordered neuron–time slices by the peak activity within each region for each slice type separately to show characteristic differences between neural correlates of behavioral variables. Trial–time slices were regrouped by trial type to show region-specific representations of task variables. Finally, neuron–trial slices were reordered by the average weights across the first 100 trials for each neuron within a region.

#### Reconstruction performance and component weights

For each neuron, we estimated the goodness of fit of the sliceTCA reconstruction as

$$1-\frac{{\sum }_{t,k}{\left({X}_{n,t,k}-{\hat{X}}_{n,t,k}\right)}^{2}}{{\sum }_{t,k}{X}_{n,t,k}^{\,{2}}}$$

We then quantified the contribution of the neuron-slicing components on the total sliceTCA reconstruction for each neuron *n* as the following ratio:

$${f}_{n}^{\;{\rm{neuron}}}=\frac{{\sum }_{t,k}{\hat{X}}_{n,t,k}^{\rm{neuron}}}{{\sum }_{t,k}{\hat{X}}_{n,t,k}}$$

where ${\hat{X}}^{\rm{neuron}}$ describes the partial reconstruction of the data tensor from only the neuron-slicing components. We similarly defined the contributions of the time- and trial-slicing components to the sliceTCA reconstruction of each neuron *n* as ${f}_{n}^{\rm{time}}$ and ${f}_{n}^{\rm{trial}}$.

### Reporting summary

Further information on research design is available in the Nature Portfolio Reporting Summary linked to this article.

## Online content

Any methods, additional references, Nature Portfolio reporting summaries, source data, extended data, supplementary information, acknowledgements, peer review information; details of author contributions and competing interests; and statements of data and code availability are available at 10.1038/s41593-024-01626-2.

## Supplementary information

Supplementary Information Supplementary Figs. 1–16 and mathematical notes.

Reporting Summary

## Data Availability

The datasets presented in this paper are available via figshare at 10.6084/m9.figshare.24961917.v1 (ref. ^60^). Source data are available via GitHub at https://github.com/caycogajiclab/sliceTCA_paper (ref. ^61^).

## Code availability

A GPU-accelerated Python library for the sliceTCA data analysis pipeline (including preprocessing, model selection, model optimization and visualization of components) is available as a pip installable package at https://github.com/arthur-pe/slicetca (ref. ^62^). Additionally, code for the analyses in this paper is available at https://github.com/caycogajiclab/sliceTCA_paper (ref. ^61^).
