# Supplementary material for: Dimensionality reduction beyond neural subspaces with slice tensor component analysis
Source: Nat Neurosci. 2024 May 6;27(6):1199–210. doi: 10.1038/s41593-024-01626-2 (PMC11537991; doi:10.1038/s41593-024-01626-2)
Supplement: Supplementary file 1 — Supplementary Figs. 1–16 and mathematical notes. [file 41593_2024_1626_MOESM1_ESM.pdf]

nature neuroscience

Technical Report

https://doi.org/10.1038/s41593-024-01626-2

Dimensionality reduction beyond neural subspaces with slice tensor component analysis

In the format provided by the authors and unedited

Supplementary Material

Dimensionality reduction beyond neural subspaces with slice tensor component analysis

Arthur Pellegrino[1,2], Heike Stein[1], and N Alex Cayco-Gajic[2]

[1]Equal contribution, [2]Correspondence

Contents

I Supplementary figures **2**

II Mathematical notes on SliceTCA **18**

1 Definition and motivation **18**

2 Invariances and uniqueness of decompositions **18**

3 Convexity of transformations **22**

Part I
Supplementary figures

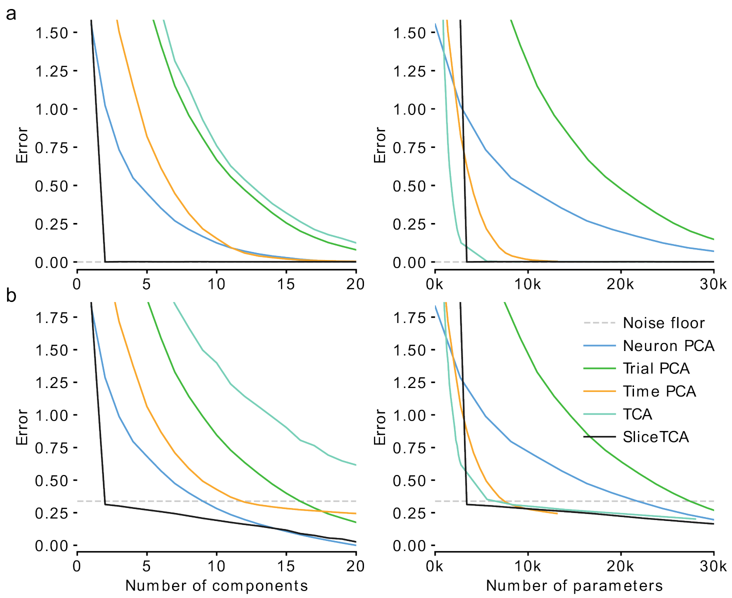

Supplementary Figure 1: Component vs. parameter efficiency of tensor decompositions. a. Losses as a function of the number of components (left) and parameters (right) for sliceTCA, TCA, and PCA for all unfoldings applied to the linear feedforward model (Figure 1e). In all cases, sliceTCA captures the simulated data with fewer components and parameters. Noise-free model. **b.** Same as panel a for the model with Gaussian noise.

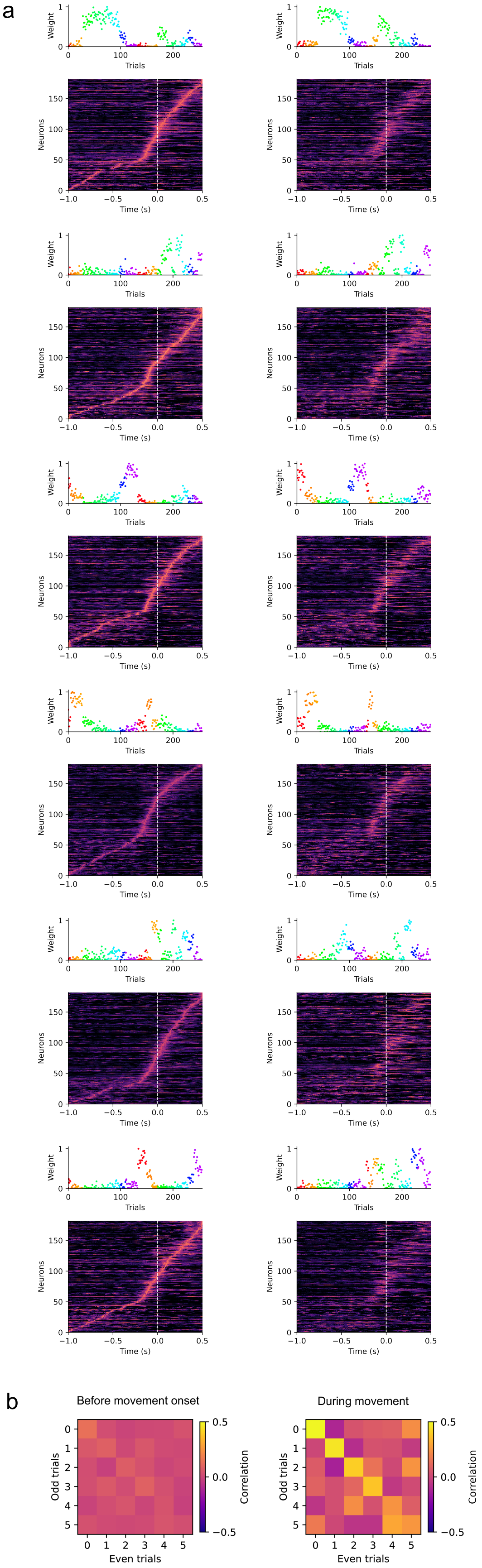

Supplementary Figure 2: Cross-validation of neuron-specific sequences identified in trial-slicing components. a. Components identified by trial-slicing NMF ($R_{\mathrm{neuron}} = 6$) applied to the motor cortical reaching dataset (Figure 2). To validate the sequences we separated the data into even (left) and odd (right) trials and fit the model separately on each of the two datasets (only 6 rather than 12 components to avoid overfitting since we halved the datasets). Components from the two models were matched by hand based on similarity of the trial loading vector. In each row of the panel, neurons in the slices are sorted identically (according to the latency of peak activity in even trials). Neural sequences during movement (dashed white line indicates movement onset) are reproduced in the two independent groups of trials, while pre-motor sequences are not matched. **b.** To quantify how reliably movement-related and pre-motor sequences could be identified across splits, for each component we calculated the Pearson correlation for each pair of slices across the two models, separately for pre-movement onset (-1 to -0.2 s) and during movement (-0.1 - 0.5 s) periods.

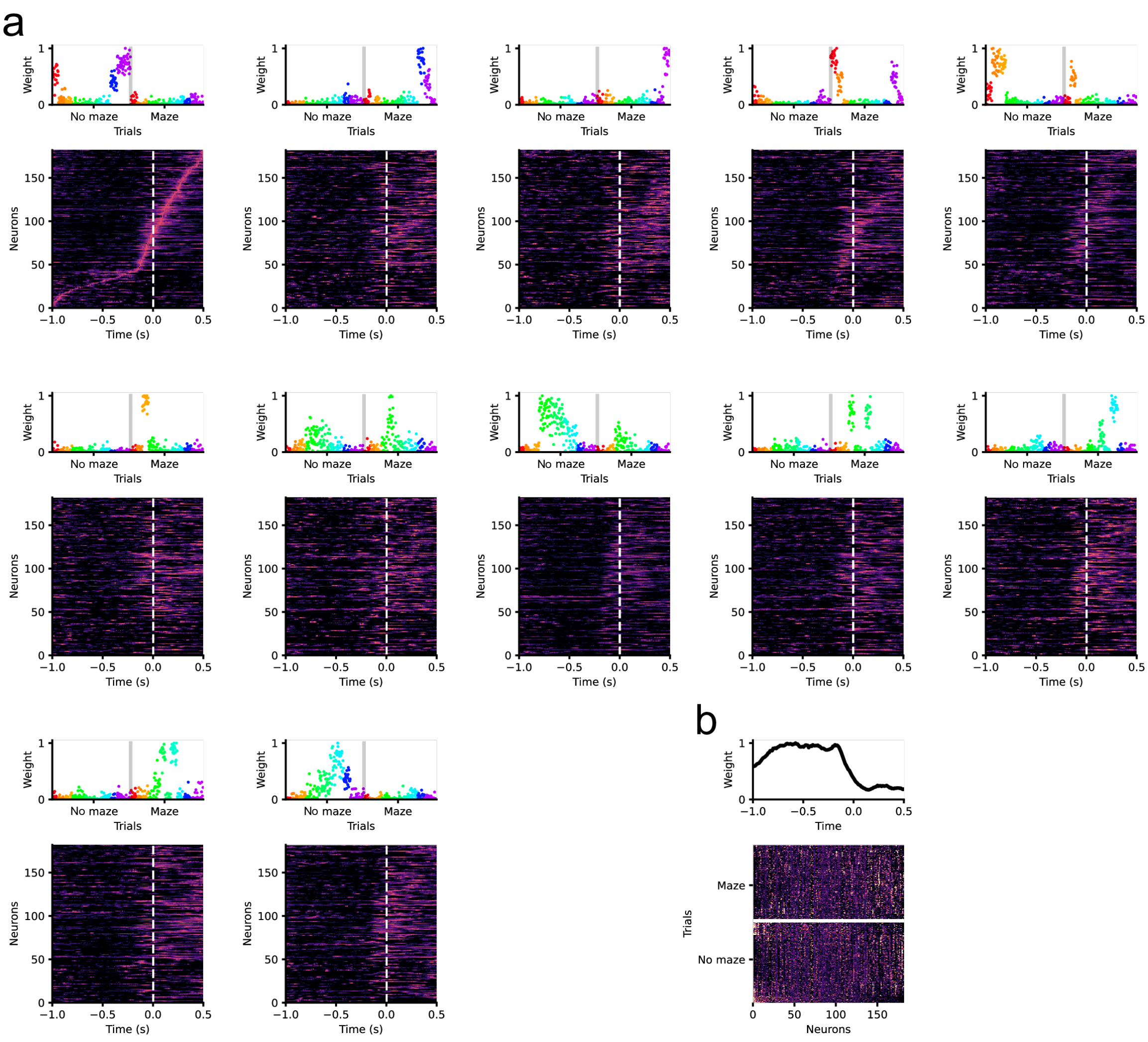

Supplementary Figure 3: Mixed covariability model used for the decoding analysis of reaching kinematics.a. The 12 trial-slicing components of the mixed covariability model. In the slices, neurons are ordered according to latency of peak activation in the first component. These components seem to capture similar reach directions as seen by the loading vector weights having a bell-shaped tuning curve to certain preferred angles. Furthermore, some components seem to capture specifically maze-related neural variability. **b.** The time-slicing component of the mixed covariability model, reproduced from Figure 2d for reference.

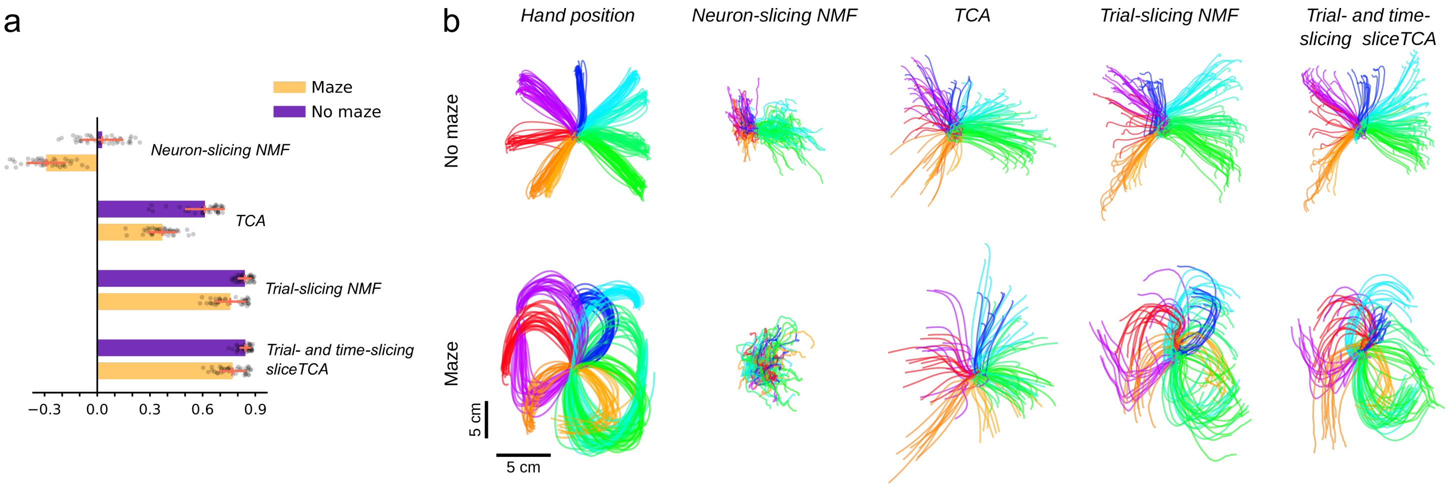

Supplementary Figure 4: Simultaneous dimensionality reduction and decoding cross-validation in the motor cortex reaching dataset. a. We also performed the decoding analysis with a stricter form of cross-validation (cf. Figure 2e). We first split the data in train and test sets with equal number of trials (randomly chosen). We fit the respective dimensionality reduction method to both sets separately, and trained the decoder only on the training dataset. The R^2 reported is that of the decoded hand movement on the test dataset. The operation is repeated by swapping which half is used for fitting and testing (2-fold validation), and repeated over $n = 10$ permutations. We plot the mean R^2 plus/minus the standard deviation over all folds and permutations ($n = 132$ trials for the no maze condition and $n = 123$ trials for the maze condition). Note that in Figure 2, to allow comparison with other methods, we applied the respective dimensionality reduction on the full dataset, before splitting into train- and test-sets for the decoding analysis. Moreover, to avoid overfitting the decoder to the smaller training dataset (compared to Figure 2a), we here use ridge regression for decoding instead of OLS (L_2 regularization coefficient equal to 5 as determined by hyperparameter tuning). **b.** Decoded hand movement on an example test set.

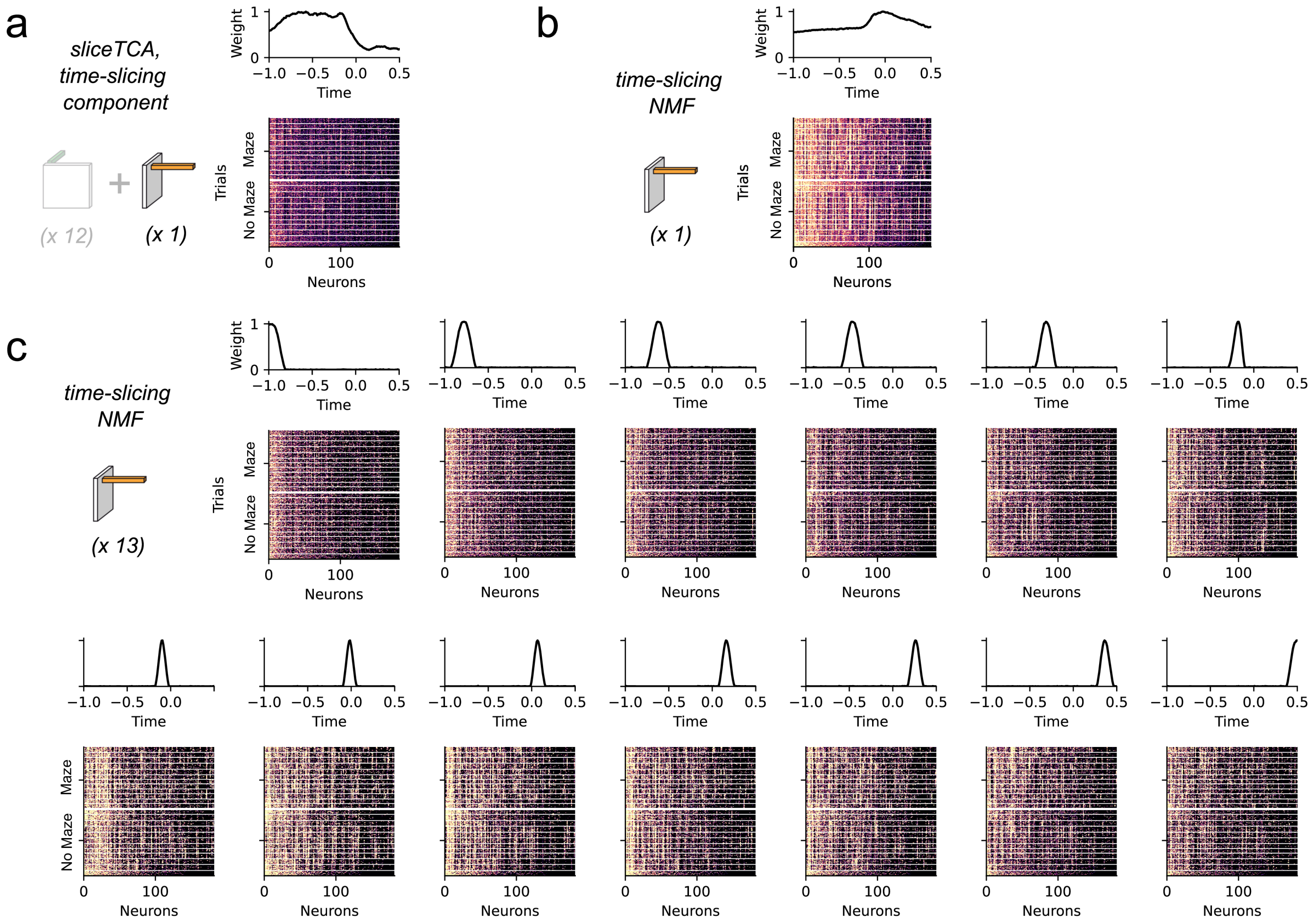

Supplementary Figure 5: Comparison of sliceTCA and time-slicing NMF for reaching dataset. a. SliceTCA time-slicing component ($R_{\text{trial}} = 12$, $R_{\text{time}} = 1$, $R_{\text{neuron}} = 0$) vs. **b,c.** Components of NMF applied to the time-unfolded data tensor for $R_{\text{time}} = 1$ (b) and $R_{\text{time}} = 13$ (c). Neither time-slicing NMF model appears to capture pre-movement preparatory activity.

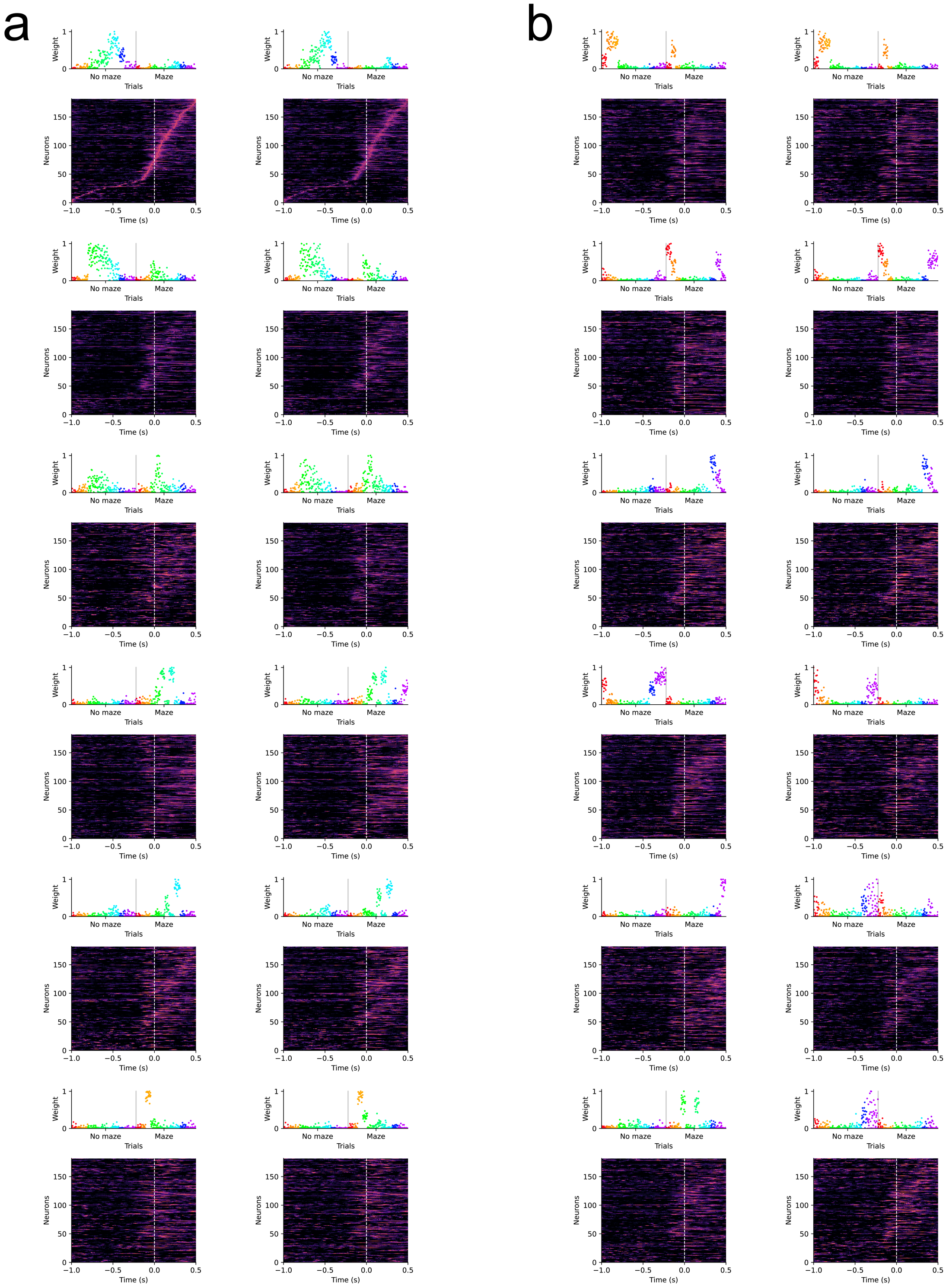

Supplementary Figure 6: Illustration of the lowest-similarity model of the reaching dataset. Trial-slicing components of the best-performing sliceTCA model (**a.,b.**, left column) and its most dissimilar model (slicing-specific model similarity ~ 0.8;**a.,b.**, right column) for the reaching dataset (Figure 2), matched by eye (axes identical to those in Figure 2c). Qualitative differences are observed mainly the last two components.

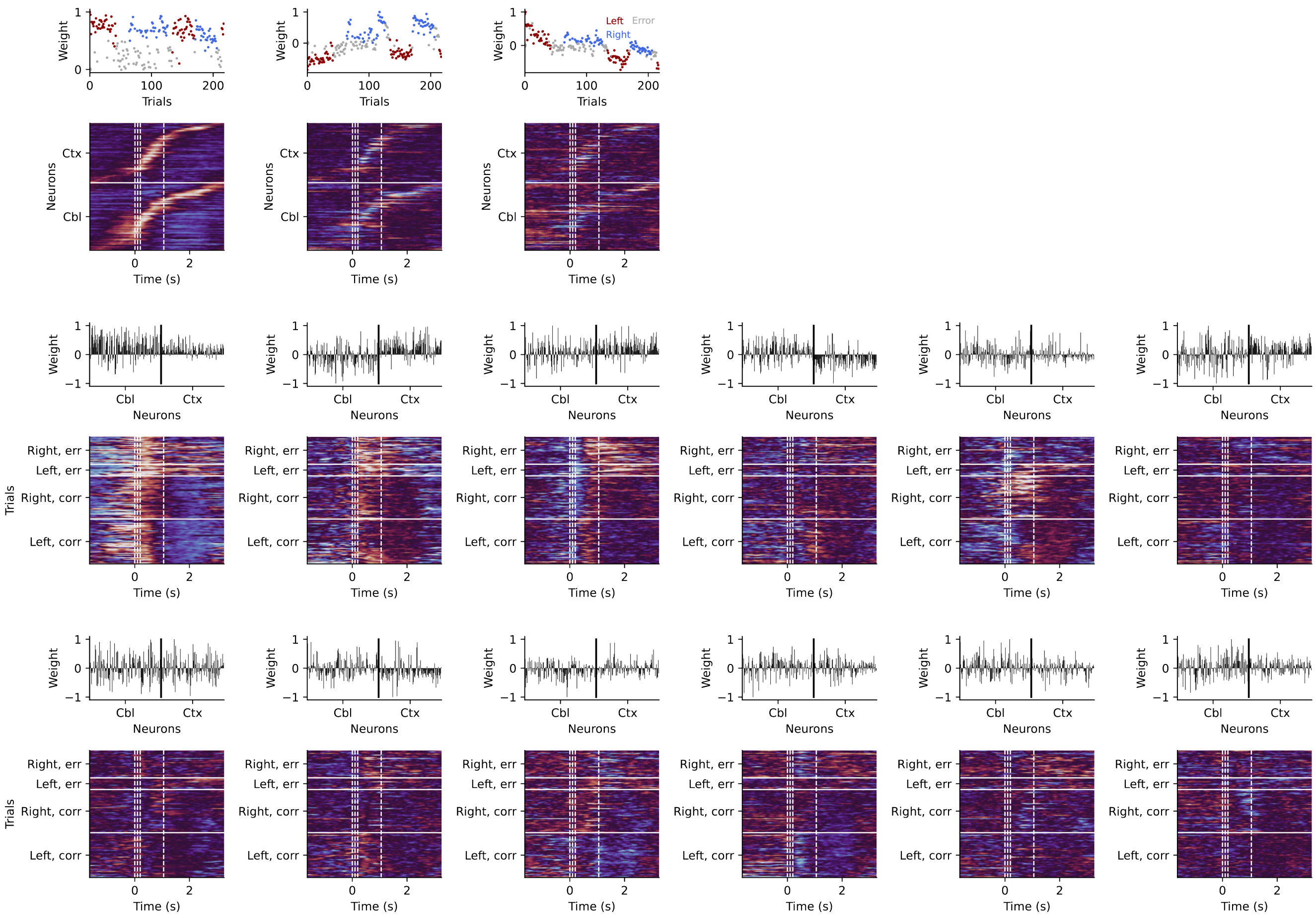

Supplementary Figure 7: Optimal model of cerebello-cortical imaging dataset. The three trial-slicing and twelve neuron-slicing components of the optimal model found from the cross-validated grid search (Extended Data Figure 9a,b; axes identical to the corresponding components displayed in Figure 4b,c). Note that the first three neuron-slicing components, as well as all trial-slicing components, are highly similar to the components in the selected model in Figure 4b,c.

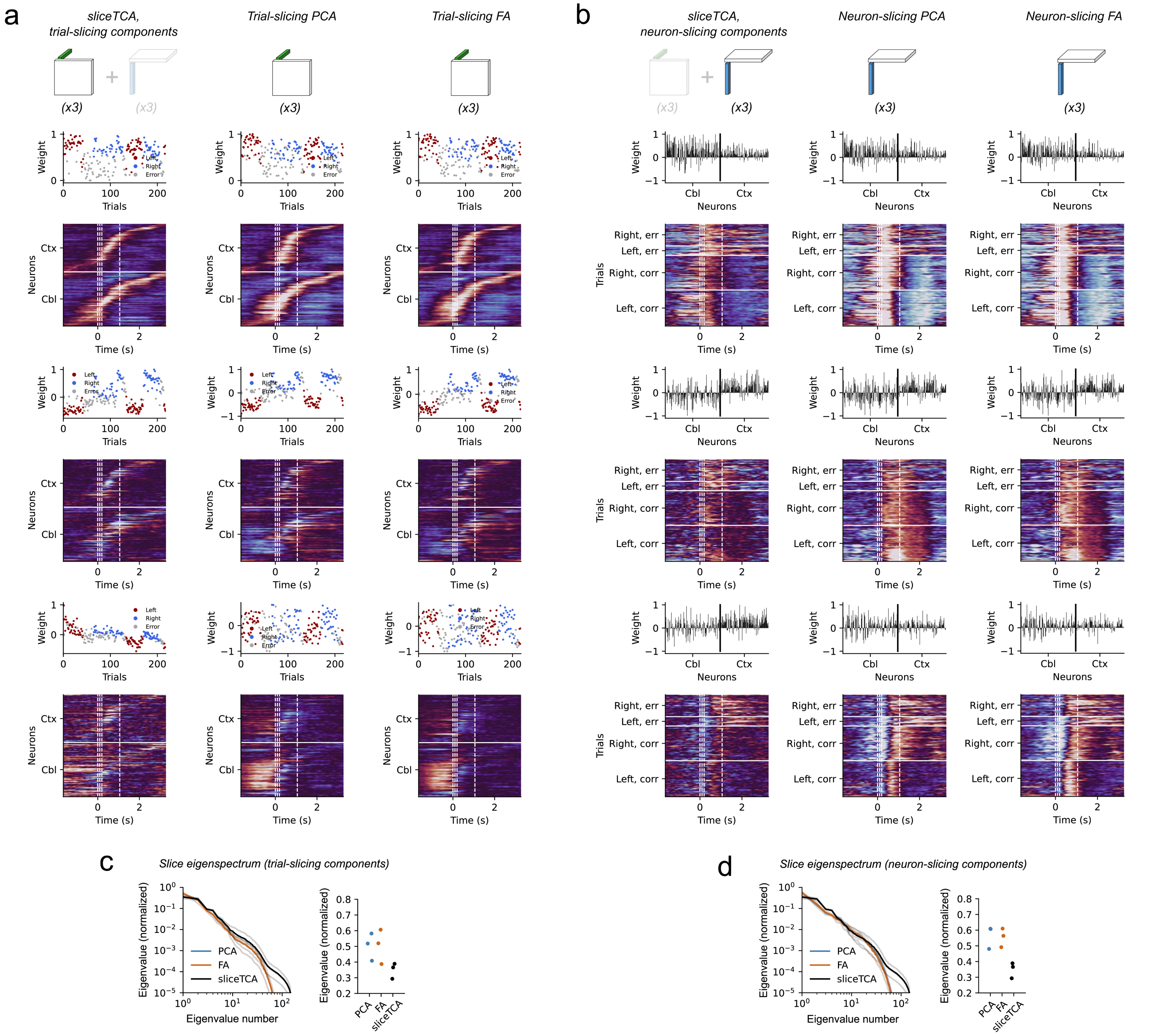

Supplementary Figure 8: PCA and FA components in neuron- and trial-slicing components. a. The three trial-slicing components of the sliceTCA model selected in Figure 4b,c (left), plotted alongside the first three trial-slicing PCA components (middle), and three trial-slicing FA components (right). All slices are ordered by peak neuron activity. Note that the sliceTCA slices appear to be higher rank (as seen by the fact that they appear more diagonally weighted; Figure 4e)). SliceTCA also seems to better capture adaptation-like dynamics in the third component, and better cluster trial types compared to PCA and FA. **b.** Neuron-slicing component of the same sliceTCA decomposition (left), plotted alongside the first three neuron-slicing PCA components (middle), and three neuron-slicing FA components (right). All slices are ordered by trial condition-outcome pairs. Again, the sliceTCA slices appear to be higher rank. **c.** Eigenspectrum analysis for slices of trial-slicing components. Same as Figure 4e. **d.** Eigenspectrum analysis for slices of neuron-slicing components.

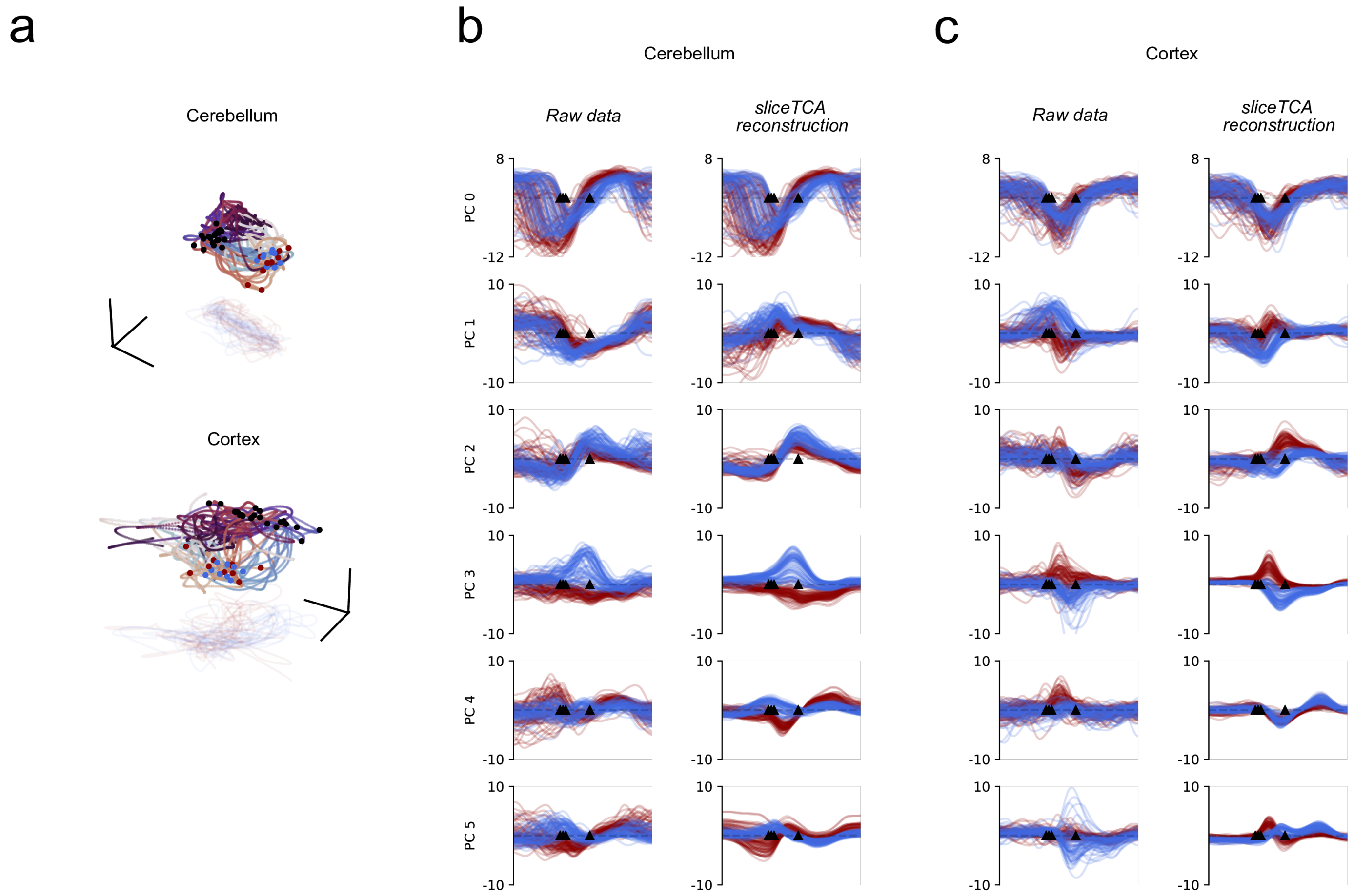

Supplementary Figure 9: Raw data manifolds projected onto LDA axes and principal components in raw data and sliceTCA reconstructions. a. Projection of raw data onto LDA axes that separate left from right trials between movement onset and reward; pre-motor and mid-movement; or movement onset vs. reward time, respectively (cf, Figure 4h; here, LDA axes are found from raw data) **b.** Projection of raw data (left) and sliceTCA reconstructions (right) of cerebellar population activity onto the first six principal components found on raw data (left) and sliceTCA reconstructions (right) for all correct trials. Higher principal components found in sliceTCA reconstructions (from third PC onwards) appear smoother than those found in raw data, and separate trials of different reach direction more clearly. **c.** Same as b for the cortical population.

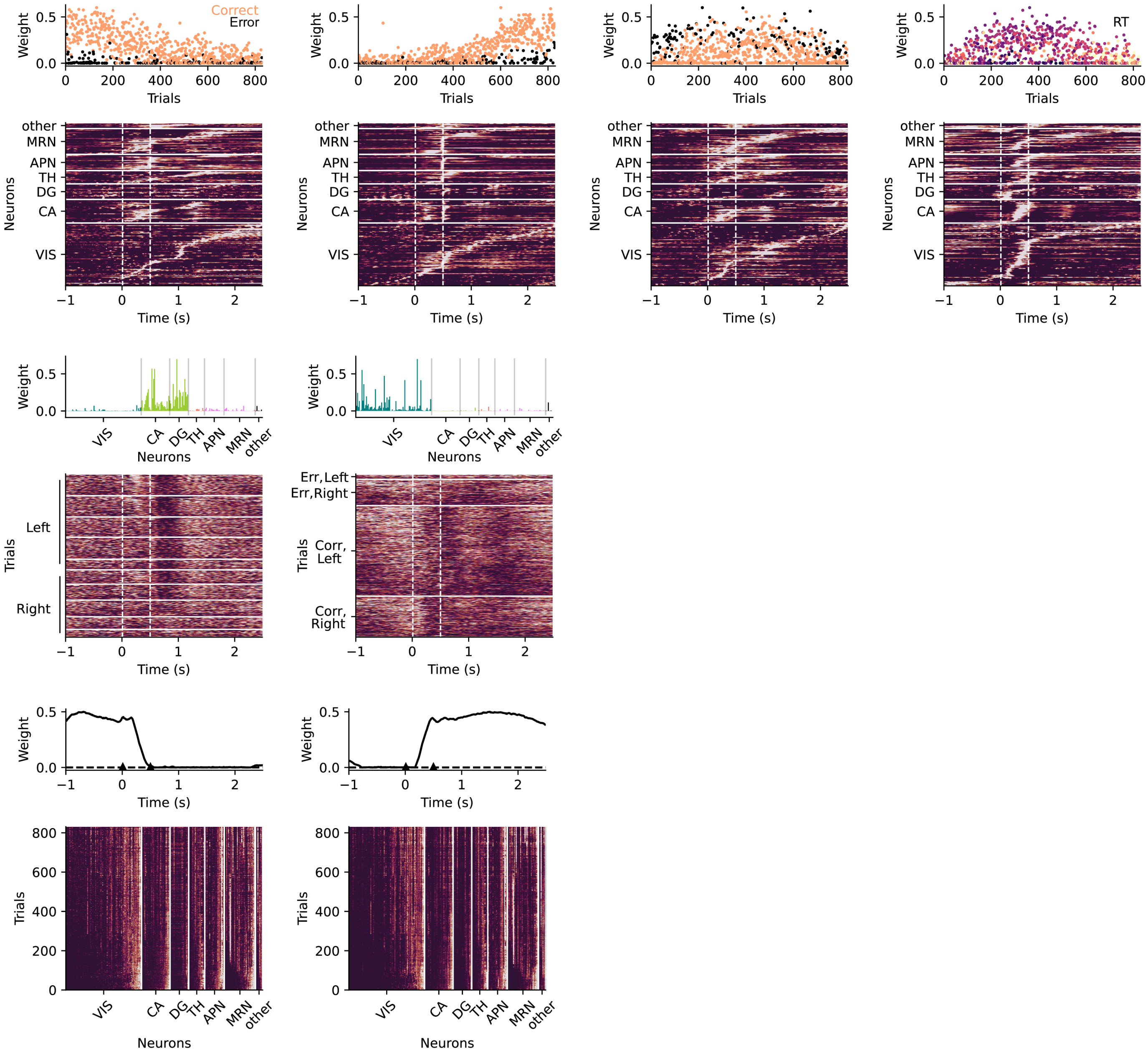

Supplementary Figure 10: Optimal model of multi-region Neuropixel dataset. Components of the optimal model identified by the cross-validated grid search (Extended Data Figure 10a), with 4 trial-slicing components, 2 neuron-slicing and 2 time-slicing components. Similarly to decomposition selected for Figure 5, this model identified time-slicing components related to correct vs. error trials, and a RT-related component. Neural activity related to correct vs. error encoding differed between late and early trials. Moreover, the optimal model grouped CA and DG together in a neuron-slicing component, and lacked a distinction between reward vs. post-reward related time courses in the two time-slicing components.

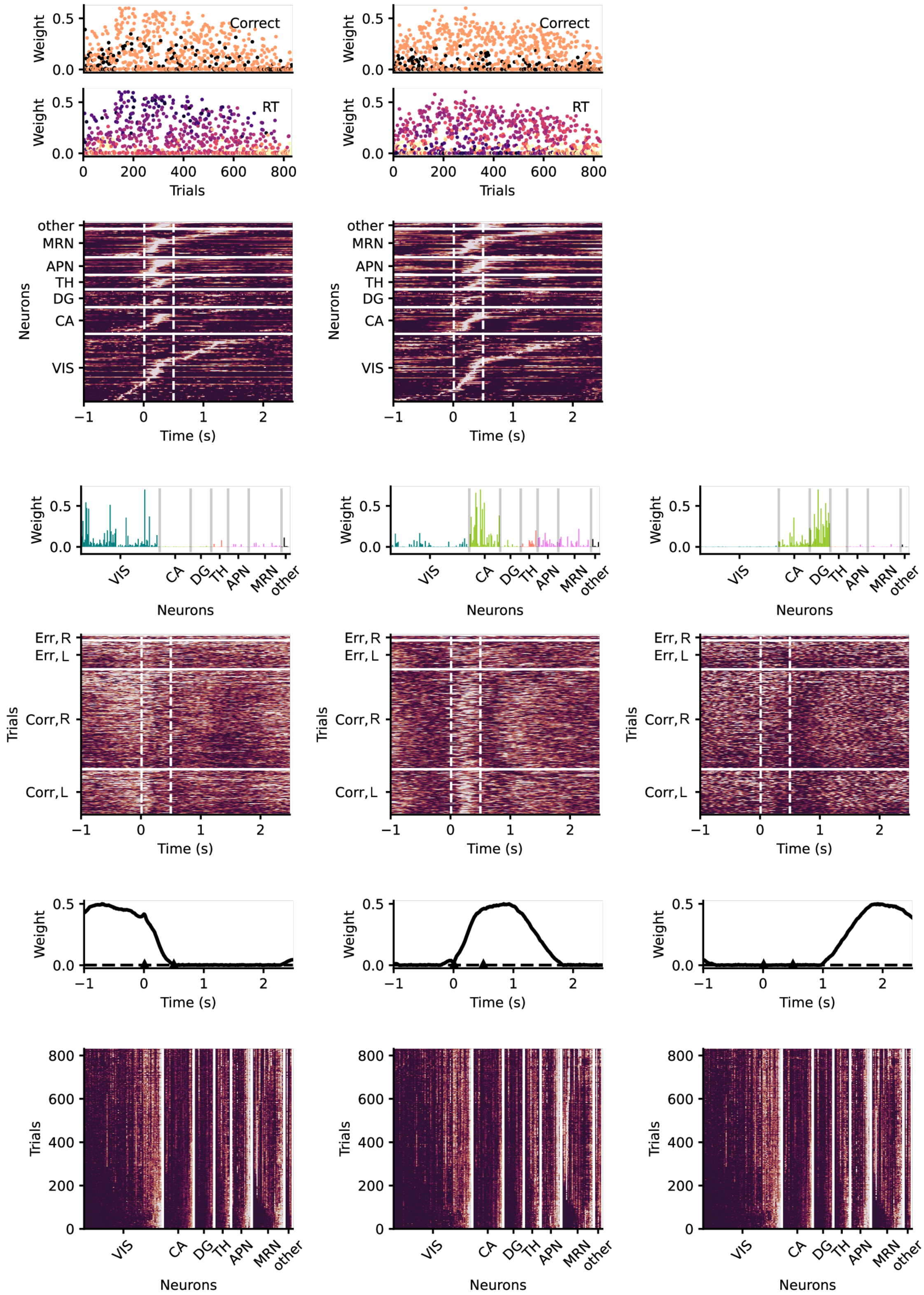

Supplementary Figure 11: SliceTCA on stimulus-onset aligned, but not time-warped, data. SliceTCA on the multi-region Neuropixel dataset without time warping. Instead, trials were trimmed after alignment to form the data tensor. Note that the loading vectors of the trial-slicing components are shown twice, color-coded both by correct vs. error trials (top) and by log reaction time (bottom).

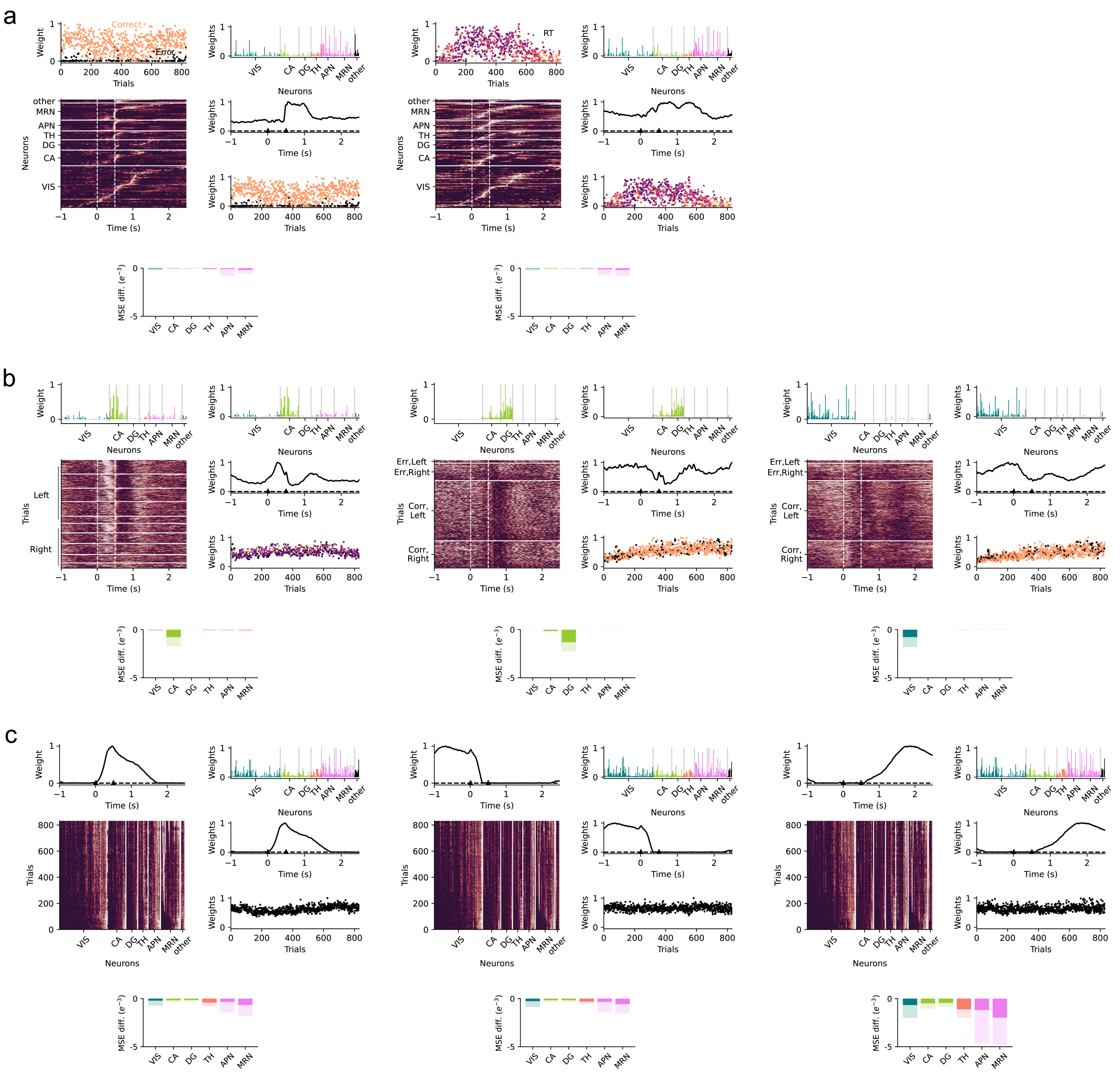

Supplementary Figure 12: Refitting single sliceTCA components as TCA components. a. Trial-slicing, **b.** neuron-slicing, **c.** time-slicing. Weight vectors are color-coded by the most informative behavioral or task variable. In each example, a single TCA component was fitted to the partial reconstruction of a single sliceTCA component. In all panels, sliceTCA components are shown on the left, and the respective refitted TCA component on the right (axes identical to the corresponding components displayed in Figure 5b,f,i). Below each pair of components, average difference in full-model MSE for neurons in different regions when removing a single sliceTCA component (transparent bars) vs. when replacing the respective component with a refitted TCA component (solid bars).

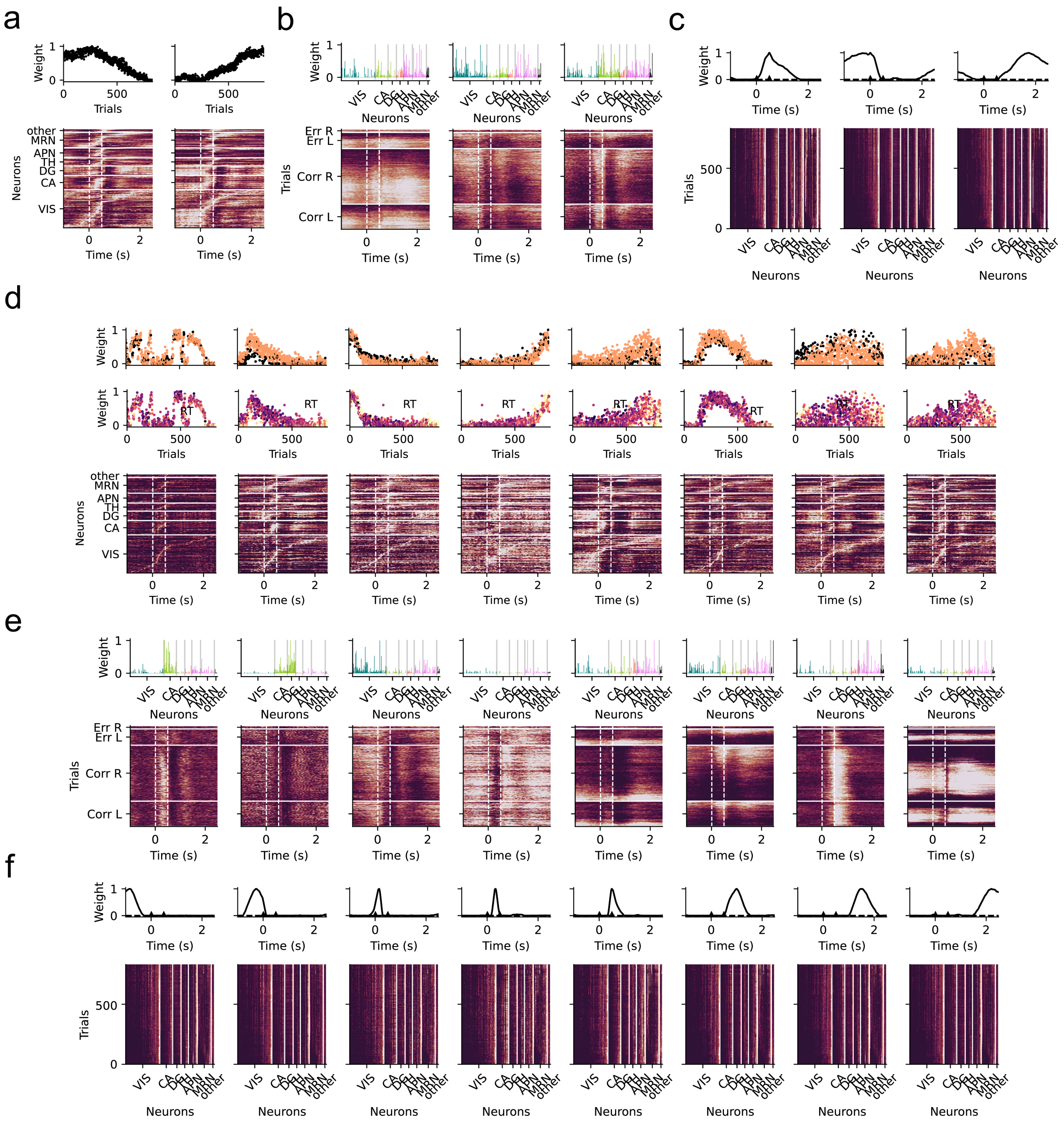

Supplementary Figure 13: Comparison of sliceTCA and NMF on multi-region Neuropixel dataset. a. 2-component trial slicing NMF. **b.** 3-component neuron-slicing NMF. **c.** 3-component time-slicing NMF. **d.** 8-component trial-slicing NMF. **e.** 8-component neuron-slicing NMF. **f.** 8-component time-slicing NMF.

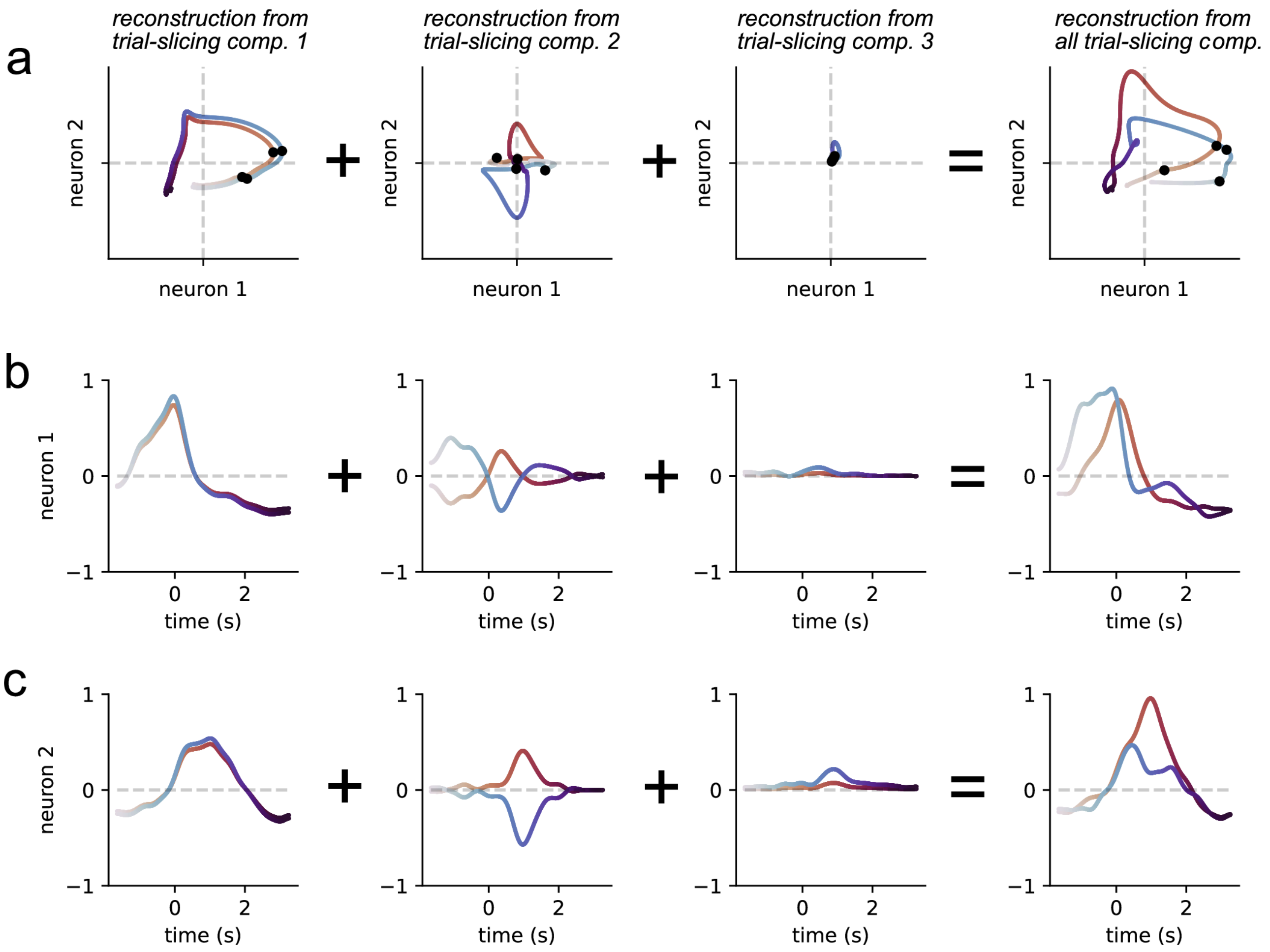

Supplementary Figure 14: Example of reconstruction of multiple trial-slicing components. a. Reconstruction of neural trajectories from three trial-slicing components of the dataset presented in Figure 4. For single components, neural trajectories in the two trials are scaled versions of another. However, the full trial-slicing reconstructions (right) are not simply scaled versions of each other since the three components have different scaling factors. Therefore multiple trial-slicing components can lead to more complex latent dynamics than shown in Figure 6. **b-c.** Single neuron reconstructions plotted against time for each component and the full trial-slicing reconstruction.

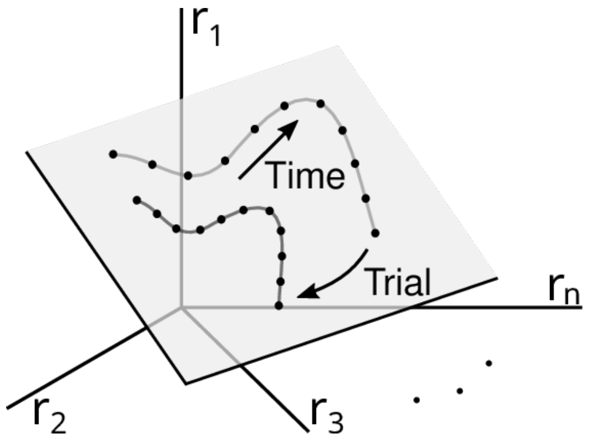

Supplementary Figure 15: Geometric interpretation of TCA components in neural activity space. Similarly to sliceTCA and matrix factorization methods (Figure 6), TCA can be interpreted as uncovering latent variables embedded in neural activity space. Since a TCA component lies at the intersection of the three covariability classes (Figure 1d), it must obey all three constraints described in Figure 6. In other words, the latent dynamics must be embedded in a fixed R-dimensional subspace and can only change in amplitude (not the shape of the dynamics) along the corresponding latent variable across trials.

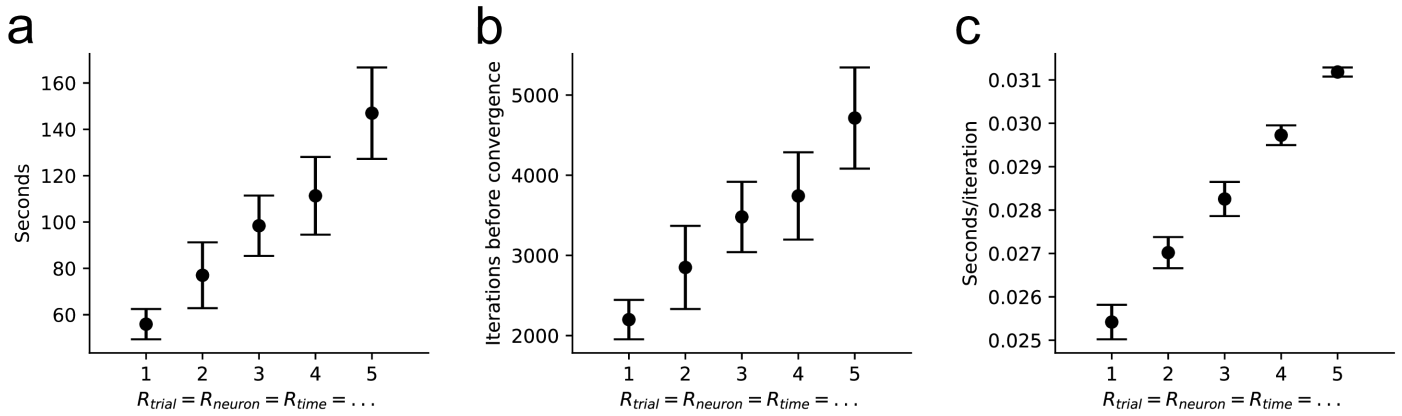

Supplementary Figure 16: SliceTCA run times on a single GPU. To estimate the time required to fit sliceTCA, we computed the run time for a single run on a GPU (NVIDIA A100) in Google Colab, on the motor cortical reaching dataset ($N = 182$ neurons, $K = 511$ trials, and $T = 150$ time points). To compare across models of different sizes, we fitted models with r components of each slice type (i.e., $R_{neuron} = R_{trial} = R_{trial} = r$) for $r = 1, ..., 5$. For each r, we ran the MSE optimization until the convergence criterion was reached (typically after $2000 - 5000$ iterations). We used a learning rate of 10^{-2} and a convergence criterion related to the standard deviation of the loss (a model is considered as converged if the standard deviation of the loss over the last 100 iterations is lower than 10^{-5}). **a.** Average time necessary to fit a single sliceTCA model as a function of the number of components of each slice type, as applied to the motor cortical reaching dataset. Note that since we set all slice types to have the same number of components, the total number of components in the model is threefold the abscissa. **b.** This increase in the run time is the result of an increase in number of iterations needed for the model to converge. **c.** When normalizing run time by the number of iterations, we observe a linear relationship between the number of components per slice type and the time needed for each iteration of optimization. Averages and error bars represent mean $\pm$ 1 standard deviations across $n = 5$ random initializations for panels a-c.

Part II
Mathematical notes on SliceTCA

The theorems presented here complement the results of the main text. In particular, they address the invariance classes of sliceTCA and how to define a unique element in these classes.

1 Definition and motivation

Throughout what follows, a tensor $T \in V_1 \otimes V_2 \otimes V_3$, with decomposition

$$T = \sum_{i=1}^{R_1} [a_i \otimes (bc)_i] + \sum_{i=1}^{R_2} [b_i \otimes (ac)_i] + \sum_{i=1}^{R_3} [c_i \otimes (ab)_i] \tag{1.0.1}$$

is considered[1], where $a_i \in V_1$, $(bc)_i \in V_2 \otimes V_3$, $b_i \in V_2$, $(ac)_i \in V_1 \otimes V_3$, $c_i \in V_3$, $(ab)_i \in V_1 \otimes V_2$, V_i some vector space over an arbitrary field $\mathbb{F}$. Each term of the form $x \otimes (yz)$ is defined as a slice rank 1 tensor [2]. If for all other decompositions of T into a sum of R_1', R_2', R_3' slice rank 1 tensors, $R_1 + R_2 + R_3 \leq R_1' + R_2' + R_3'$, then $R_1 + R_2 + R_3$ is the slice rank of T, and it is said that (1.0.1) is optimal.

Remark 1.1. In this sum, the indices are implicitly permuted to match, so that for example $[a \otimes (bc) + b \otimes (ac)]_{ijk} = T^1_{ijk} + T^2_{jik}$ where $T^1 = a \otimes (bc)$, $T^2 = b \otimes (ac)$ in a formal sense. These permutations are omitted for notational clarity.

As mentioned in the main text, there exists multiple generalizations of the matrix rank to tensors. To illustrate their relationship, we show that the gap between the rank of any unfolding of the tensor and its slice rank can be arbitrarily large. Suppose

$$T = a \otimes (bc) + b \otimes (ac) + c \otimes (ab),$$

where $\text{rank}(bc) = \min(\dim V_2, \dim V_3)$ (that is full rank), $a \neq 0$, and similarly for the other 2-tensors. Then without loss of generality between the slicings, there exists i such that $\text{rank}\, T_i = \text{rank}(a_i(bc) + b \otimes (ac)_i + c \otimes (ab)_i) \geq \min(\dim V_2, \dim V_3) - 2$ as adding a rank 1 matrix decreases the rank by at most 1. Thus, the matrix rank as applied to unfoldings of the tensor can be as large as the minimum of the dimensions of the vector spaces over which the tensor is defined. Furthermore, for $T = a \otimes (bc)$, $\text{rank}(bc) = \min(\dim V_2, \dim V_3)$, the tensor rank is exactly $\min(\dim V_2, \dim V_3)$ while the matrix rank as applied to the appropriate unfolding is 1, and thus the gap is also proportional to the dimension of the shortest leg of the tensor. Therefore, there exists a gap between the slice rank and the matrix rank on any unfolding, and the slice rank and the tensor rank.

To help the reader, properties of tensors that are used extensively in the upcoming proofs are stated. Here $a, a' \in V_1$, $b \in V_2$, $c \in V_3$, $r \in \mathbb{F}$, where V_i is a vector space over the field $\mathbb{F}$.

1. Multilinear addition. $(a + a') \otimes b \otimes c = a \otimes b \otimes c + a' \otimes b \otimes c$.
2. Multilinear scalar multiplication. $ra \otimes b \otimes c = a \otimes rb \otimes c = a \otimes b \otimes rc$.
3. Tensor product. Define $V_1 \otimes V_2 = \text{span}\{e_i \otimes e_j\}$ where $\{e_i\}, \{e_j\}$ form bases of V_1 and V_2 respectively. The first "$\otimes$" in the expression refers to a tensor product, the second to an outer product. The tensor product is a vector space itself, thus $V_1 \otimes V_2 \otimes V_3 = V_1 \otimes (V_2 \otimes V_3)$.
4. Disjoint spaces. If $V_1 \cap V_1' = \{0\}$ then $V_1 \otimes V_2 \cap V_1' \otimes V_2 = \{0\}$.

2 Invariances and uniqueness of decompositions

The main result of this section is that under the weak condition of optimality of the decomposition and a condition on the rank of the slices, the set of slice rank decompositions of a tensor can be fully characterized by that decomposition and two natural transformations on this decomposition.

Two transformations on decomposition (1.0.1) are defined.

[1] We introduce the notation $(bc)_i, (ac)_i, (ab)_i$ to denote arbitrary 2-tensors.

1. The within-slicing transformation,

$$\sum_{i=1}^{R_1} a_i \otimes (bc)_i = \sum_{i=1}^{R_1} \left(\sum_{j=1}^{R_1} X_{ji} a_j\right) \otimes \left(\sum_{j=1}^{R_1} X_{ij}^{-1} (bc)_j\right)$$

for $X \in GL(R_1)$, and similarly for the other slicings.

2. The between-slicings transformation,

$$\sum_{i=1}^{R_1} a_i \otimes (bc)_i + \sum_{i=1}^{R_2} b_i \otimes (ac)_i = \sum_{i=1}^{R_1} a_i \otimes \left[(bc)_i + \sum_{j=1}^{R_2} b_j \otimes z_{ij}\right] + \sum_{i=1}^{R_2} b_i \otimes \left[(ac)_i - \sum_{j=1}^{R_1} a_j \otimes z_{ji}\right]$$

for $z_{ij} \in V_3$, and similarly for the other pairs of slicings.

Proposition 2.1. *These two transformations commute up to the z_{ij}.*

Proof.

$$\begin{aligned}
&\sum_{i=1}^{R_1} \left(\sum_{j=1}^{R_1} X_{ji} a_j\right) \otimes \left[\left(\sum_{j=1}^{R_1} X_{ij}^{-1} (bc)_j\right) + \sum_{k=1}^{R_2} b_k \otimes z_{ik}\right] + \sum_{i=1}^{R_2} b_i \otimes \left[(ac)_i - \sum_{k=1}^{R_1} \left(\sum_{j=1}^{R_1} X_{jk} a_j\right) \otimes z_{ki}\right] \\
=&\sum_{i=1}^{R_1} \left(\sum_{j=1}^{R_1} X_{ji} a_j\right) \otimes \left[\sum_{j=1}^{R_1} X_{ij}^{-1} \left((bc)_j + \sum_{k=1}^{R_2} b_k \otimes X_{ji} z_{ik}\right)\right] + \sum_{i=1}^{R_2} b_i \otimes \left[(ac)_i - \sum_{j=1}^{R_1} a_j \otimes \left(\sum_{k=1}^{R_1} X_{jk} z_{ki}\right)\right].
\end{aligned}$$

□

Thus, from (1.0.1) a class of decompositions can be naturally defined. Let T be defined as in (1.0.1). Then,

$$\begin{aligned}
T =& \sum_{i=1}^{R_1} \left[\left(\sum_{j=1}^{R_1} X_{ji} a_j\right) \otimes \left(\sum_{j=1}^{R_1} X_{ij}^{-1} (bc)_j\right) + \sum_{j=1}^{R_2} a_i \otimes b_j \otimes z_{ij} + \sum_{j=1}^{R_3} a_i \otimes y_{ij} \otimes c_j\right] \\
&+ \sum_{i=1}^{R_2} \left[\left(\sum_{j=1}^{R_2} Y_{ji} b_j\right) \otimes \left(\sum_{j=1}^{R_2} Y_{ij}^{-1} (ac)_j\right) - \sum_{j=1}^{R_1} a_j \otimes b_i \otimes z_{ji} + \sum_{j=1}^{R_3} x_{ij} \otimes b_i \otimes c_j\right] \\
&+ \sum_{i=1}^{R_3} \left[\left(\sum_{j=1}^{R_3} Z_{ji} c_j\right) \otimes \left(\sum_{j=1}^{R_3} Z_{ij}^{-1} (ab)_j\right) - \sum_{j=1}^{R_1} a_j \otimes y_{ji} \otimes c_i - \sum_{j=1}^{R_2} x_{ji} \otimes b_j \otimes c_i\right].
\end{aligned}$$

The main purpose of these notes is to show that this class contains *all* optimal slice rank decompositions of T under some weak assumptions.

2.1 Properties of the slice rank

It is first shown that factors of any decomposition of fixed slice rank tuple of T must lie in the same subspace.

Theorem 2.2. *Let $T \in V_1 \otimes V_2 \otimes V_3$ such that*

$$T = \sum_{i=1}^{R_1} [a_i \otimes (bc)_i] + \sum_{i=1}^{R_2} [b_i \otimes (ac)_i] + \sum_{i=1}^{R_3} [c_i \otimes (ab)_i].$$

Suppose there exists another decomposition of T,

$$T = \sum_{i=1}^{R_1} [u_i \otimes (vw)_i] + \sum_{i=1}^{R_2} [v_i \otimes (uw)_i] + \sum_{i=1}^{R_3} [w_i \otimes (uv)_i].$$

Then $u_i \in V_1' = \operatorname{span}\{a_i\} \cup \{(uw)_{i,j}\} \cup \{(uv)_{i,j}\}$, and $v_i \in V_2'$, $w_i \in V_3'$, constructed similarly to V_1'. Furthermore, $(vw)_i \in V_2' \otimes V_3'$, $(uw)_i \in V_1' \otimes V_3'$, $(uv)_i \in V_1' \otimes V_2'$.

Proof. The proof follows a similar scheme as the tensor rank decomposition case [1]. Let $u_i' + u_i'' = u_i$ such that $u_i \in V_1' = \text{span}\{a_i\} \cup \{(uw)_{i,j}\} \cup \{(uv)_{i,j}\}$ (the second index is over the V_1 space), $u_i'' \in V_1'' = V_1 \setminus V_1'$, that is $V_1' \oplus V_1'' = V_1$. Similarly, let $v_i' \in V_2'$, $v_i'' \in V_2''$, $V_2' \oplus V_2'' = V_2$, $w_i' \in V_3'$, $w_i'' \in V_3''$, $V_3' \oplus V_3'' = V_3$. Furthermore, let $(vw)_i' \in V_2' \otimes V_3'$, $(vw)_i'' \in V_2'' \otimes V_3' \cup V_2' \otimes V_3'' \cup V_2'' \otimes V_3''$ and similarly for $(uw)_i$ and $(uv)_i$. Since $V_i = V_i' \oplus V_i''$, $V_2 \otimes V_3 = (V_2' \otimes V_3') \oplus (V_2'' \otimes V_3') \oplus (V_2' \otimes V_3'') \oplus (V_2'' \otimes V_3'')$ and similarly for the other terms, the decomposition can be rewritten as,

$$\begin{aligned} T = &\sum_{i=1}^{R_1}[(u_i' + u_i'') \otimes ((vw)_i' + (vw)_i'')] + \sum_{i=1}^{R_2}[(v_i' + v_i'') \otimes ((uw)_i' + (uw)_i'')] && (2.1.1)\\ &+ \sum_{i=1}^{R_3}[(w_i' + w_i'') \otimes ((uv)_i' + (uv)_i'')], && (2.1.2) \end{aligned}$$

that is,

$$\begin{aligned} T = &\sum_{i=1}^{R_1}[u_i' \otimes (vw)_i' + u_i' \otimes (vw)_i'' + u_i'' \otimes (vw)_i' + u_i'' \otimes (vw)_i''] && (2.1.3)\\ &+ \sum_{i=1}^{R_2}[v_i' \otimes (uw)_i' + v_i' \otimes (uw)_i'' + v_i'' \otimes (uw)_i' + v_i'' \otimes (uw)_i''] && (2.1.4)\\ &+ \sum_{i=1}^{R_3}[w_i' \otimes (uv)_i' + w_i' \otimes (uv)_i'' + w_i'' \otimes (uv)_i' + w_i'' \otimes (uv)_i'']. && (2.1.5) \end{aligned}$$

But by equation (1.0.1), $T \in V_1' \otimes V_2' \otimes V_3'$. Since all other terms are in subspaces disjoint from $V_1' \otimes V_2' \otimes V_3'$, it follows that $u_i' \otimes (vw)_i'' = v_i'' \otimes (uw)_i' = ... = 0$. That is,

$$T = \sum_{i=1}^{R_1} u_i' \otimes (vw)_i' + \sum_{i=1}^{R_2} v_i' \otimes (uw)_i' + \sum_{i=1}^{R_3} w_i' \otimes (uv)_i'. \qquad (2.1.6)$$

□

This theorem is useful as it tells that no additional consideration of spans of spaces over which the tensor is defined have to be taken into consideration when going from tensor rank to tensor slice rank decompositions.

Proposition 2.3. *Let T be decomposed optimally as in* (1.0.1). *Then without loss of generality between the slicings* $\text{span}\{(bc)_i\} \cap (\text{span}\{b_i\} \otimes V_3 \cup V_2 \otimes \text{span}\{c_i\}) = \{0\}$.

Proof. Suppose there exists $\{q_i\}$ such that

$$\sum_{i=1}^{R_1} q_i (bc)_i = \sum_{i=1}^{R_2} b_i \otimes z_i + \sum_{i=1}^{R_3} y_i \otimes c_i$$

for $z_i \in V_3, y_i \in V_2$. Consider some $M \in GL(R_1)$ such that $M_1 = (q_i)$ and apply it and its inverse to $(bc)_i$ and a_i, respectively, to get $(bc)_i'$ and a_i' (i.e., the within-slicings transformation). Then,

$$\begin{aligned} T &= a_1' \otimes \sum_{i=1}^{R_1} q_i (bc)_i + \sum_{i=2}^{R_1}[a_i' \otimes (bc)_i'] + \sum_{i=1}^{R_2}[b_i \otimes (ac)_i] + \sum_{i=1}^{R_3}[c_i \otimes (ab)_i]\\ &= a_1' \otimes \sum_{i=1}^{R_2} b_i \otimes z_i + a_1' \otimes \sum_{i=1}^{R_3} y_i \otimes c_i + \sum_{i=2}^{R_1}[a_i' \otimes (bc)_i'] + \sum_{i=1}^{R_2}[b_i \otimes (ac)_i] + \sum_{i=1}^{R_3}[c_i \otimes (ab)_i]\\ &= \sum_{i=1}^{R_2} a_1' \otimes b_i \otimes z_i + \sum_{i=1}^{R_3} a_1' \otimes y_i \otimes c_i + \sum_{i=2}^{R_1}[a_i' \otimes (bc)_i'] + \sum_{i=1}^{R_2}[b_i \otimes (ac)_i] + \sum_{i=1}^{R_3}[c_i \otimes (ab)_i]\\ &= \sum_{i=2}^{R_1}[a_i' \otimes (bc)_i'] + \sum_{i=1}^{R_2}[b_i \otimes ((ac)_i + a_1' \otimes z_i)] + \sum_{i=1}^{R_3}[c_i \otimes ((ab)_i + a_1' \otimes y_i)]. \end{aligned}$$

That is, T is of slice rank $R_1 + R_2 + R_3 - 1$ which is a contradiction since (1.0.1) was assumed to be optimal. □

The proof of the above proposition essentially uses the fact that the transformations cannot get rid of a term if the decomposition is optimal.

Proposition 2.4. *Let T as in* (1.0.1). *Then there exists*

$$T = \sum_{i=1}^{R_1}[a_i \otimes (bc)'_i] + \sum_{i=1}^{R_2}[b_i \otimes (ac)'_i] + \sum_{i=1}^{R_3}[c_i \otimes (ab)'_i]$$

such that $(bc)'_{i,j} \notin \mathrm{span}\{b_i\}$ and $(bc)'_{i,\cdot,j} \notin \mathrm{span}\{c_i\}$, and similarly (but not necessarily simultaneously) for the other slicings.

Proof. For all j such that $(bc)_{i,j} \in \mathrm{span}\{b_j\}$ let $x \in \mathbb{F}^{R_1} \otimes \mathbb{F}^{R_3} \otimes \mathbb{F}^{R_2}$ such that

$$(bc)_{i,j} = \sum_{k=1}^{R_2} x_{ijk} b_k$$

and $x_{ijk} = 0$ for all other j. Now let

$$(bc)'_{i,j} = (bc)_{i,j} - \sum_{k=1}^{R_2} x_{ijk} b_k.$$

Then $(bc)'_i \in V_2 \backslash \mathrm{span}\{b_i\} \otimes V_3$. The same step can be taken with respect to V_3 such that $(bc)'_i \in V_2 \backslash \mathrm{span}\{b_i\} \otimes V_3 \backslash \mathrm{span}\{c_i\}$. □

This proposition shows that terms of different slicings of the decomposition can be made to lie in disjoint spaces using the transformations.

Proposition 2.5. *Let T as in* (1.0.1). *Then there exists*

$$T = \sum_{i=1}^{R_1}[a_i \otimes (bc)'_i] + \sum_{i=1}^{R_2}[b_i \otimes (ac)'_i] + \sum_{i=1}^{R_3}[c_i \otimes (ab)'_i]$$

such that $(bc)'_{i,j} \notin \mathrm{span}\{b_i\}$ and $(ab)'_{i,\cdot,j} \notin \mathrm{span}\{b_i\}$, and similarly (but not necessarily simultaneously) for the other slicings.

Proof. The same step as above can be taken so that $(bc)'_i \in V_2 \setminus (\mathrm{span}\{b_i\} \otimes V_3)$ and $(ab)'_i \in V_1 \otimes (V_2 \setminus \mathrm{span}\{b_i\})$. □

2.2 Main theorem

Lemma 2.6. *Let T be decomposed optimally as in 1.0.1. If $\mathrm{rank}(bc)'_i > 2(R_2 + R_3)$ for all i and for all within-slicing transformations, and similarly for the other slicings, then $\mathrm{span}\{u_i\} = \mathrm{span}\{a_i\}$, $\mathrm{span}\{v_i\} = \mathrm{span}\{b_i\}$, $\mathrm{span}\{w_i\} = \mathrm{span}\{c_i\}$.*

Proof. Suppose there exists another decomposition,

$$T = \sum_{i=1}^{R_1}[u_i \otimes (vw)_i] + \sum_{i=1}^{R_2}[v_i \otimes (uw)_i] + \sum_{i=1}^{R_3}[w_i \otimes (uv)_i].$$

Then there exists $P_1, P_2 \subseteq [R_1]$ such that $\mathrm{span}\{u_i\} = \mathrm{span}\{a_j\}, i \in P_1, j \in P_2$. Therefore, there exist $(bc)'_i$ such that

$$\begin{aligned} 0 = &\sum_{i \in P_1}[u_i \otimes ((vw)_i - (bc)'_i)] + \sum_{i \in [R_1] \backslash P_1}[u_i \otimes (vw)_i] \\ &+ \sum_{i=1}^{R_2}[v_i \otimes (uw)_i] + \sum_{i=1}^{R_3}[w_i \otimes (uv)_i] \\ &- \sum_{i \in [R_1] \backslash P_2}[a_i \otimes (bc)_i] - \sum_{i=1}^{R_2}[b_i \otimes (ac)_i] - \sum_{i=1}^{R_3}[c_i \otimes (ab)_i]. \end{aligned}$$

By applying proposition 2.4 to the whole tensor, let $(bc)'_i \in V_2 \otimes V_3 \setminus (\mathrm{span}\{b_j\} \otimes V_3 \cup \mathrm{span}\{v_j\} \otimes V_3 \cup V_2 \otimes \mathrm{span}\{c_j\} \cup V_2 \otimes \mathrm{span}\{w_j\})$ for all i. Then,

$$0 = \sum_{i \in P_1}[u_i \otimes ((vw)_i - (bc)'_i)] + \sum_{i \in [R_1] \backslash P_1}[u_i \otimes (vw)_i]$$

$$+\sum_{i=1}^{R_2}[v_i \otimes (uw)'_i] + \sum_{i=1}^{R_3}[w_i \otimes (uv)'_i]$$
$$-\sum_{i\in[R_1]\setminus P_2}[a_i \otimes (bc)'_i] - \sum_{i=1}^{R_2}[b_i \otimes (ac)'_i] - \sum_{i=1}^{R_3}[c_i \otimes (ab)'_i].$$

By the assumption that $\text{rank}(bc)_i > 2(R_2 + R_3), (bc)'_i \neq 0$. This is a contradiction as it is independent of all other terms, therefore $P_2 = [R_1]$, that is $\text{span}\{u_i\} = \text{span}\{a_i\}$. By a similar argument, $\text{span}\{v_i\} = \text{span}\{b_i\}$, $\text{span}\{w_i\} = \text{span}\{c_i\}$.

□

Theorem 2.7. *Let $T \in V_1 \otimes V_2 \otimes V_3$ such that*

$$T = \sum_{i=1}^{R_1}[a_i \otimes (bc)_i] + \sum_{i=1}^{R_2}[b_i \otimes (ac)_i] + \sum_{i=1}^{R_3}[c_i \otimes (ab)_i]$$

and

$$T = \sum_{i=1}^{R_1}[u_i \otimes (vw)_i] + \sum_{i=1}^{R_2}[v_i \otimes (uw)_i] + \sum_{i=1}^{R_3}[w_i \otimes (uv)_i]$$

are two optimal decompositions of T. Furthermore, suppose $\text{span}\{a_i\} = \text{span}\{u_i\}$, $\text{span}\{b_i\} = \text{span}\{v_i\}$, $\text{span}\{c_i\} = \text{span}\{w_i\}$. Then the two decompositions are equivalent up to transformations.

Proof. By the transformations,

$$0 = \sum_{i=1}^{R_1}[a_i \otimes ((vw)'_i - (bc)_i)] + \sum_{i=1}^{R_2}[b_i \otimes ((uw)'_i - (ac)_i)] + \sum_{i=1}^{R_3}[c_i \otimes ((uv)'_i - (ab)_i)]$$
$$+ \sum_{ij} x_{ij} \otimes b_i \otimes c_j + \sum_{ij} a_i \otimes y_{ij} \otimes c_j + \sum_{ij} a_i \otimes b_j \otimes z_{ij}.$$

So, without loss of generality, $a_i \otimes ((vw)'_i - (bc)_i) = 0$, that is $(vw)'_i = (bc)_i$ as it is independent of all other terms. □

3 Convexity of transformations

The results of the previous section hint at looking for sufficient criteria for uniqueness of the two transformations. Here it is shown that convex objective functions applied to appropriate tensors of the decomposition will provide such uniquenesses.

Let $L : \mathbb{R} \to \mathbb{R}$ a strictly convex function. Let T^1, T^2, T^3 the reconstructed tensors of each type. The regularization loss can be written as,

$$l(\alpha, \beta, \gamma) = \sum_{ijk} L\left(T^1_{ijk} - \sum_{m,n} \alpha_i^{m,n} b_j^n c_k^m - \sum_{m,n} a_i^m \beta_j^{m,n} c_k^n\right)$$
$$+ \sum_{ijk} L\left(T^2_{ijk} + \sum_{m,n} \alpha_i^{m,n} b_j^n c_k^m - \sum_{m,n} a_i^m b_j^n \gamma_k^{m,n}\right)$$
$$+ \sum_{ijk} L\left(T^3_{ijk} + \sum_{m,n} a_i^m \beta_j^{m,n} c_k^n + \sum_{m,n} a_i^m b_j^n \gamma_k^{m,n}\right) \tag{3.0.1}$$

The following well-known properties of convex functions are used to prove the strict convexity of l.

Lemma 3.1. *Let $f, g : \mathbb{R}^n \to \mathbb{R}$ be strictly convex, and $A \in \mathbb{R}^{m,n}$, $b \in \mathbb{R}^n$, then,*

1. *$f(Ax + b)$ is strictly convex.*
2. *$(f + g) : \mathbb{R}^n \to \mathbb{R}$ is strictly convex.*

3. f admits a unique minimum on a $\mathbb{R}^n$.

By 1. and 2. of lemma 3.1, since L is strictly convex l is strictly convex.

Lemma 3.2. *Let $f : \mathbb{R}^n \to \mathbb{R}$ convex. Then stochastic gradient descent with appropriately decreasing learning rate converges.*

Therefore, by 3. of lemma 3.1, the optimization procedure of regularization under a strictly convex function (e.g. L2 regularization) converges to a unique criterion.

References

[1] J. M. Landsberg. Kruskal's theorem, 2009.

[2] T. Tao and W. Sawin. Notes on the "slice rank" of tensors, 08 2016.
