# Supplementary material for: Dimensionality reduction beyond neural subspaces with slice tensor component analysis
Source: Nat Neurosci. 2024 May 6;27(6):1199–210. doi: 10.1038/s41593-024-01626-2 (PMC11537991; doi:10.1038/s41593-024-01626-2)
Supplement: Supplementary file 2 — Reporting Summary [file 41593_2024_1626_MOESM2_ESM.pdf]

nature portfolio

Corresponding author(s): N Alex Cayco Gajic, Arthur Pellegrino

Last updated by author(s): Jan 16, 2024

Reporting Summary

Nature Portfolio wishes to improve the reproducibility of the work that we publish. This form provides structure for consistency and transparency in reporting. For further information on Nature Portfolio policies, see our Editorial Policies and the Editorial Policy Checklist.

Statistics

For all statistical analyses, confirm that the following items are present in the figure legend, table legend, main text, or Methods section.

n/a	Confirmed	
☐	☒	The exact sample size (*n*) for each experimental group/condition, given as a discrete number and unit of measurement
☐	☒	A statement on whether measurements were taken from distinct samples or whether the same sample was measured repeatedly
☐	☒	The statistical test(s) used AND whether they are one- or two-sided<br>*Only common tests should be described solely by name; describe more complex techniques in the Methods section.*
☒	☐	A description of all covariates tested
☐	☒	A description of any assumptions or corrections, such as tests of normality and adjustment for multiple comparisons
☐	☒	A full description of the statistical parameters including central tendency (e.g. means) or other basic estimates (e.g. regression coefficient) AND variation (e.g. standard deviation) or associated estimates of uncertainty (e.g. confidence intervals)
☐	☒	For null hypothesis testing, the test statistic (e.g. *F*, *t*, *r*) with confidence intervals, effect sizes, degrees of freedom and *P* value noted<br>*Give P values as exact values whenever suitable.*
☒	☐	For Bayesian analysis, information on the choice of priors and Markov chain Monte Carlo settings
☒	☐	For hierarchical and complex designs, identification of the appropriate level for tests and full reporting of outcomes
☒	☐	Estimates of effect sizes (e.g. Cohen's *d*, Pearson's *r*), indicating how they were calculated

Our web collection on statistics for biologists contains articles on many of the points above.

Software and code

Policy information about availability of computer code

Data collection	No original data was collected for this study, therefore no software was used for data collection.
Data analysis	Analysis code for the manuscript is available at https://github.com/caycogajiclab/sliceTCA_paper. A GPU accelerated Python library for the sliceTCA data analysis pipeline can be found at https://github.com/arthur-pe/slicetca. All code is based on Python 3.8.

For manuscripts utilizing custom algorithms or software that are central to the research but not yet described in published literature, software must be made available to editors and reviewers. We strongly encourage code deposition in a community repository (e.g. GitHub). See the Nature Portfolio guidelines for submitting code & software for further information.

Data

Policy information about availability of data

All manuscripts must include a data availability statement. This statement should provide the following information, where applicable:

- Accession codes, unique identifiers, or web links for publicly available datasets
- A description of any restrictions on data availability
- For clinical datasets or third party data, please ensure that the statement adheres to our policy

For this study we analyzed three datasets: 1) an openly available motor cortical dataset during reaching from the Neural Latents Benchmark (https://neurallatents.github.io/) 2) an openly available Neuropixel dataset from mice during perceptual decision making from the International Brain Laboratory (https://www.internationalbrainlab.com/data), and 3) a previously published cerebello-cortical imaging dataset generously provided by Dr Mark Wagner (https://

doi.org/10.1016/j.cell.2019.02.019). Source data is available at: https://github.com/caycogajiclab/sliceTCA_paper and the data for the analyzed sessions are available on Figshare: https://figshare.com/articles/dataset/Pellegrino_Stein_Cayco-Gajic_2024_Nat-Neuro/24961917/1

Human research participants

Policy information about studies involving human research participants and Sex and Gender in Research.

Reporting on sex and gender	N/A
Population characteristics	N/A
Recruitment	N/A
Ethics oversight	N/A

Note that full information on the approval of the study protocol must also be provided in the manuscript.

Field-specific reporting

Please select the one below that is the best fit for your research. If you are not sure, read the appropriate sections before making your selection.

☒ Life sciences ☐ Behavioural & social sciences ☐ Ecological, evolutionary & environmental sciences

For a reference copy of the document with all sections, see nature.com/documents/nr-reporting-summary-flat.pdf

Life sciences study design

All studies must disclose on these points even when the disclosure is negative.

Sample size	Since we re-analyzed existing data, no statistical method was used to predetermine sample size. Instead, we demonstrated the utility of our analyses and sliceTCA by choosing three previously published datasets representing a typical range of numbers of recorded neurons, timepoints, and trials as used in the field (Churchland et al., 2012; Wagner et al., 2019; IBL et al., 2022).
Data exclusions	For the IBL dataset, we excluded neurons with very low firing rates (<0.2 Hz).
Replication	To ensure reproducible findings, we have released all data analysis code on GitHub: https://github.com/caycogajiclab/sliceTCA_paper In addition, a GPU accelerated Python library for the sliceTCA data analysis pipeline is available as a pip-installable package at: https://github.com/arthur-pe/slicetca
Randomization	During cross-validation, tensor entries (indexed by neurons, trials and blocks of time) were randomly allocated (80-20%) into training vs. held-out data using a pseudo-random number generator.
Blinding	No blinding was performed for cross-validation, as the analyses were performed automatically through Python scripts.

Reporting for specific materials, systems and methods

We require information from authors about some types of materials, experimental systems and methods used in many studies. Here, indicate whether each material, system or method listed is relevant to your study. If you are not sure if a list item applies to your research, read the appropriate section before selecting a response.

Materials & experimental systems

n/a	Involved in the study	
☒	☐	Antibodies
☒	☐	Eukaryotic cell lines
☒	☐	Palaeontology and archaeology
☒	☐	Animals and other organisms
☒	☐	Clinical data
☒	☐	Dual use research of concern

Methods

n/a	Involved in the study	
☒	☐	ChIP-seq
☒	☐	Flow cytometry
☒	☐	MRI-based neuroimaging
